# Evolution of resistance under alternative models of selective interference

**DOI:** 10.1111/jeb.13919

**Published:** 2021-09-25

**Authors:** Philip G. Madgwick, Ricardo Kanitz

**Affiliations:** ^1^ Syngenta Jealott’s Hill International Research Centre Bracknell UK; ^2^ Syngenta Crop Protection Basel Switzerland

**Keywords:** genetics of adaptation, Hill‐Robertson interference, pesticide or drug mixtures, population genetics, resistance management

## Abstract

The use of multiple pesticides or drugs can lead to a simultaneous selection pressure for resistance alleles at different loci. Models of resistance evolution focus on how this can delay the spread of resistance through a population, but often neglect how this can also reduce the probability that a resistance allele spreads. This neglected factor has been studied in a parallel literature as selective interference. Models of interference use alternative constructions of fitness, where selection coefficients from different loci either add or multiply. Although these are equivalent under weak selection, the two constructions make alternative predictions under the strong selection that characterizes resistance evolution. Here, simulations are used to examine the effects of interference on the probability of fixation and time to fixation of a new and strongly beneficial mutation in the presence of another strongly beneficial allele with variable starting frequency. The results from simulations show a complicated pattern of effects. The key result is that, under multiplicativity, the presence of the strongly beneficial allele leads to a small reduction in the probability of fixation for the new beneficial mutation up to ~10%, and a negligible increase in the average time to fixation up to ~2%, whereas under additivity, the effect is more substantial at up to ~50% for the probability of fixation and ~100% for the average time to fixation. Consequently, the effect of interference is only an important feature of resistance evolution under additivity. Current evidence from studies of experimental evolution provides widespread support for the basic features of additivity, which suggests that interference may afford resistance a different pattern of evolution than other adaptations: rather than the gradual and simultaneous selection of many alleles with small effects, the rapid evolution of resistance may involve the sequential selection of alleles with large effects.

## 1 INTRODUCTION

The evolution of resistance is a major threat to the control of pests, weeds and pathogens with implications for food security and human health (Gould et al., 2018; Powles & Yu, 2010; World Health Organization, 2014). Although it may not always be possible to prevent resistance, evolutionary theory can be applied to manage the evolution of resistance to delay its spread (Curtis et al., 1993; Georghiou, 1994; Roush, 1989; Tabashnik, 1989). One of the principal strategies of resistance management is to use two (or more) active ingredients over a period of time in pesticide or drug combinations, often simply implemented as ‘mixtures’. A mixture differs from other strategies of resistance management (e.g. rotations or mosaics) in providing a simultaneous selection pressure (Comins, 1986; Curtis, 1985; Mani, 1985) that provides ‘redundant kill’: a mutant individual that receives a dose of two active ingredients may be resistant to one of them, but it is nonetheless susceptible to the other, which can delay the evolution of resistance by reducing the fitness of such single mutants (Comins, 1986; Georghiou, 1994; Helps et al., 2020; Slater et al., 2017).

The effect of mixtures on the spread of resistance through a population critically depends upon the origins of mutations that confer resistance (see Hawkins et al., 2019 for a review). In smaller populations, we might expect resistance evolution to be limited by the rate of *de novo* mutation—and there is some supporting evidence of this (Rousselle et al., 2020). In this context, despite a simultaneous selection pressure for independent resistance mutations at different loci, the evolution of resistance may occur through the spread of resistance alleles at different loci in nonoverlapping time‐windows because selection leads to the fixation of a resistance allele faster than new resistance alleles may arise by mutation (see also Kreiner et al., 2018). In larger populations that are more characteristic of pests, weeds and pathogens, resistance alleles may exist in the standing variation, which may reflect the drift of neutral variants before the selection for resistance or the historical selection for resistance within a population, or may spread (or introgress) from other populations with historical selection for resistance (Hawkins et al., 2019). The use of an active ingredient within the population prior to the formulation of the mixture is a common practice due to the costliness of developing multiple new active ingredients. The pre‐existence of resistance alleles in the standing variation is a common assumption in models of resistance evolution, which restricts focus to the deterministic role of selection on the *time* it takes for a resistance mutation to spread through a population and excludes the role of stochastic factors like genetic drift that affect the *probability* that a given resistance mutation may spread (see REX Consortium, 2010 for a meta‐analysis of resistance models). Excluding stochastic factors could be of little consequence to the comparison of resistance‐management strategies if their effects on resistance evolution are constant irrespective of the strategy, but this would seem unlikely for mixtures in comparison to other strategies with insights from a separate literature on ‘selective interference’.

Outside of the context of resistance evolution, the effect of simultaneous selection pressures has been intensely studied as selective interference that reduces the probability of fixation (Barton, 1995; Felsenstein, 1974; Felsenstein & Yokoyama, 1976; Hill & Robertson, 1966; Kim & Stephan, 2003; Neher et al., 2013; Neher & Shraiman, 2009; Otto & Barton, 1997; Weissman & Barton, 2012; Weissman & Hallatschek, 2014; Yu & Etheridge, 2010). Interference arises due to the chance association between a mutation and other alleles (Felsenstein, 1974; Fisher, 1930) and is the root cause of diverse phenomena, such as the intrinsic evolutionary advantage of sex (Muller, 1932), the rate limit on selective sweeps (Haldane, 1957), hitchhiking by neutral alleles (Maynard Smith & Haigh, 1974), background selection from deleterious alleles (Charlesworth et al., 1993), clonal competition in asexual populations (Gerrish & Lenski, 1998) and genetic ‘draft’ in infinite populations (Gillespie, 2000). Yet, different studies of interference have used alternative fitness models, which has corresponding implications for the predicted effect of interference on the probability of fixation. Critically, when a haploid individual has one copy of a beneficial allele (A,B *vs* a,b) at two loci, the selection coefficients of the beneficial alleles ($s_{A}$, $s_{B}$) can either multiply ($\omega_{\text{AB}}=\left(1+s_{A}\right)\;\left(1+s_{B}\right)$) or add ($\omega_{\text{AB}}=1+s_{A}+s_{B}$; see Table 1). Although it is potentially overly simplistic to find trends across studies with variable set‐ups, theoretical studies that add selection coefficients tend to find a larger reduction in the probability of fixation (Birky & Walsh, 1988; Hill & Robertson, 1966; Lessard & Kermany, 2012; Li, 1987; McVean & Charlesworth, 2000; Robertson, 1961), whereas studies that multiply selection coefficients tend to find a smaller effect (Barton, 1995; Felsenstein, 1974; Felsenstein & Yokoyama, 1976; Kim & Stephan, 2003; Lessard & Kermany, 2012; Li, 1987; Maynard Smith & Haigh, 1974; Neher et al., 2013; Otto & Barton, 1997; Weissman & Barton, 2012; Weissman & Hallatschek, 2014; Yu & Etheridge, 2010). For example, Hill and Robertson (1966) found that selective interference may reduce the probability of fixation by >50% when selection coefficients are both large under additivity, whereas Barton (1995) found that it may only reduce the probability of fixation by <10% in this set‐up under multiplicativity. The two models also differ in other ways: the model in Hill and Robertson (1966) involves pre‐existing alleles in the absence of mutation with results derived from a Monte Carlo simulation procedure, whereas the model in Barton (1995) involves recurrent mutation with results derived from numerical evaluation of analytical derivations. Where the potential for differences between multiplicative and additive set‐ups has been acknowledged in the interference literature (Lessard & Kermany, 2012; Li, 1987; McVean & Charlesworth, 2000; Neher et al., 2013; Neher & Shraiman, 2009; Otto & Barton, 1997; Yu & Etheridge, 2010), studies have restricted their attention to weak selection, where there is little to distinguish multiplicativity and additivity (because $\left(1+s_{A}\right)\;\left(1+s_{B}\right)\approx 1+s_{A}+s_{B}$ are close to zero) such that this difference can be ignored to justify whichever set‐up is simpler for the chosen analysis. Yet, the assumption of weak selection does not make sense in the context of resistance evolution. The application of a pesticide or drug provides strong selection, with resistance mutations having distinctively larger selection coefficients than other forms of adaptation in natural populations (Reznick & Ghalambor, 2001; Thurman & Barrett, 2016). Therefore, it is critical to resolve whether selection coefficients should multiply or add to examine the effects of interference on the evolution of resistance.

**TABLE 1:** Differences in the fitness model under multiplicativity, additivity and resistivity, and its impact of the delta equation for the change in allele frequency at each locus

	Multiplicativity	Additivity	Resistivity
$\left(1+s_{A}\right)\;\left(1+s_{B}\right)$	$1+s_{A}+s_{B}$	$1‐d\;\left(1‐s_{A}\right)\;\left(1‐s_{B}\right)$
$\omega_{\text{Ab}}=$	$1+s_{A}$	$1+s_{A}$	$1‐d\;\left(1‐s_{A}\right)$
$\omega_{\text{aB}}=$	$1+s_{B}$	$1+s_{B}$	$1‐d\;\left(1‐s_{B}\right)$
$\omega_{\text{ab}}$	1	1	1‐d
$\Delta f_{A}=$	$\frac{f_{A}\left(1‐f_{A}\right)\;s_{A}}{1+f_{A}s_{A}}$	$\frac{f_{A}\left(1‐f_{A}\right)\;s_{A}}{1+f_{A}s_{A}+f_{B}s_{B}}$	$\frac{df_{A}\left(\left(1‐f_{A}\right)s_{A}‐f_{B}s_{B}\left(1‐f_{A}s_{A}\right)\right)}{1‐d\left(1‐f_{A}s_{A}\right)\left(1‐f_{B}s_{B}\right)}$
$\Delta f_{B}=$	$\frac{f_{B}\left(1‐f_{B}\right)\;s_{B}}{1+f_{B}s_{B}}$	$\frac{f_{B}\left(1‐f_{B}\right)\;s_{B}}{1+f_{A}s_{A}+f_{B}s_{B}}$	$\frac{df_{B}\;\left(\left(1‐f_{B}\right)\;s_{B}‐f_{A}s_{A}\;\left(1‐f_{B}s_{B}\right)\right)}{1‐d\;\left(1‐f_{A}s_{A}\right)\;\left(1‐f_{B}s_{B}\right)}$

Note

These models assume that individuals are haploid with one copy of a beneficial allele (A/B) at two loci (where beneficial allele A competes against wildtype allele a at locus Aa and beneficial allele B competes against wildtype allele b at locus Bb), the selection coefficients of the beneficial alleles ($s_{A}/s_{B}$) can either add ($\omega_{B}=1+s_{A}+s_{B}$) or multiply ($\omega_{\mathrm{AB}}=1+s_{A}+s_{B}$). In the case of resistivity, a mixture of pesticides are assumed to have a mortality effect (d) that it mitigated by the beneficial resistance alleles (at rate $1‐s_{A}$ and $1‐s_{B}$ for alleles A and B respectively). When considering two beneficial resistance alleles, it is possible to recapitulate the exact properties of multiplicativity or additivity by assuming that each beneficial allele provides resistance to separate parts of the mortality effect ($d_{A}$, $d_{B}$), which would lead to the contrast: $\omega_{\mathrm{AB}}=\left(1‐d_{A}\left(1‐s_{A}\right)\right)\left(1‐d_{B}\left(1‐s_{B}\right)\right)$ and $\omega_{\mathrm{AB}}=1‐d_{A}\left(1‐s_{A}\right)‐d_{B}\left(1‐s_{B}\right)$ for multiplicativity and additivity, respectively, but this does not capture new properties in the delta equations.

Multiplicativity and additivity are the basic fitness models for considering simultaneous selection pressures in different modelling traditions (Miller et al., 2018; Orr, 2005; Wade et al., 2001), but these models carry different ramifications. The fundamental difference between these fitness models is that addition implies that an allele has a constant selection coefficient irrespective of its background fitness, whereas multiplication implies that an allele has a constant selection coefficient in any background. Consequently, addition differs because the effective selection coefficient of an allele (as a percentage improvement on mean fitness) reduces as mean fitness increases. In most population genetic models (see Wade et al., 2001), selection coefficients from alleles at the same locus add whilst selection coefficients from alleles at different loci multiply, such that a deviation from adding intralocus selection coefficients would imply a dominance effect, whereas a deviation from multiplying interlocus selection coefficients would imply an epistatic effect. Although there is precedent for adding interlocus selection coefficients (especially in quantitative genetics, as discussed in Wade et al., 2001; see also Miller et al., 2018), other possibilities to this set‐up are rarely considered (though see Barton, 1998; Hartl & Taubes, 1998; Orr, 1999) because it does tend to make modelling simpler: selection can be described assuming haploidy (rather than needing to consider diploidy; sometimes referred to as ‘genic selection’) and the change in allele frequency at one locus can ignore the effects of selection at other loci (under linkage equilibrium; as a form of ‘independence’). Such convenient mathematical properties of adding intralocus selection coefficients and multiplying interlocus selection coefficients may make modelling easier, but this does not justify whether the multiplication or addition is more appropriate.

Given their ramifications, the biological meaning of multiplicativity and additivity is not as straight‐forward as it is sometimes made out by proponents who can only see the logic of one fitness model. Consider the evolution of resistance to a mixture of two pesticides that each independently afford 90% mortality. The mixture might be (multiplicatively) assumed to have 99% mortality, but the interaction between pesticides could have synergistic (or antagonistic) effects on mortality from using the pesticides together, which would mean that a resistance mutation may have a lesser or greater effect than expected from their independent mortalities. Further, a resistance mutation might be assumed to confer a 100% increase in survival to one pesticide, but it may also confer some level of cross‐resistance to the other pesticide. Although this might seem unlikely for *de novo* target‐site mutations that alter pesticide binding in unrelated proteins, this may be more likely with resistance mutations that lead to metabolic, physiological or behavioural changes (e.g. Beckie & Tardif, 2012; Yunta et al., 2019) that may exist in the standing variation (owing to reduced costliness). One way to conceptualize this distinction is as separate trait dimensions (Fisher, 1930; Orr, 1998, 1999, 2000), where independent traits that are encoded at separate loci would have multiplicative selection coefficients as their beneficial alleles are improving unrelated aspects of survival or fecundity, whereas selection coefficients from alleles at the same locus would add because they affect the same trait. This raises the almost‐philosophical question of whether resistance to a mixture of pesticides counts as one or two traits; the question is more tangible when considering that this is what factors like synergism and cross‐resistance are really blurring. Herein, it is critical to recognize that the addition of selection coefficients may not accurately reflect the interaction between selection coefficients that these factors imply, but it is not the ‘special case’ where beneficial alleles evolve independently. Instead, additivity is a basic representative of nonmultiplicative fitness models where selection coefficients are affected by the shifting baseline of mean fitness. For example, a mixture of pesticides might have a mortality effect (d) that it mitigated by beneficial resistance alleles at rate $1‐s_{A}$ and $1‐s_{B}$ for alleles A and B, respectively, such that the selection coefficients for each allele neither add nor multiply but rather: $\omega_{\text{AB}}=1‐d\;\left(1‐s_{A}\right)\;\left(1‐s_{B}\right)$ (see ‘Resistivity’ in Table 1 for the effect on mean fitness). That said, the addition of selection coefficients could represent a plausible model of the interaction between selection coefficients in its own right, implying that an allele leads to a constant improvement on fitness irrespective of mean fitness. Therefore, we can recognize multiplicativity and additivity (and resistivity) as ‘valid’ models with different implications, and whichever implication best reflects biological examples in particular or in general is to be settled by empirical (not theoretical) research.

In summary, in the context of resistance evolution to the simultaneous and strong selection pressure from using multiple active ingredients in pesticide or drug mixtures, multiplication and addition are two basic fitness models for the interaction between selection coefficients at different loci that present alternative set‐ups in a constant or shifting baseline of mean fitness, respectively, which may have implications for the effects of selective interference on the spread of resistance alleles. The aim of this paper is two‐fold. First, there is a fundamental question about whether multiplicative and additive set‐ups are sufficient to explain the difference that is observed in the effect of interference on the probability of fixation in studies that differ in many other ways. Second, there is a more applied question about whether the neglect of genetic drift in resistance models removes an important aspect of resistance evolution by ignoring effects on the probability of resistance from interference, which may differ under multiplicativity and additivity. Here, we use simulations to address these questions to describe the relative change in the probability of and time to fixation of a new and strongly beneficial mutation (with a variable selection coefficient) that occurs during an ongoing substitution of another strongly beneficial allele (with a variable selection coefficient and starting frequency) under multiplicativity or additivity. In comparison with other recent studies that quantify the effects of interference in models with recurrent sweeps (Barton, 1994; Neher et al., 2013; Neher & Shraiman, 2011; Weissman & Barton, 2012; Weissman & Hallatschek, 2014), the simulations presented here focus on the interaction between simultaneous selection pressures and genetic drift in the absence of mutation, which affords statistics on the probability of and time to fixation of a new mutation that are comparable to classic estimates (Kimura, 1957, 1962) without simultaneous selection pressures.

## 2 METHODS

The probability of fixation and average time to fixation of a new mutation has been extensively studied in a simple two‐allele system within a Wright‐Fisher idealized population (Fisher, 1930; Wright, 1931), which assumes a finite census population size N (where $N=N_{e}$ by definition), genic selection, a negligible mutation rate, random mating, nonoverlapping generations, unstructured populations and so on (Kimura, 1957, 1962). For correspondence, this set‐up is also used here. Simulations can be used to calculate the summary statistics about a new mutation in this set‐up by iteratively sampling an allele into the next generation at a rate equal to its frequency after selection, which is described by a binomial distribution; in each generation, the probability distribution of allele frequencies in the next generation is calculated by weighting each of the binomial distributions (that describe the probability distributions in the next generation from a particular allele frequency after selection) by the probability of a population having the new mutation at that given frequency after selection. In principle, it would be possible to formulate an equivalent two‐allele system with the inclusion of a second beneficial allele at another locus using a multinomial distribution, but this has too large a computational burden with the consideration of linkage disequilibrium. As a result, previous simulations have resorted to averaging across multiple runs of different population instances to derive an approximation of the probability of fixation of a new mutation (Birky & Walsh, 1988; Felsenstein & Yokoyama, 1976; Hill & Robertson, 1966; Kim & Stephan, 2003; McVean & Charlesworth, 2000; Neher et al., 2013; Pearce & Fisher, 2019; Weissman & Barton, 2012; Yu & Etheridge, 2010). Here, instead, the evolution of the entire probability distribution of allele frequencies is tracked, resulting in high‐quality data that are noiseless and reproducible, but also giving access to an additional summary statistic in the average time to fixation of a mutation. Accordingly, two computationally tractable simulations are run, which are termed stochastic and deterministic. In the stochastic simulations, both loci are sampled according to independent binomial distributions, capturing the effects of linkage disequilibrium on the frequencies of alleles but ignoring the effects of linkage disequilibrium on the sampling process itself. This set‐up is more computationally efficient than a multinomial sampling process, which enables larger population sizes to be simulated. In the deterministic simulations, the first locus is sampled from a binomial distribution and the second locus has the beneficial allele increase in the manner of a discrete‐time model of selection, ignoring the change in allele frequencies from drift at the second locus. The deterministic simulations are more computationally efficient than the stochastic simulations and so enable even larger population sizes to be simulated. Both these simulations are presented in the results and supplement, but note that these two approximations are congruent except when there is both near‐perfect linkage disequilibrium, whereupon the sampling process can never separate a mutation from its background, and concurrently, there is a very low initial frequency of the second beneficial allele, whereupon its drift is important to its evolution.

Simulations are set up for the two basic fitness models of additivity and multiplicativity (Table 1) with the results presented in the main‐text, whereas the resistivity results are presented in the supplement. At the first locus, a single copy of a new beneficial mutation (allele ) with a particular selection coefficient ($s_{A}$) arises and competes against a wildtype (allele a). At the second locus, a beneficial mutation (allele B) with a particular selection coefficient ($s_{B}$) and frequency ($f_{B}$) arises and competes against a wildtype (allele b). The beneficial mutation that produces allele A can arise on a background with allele B or b with a probability equal to the frequency of either allele ($f_{B}$ and $1‐f_{B}$, respectively). Allele A is associated with its background, which is maintained between the generations at a rate equal to a correlation coefficient (r) (Birky & Walsh, 1988) that enables linkage disequilibrium to be built by selection: when r=0 there is no association between an allele and its background between the generations and when r=1 an allele is co‐inherited with its background. Accordingly, the correlation coefficient runs on a reverse scale to the recombination coefficient that other studies have used, but the correlation coefficient is more tangible here because it describes the probability of the co‐inheritance of the alleles (that also provides r=0 with a meaning to describe the assumption of infinite population size). Simulations are run in a factorial design across parameter space of all combinations of: the selection coefficient $s_{B}$ and initial frequency $f_{B}$ of an existing beneficial allele B at another locus, the selection coefficient for a new mutation $s_{A}$ (with initial frequency $f_{A}=1/N$), the correlation coefficient (r) and for both additivity and multiplicativity. Due to computational load, simulations are run for the largest population size possible given the available computing facilities of N=100 for the stochastic simulations and $N=\left\{100,\;1000\right\}$ for the deterministic simulations. As such population sizes are smaller than many pest populations, larger selection coefficients (s≥0.1) are used to account for the drift‐selection balance because of the drift barrier when: s<1/N (Kimura, 1968; Li, 1978). Each simulation runs for a maximum of 10N generations, but a simulation usually ends before then when 99.999% of the probability distribution of frequencies for the allele A has become fixed or lost (and the percentage completion of each run is recorded). For each simulation, data are recorded on: the probability of fixation (and loss) and the average time to fixation (and loss) for the new beneficial mutation that creates allele A when arises on a background with allele B or allele b (and, for the stochastic simulation, the same statistics for the allele B). The overall statistics are calculated by taking the weighted‐sum of the statistic for either background (B or b), weighting by the probability that mutation A occurs on either background (which depends on the initial frequency of the two alleles at the second locus: $f_{B}$ and $1‐f_{B}$, respectively). In total, 144 900 simulations were run for all the combinations of parameters with each taking around ~30 min (0.5 * 144 900 = 72 450 CPU h or ~8.2 CPU years) and requiring up to 160 GB of memory.

The range of parameters is chosen to be representative of the parameter space, but the correlation coefficient is also varied to simulate two kinds of interference, which are the extremes on a continuum that depends on the quantity of recombination. First, in‐keeping with the primary interest of this paper, there is interlocus interference where the selection of an allele at one locus alters the selection of an allele at another locus. The correlation coefficient (r) for the two alleles may vary depending on whether or not the two alleles are on the same chromosome, but for all other cases is expected to be low because of the free recombination of nonhomologous chromosomes. Theoretically, under random mating, models with infinite population sizes necessarily assume r=0 because alleles are inherited independently, whereas models of finite population sizes assume r=1/2 because alleles have a 50% chance of being inherited together; simulations are conducted for both parameters but the main‐text results focus on r=1/2. Factors like nonrandom mating, overlapping generations and population structure can lead to inbreeding, which violates the assumption of random mating and can lead to a larger correlation coefficient (represented by r=0.75 and 0.9 in the figures in the supplement). Yet, logically, only asexuality leads to a correlation coefficient that approaches the extreme where two alleles are only ever found together (r=1) because the association between a new mutation and its background loci is described by $r^{t}$ where t is generation time (so for r<1, $r^{t}$ quickly tends towards zero unless r≈1). Second, at this extreme, there is intralocus interference where the selection of an allele alters the selection of another allele at the same locus, which is incidentally the only possible form of interference in an asexual population with no recombination (r=1). The correlation coefficient for the two alleles is expected to be very high because of a low intralocus recombination rate, and so fixation entails one allele out‐competing another. Although the accuracy of the simulations breaks down when there is both a very low initial frequency of the existing beneficial allele and near‐perfect linkage disequilibrium, the results are accurate for higher initial frequencies of the existing beneficial allele and are nonetheless qualitatively indicative at this extreme as a conservative estimate.

## 3 RESULTS

The results of both stochastic and deterministic scenarios for the second locus show the same pattern (compare Figure S1‐S12). Further, for all initial frequencies of allele B except when the initial frequency is very small, the stochastic scenario shows that allele B has a near 100% probability of fixation and an average time to fixation that is well approximated by deterministic dynamics (Figure S13‐S16). Therefore, here in the main text, the higher resolution deterministic results are treated as representative of all the results (for direct comparison, see Figure S17‐S20).

**FIGURE 1:**
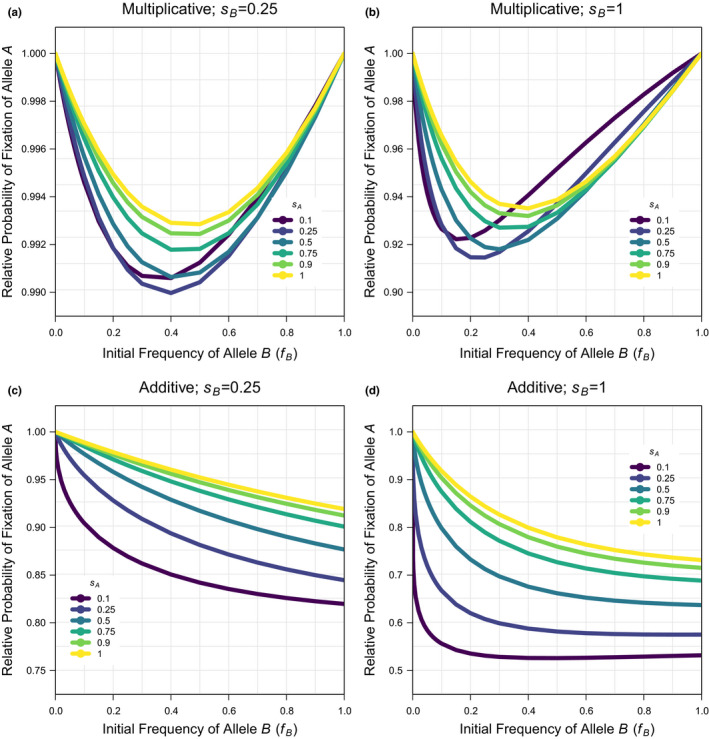
The probability of fixation of a new beneficial mutation when there is an ongoing substitution of an existing beneficial allele at another locus relative to the probability of fixation of a new beneficial mutation in isolation under interlocus interference. Simulation data are used for the deterministic scenario (with population size: N=1000) across a subset of the full parameter space (see Figure S1‐S6 for comparison and Data‐file S3 & S6 for the raw data) for: the frequency of the existing beneficial allele ($f_{B}=\left\{0‐1\right\}$ in 22 values; x‐axis of plots), the selection coefficient of the new beneficial mutation ($s_{A}=\left\{0.1,0.25,0.5,0.75,0.9,1\right\}$; coloured lines per plot), the selection coefficient of the existing beneficial allele ($s_{B}=\left\{0.25,1\right\}$; panel columns) and the fitness model (Multiplicative or Additive; panel rows) when the correlation coefficient among the new and existing beneficial alleles is at its minimum in finite populations (r=0.5)

**FIGURE 2:**
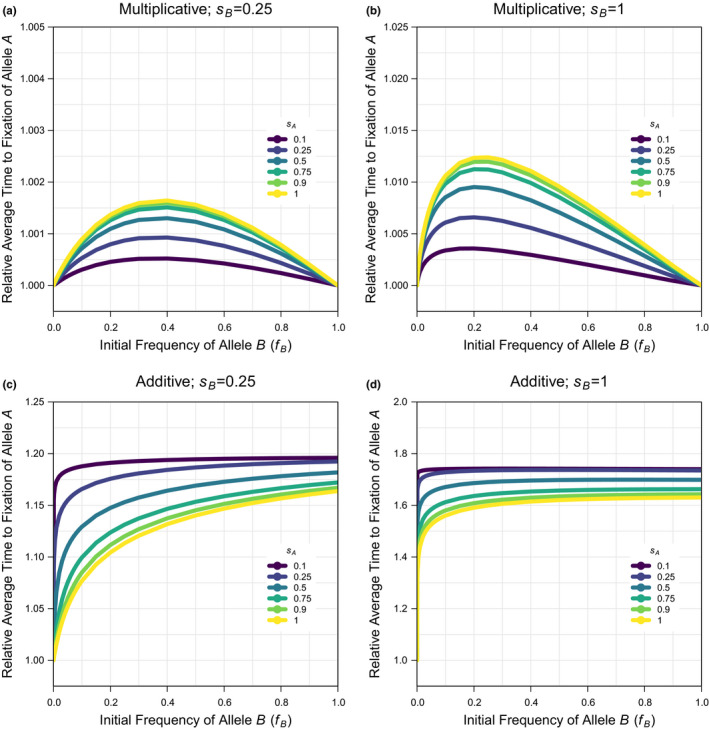
The average time to fixation of a new beneficial mutation when there is an on‐going substitution of an existing beneficial allele at another locus relative to the average time to fixation of a new beneficial mutation in isolation under interlocus interference. Simulation data are used for the deterministic scenario (with population size: N=1000) across a subset of the full parameter space (see Figure S7‐S12 for comparison and Data‐file S3 & S6 for the raw data) for: the frequency of the existing beneficial allele ($f_{B}=\left\{0‐1\right\}$ in 22 values; x‐axis of plots), the selection coefficient of the new beneficial mutation ($s_{A}=\left\{0.1,0.25,0.5,0.75,0.9,1\right\}$; coloured lines per plot), the selection coefficient of the existing beneficial allele ($s_{B}=\left\{0.25,1\right\}$; panel columns) and the fitness model (Multiplicative or Additive; panel rows) when the correlation coefficient among the new and existing beneficial alleles is at its minimum in finite populations (r=0.5)

**FIGURE 3:**
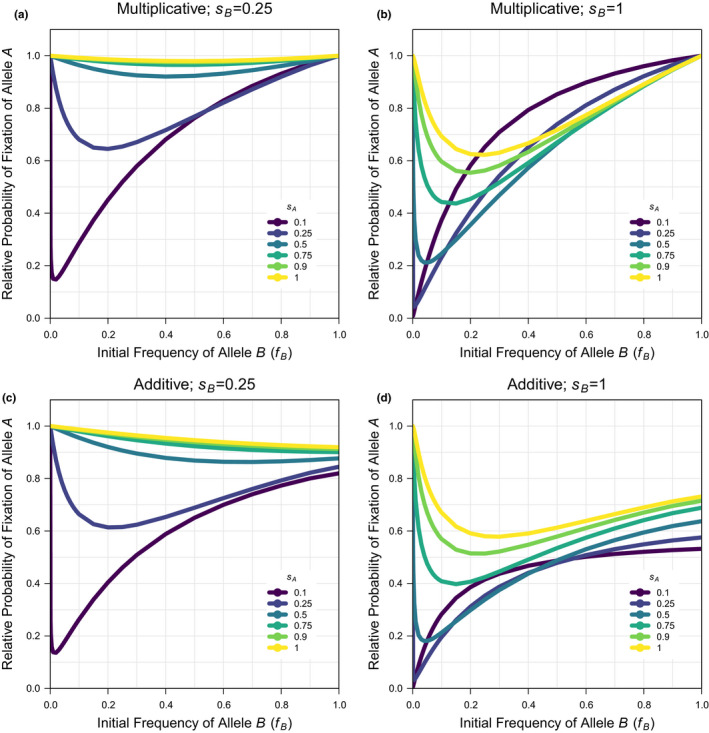
The probability of fixation of a new beneficial mutation when there is an ongoing substitution of an existing beneficial allele at another locus relative to the probability of fixation of a new beneficial mutation in isolation under intralocus interference. Simulation data are used for the deterministic scenario (with population size: N=1000) across a subset of the full parameter space (see Figure S1‐S6 for comparison and Data‐File S3 & S6 for the raw data) for: the frequency of the existing beneficial allele ($f_{B}=\left\{0‐1\right\}$ in 22 values; x‐axis of plots), the selection coefficient of the new beneficial mutation ($s_{A}=\left\{0.1,0.25,0.5,0.75,0.9,1\right\}$; coloured lines per plot), the selection coefficient of the existing beneficial allele ($s_{B}=\left\{0.25,1\right\}$; panel columns) and the fitness model (Multiplicative or Additive; panel rows) when the correlation coefficient among the new and existing beneficial alleles is at its maximum (r=1)

**FIGURE 4:**
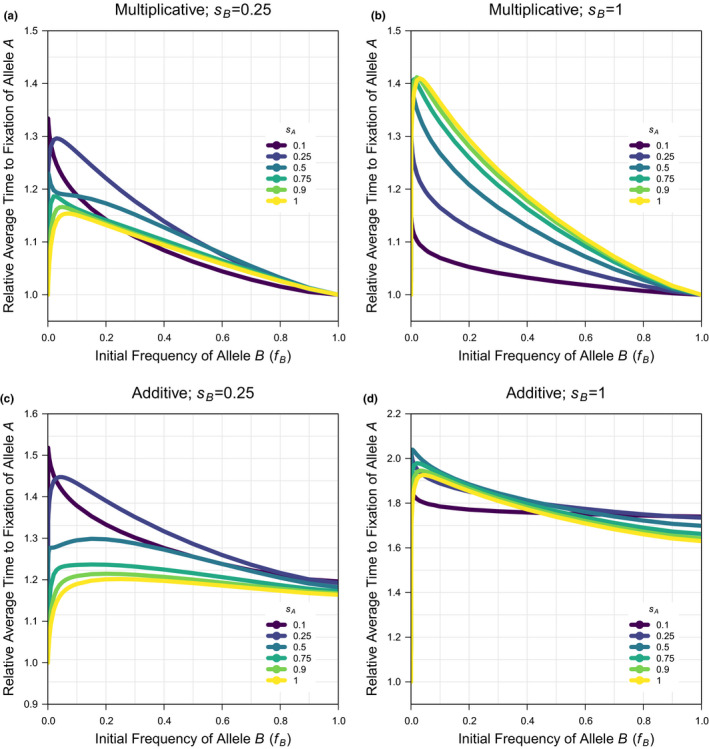
The average time to fixation of a new beneficial mutation when there is an on‐going substitution of an existing beneficial allele at another locus relative to the average time to fixation of a new beneficial mutation in isolation under intralocus interference. Simulation data are used for the deterministic scenario (with population size: N=1000) across a subset of the full parameter space (see Figure S7‐S12 for comparison and Data‐File S3 & S6 for the raw data) for: the frequency of the existing beneficial allele ($f_{B}=\left\{0‐1\right\}$ in 22 values; x‐axis of plots), the selection coefficient of the new beneficial mutation ($s_{A}=\left\{0.1,0.25,0.5,0.75,0.9,1\right\}$; coloured lines per plot), the selection coefficient of the existing beneficial allele ($s_{B}=\left\{0.25,1\right\}$; panel columns) and the fitness model (Multiplicative or Additive; panel rows) when the correlation coefficient among the new and existing beneficial alleles is at its maximum (r=1)

The probability of fixation of allele A when allele B is not present ($f_{B}=0$) is the same under additivity and multiplicativity and can be predicted for the selection coefficient for allele A as: $\pi =1‐\exp\;\left(‐2s_{A}\right)$ (Kimura, 1957, 1962); this approximation is accurate for small to intermediate selection coefficients, but needs to be corrected by a constant factor (k≈0.9, fitted statistically; see Script 4 in the supplement) to be accurate for the larger selection coefficients that are considered in the simulations here (because of the interest in more strongly beneficial alleles and the computation constraints on low population size). When the correlation coefficient is not one, the simulations describe scenarios of interlocus interference. Under multiplicativity, there is a modest reduction in the probability of fixation under a low correlation coefficient that generates loose linkage (Figure 1a,b, Figure S1‐S3). By explicitly showing the relationship across initial frequencies of allele $B\;(f_{B})$, the data here also show that the maximum effect of allele B on the probability of fixation of allele A occurs for large selection coefficients ($s_{B}$) and intermediate initial frequencies ($f_{B}$), and is small at <10%. Under additivity, there is a larger reduction in the probability of fixation under a low correlation coefficient that generates loose linkage (Figure 1c,d, Figure S4‐S6). Unlike under multiplicativity, the probability of fixation under additivity is unequal at the intercepts $f_{B}=0$ and, $f_{B}=1$ leading to a maximum effect of <50% when there is a large asymmetry in frequency and/or selection coefficient between alleles A and B. Furthermore, there is a large reduction in the probability of fixation of a new mutation with even a marginal asymmetry in initial allele frequencies. Consistent with the results on the probability of fixation, the average time to fixation shows a more negligible increase under multiplicativity (Figure 2a,b, Figure S7‐S9) but a more substantial increase in the average time to fixation with even a marginal asymmetry in initial allele frequencies under additivity (Figure 2c,d, Figure S10‐S12). When the correlation coefficient is near one, the simulations describe intralocus interference because a new mutation is exclusively associated with the background it arises on. The results are similar under multiplicativity (Figure 3a,b, Figure S1‐S3) and additivity (Figure 3c,d, Figure S4‐S6), showing a large reduction in the probability of fixation, which can be >95%, when there is a small asymmetry in frequency and/or a large asymmetry in selection coefficient between alleles A and B. Consistent with these results, the average time to fixation similarly increases but with different extents of <40% under multiplicativity (Figure 4a,b, Figure S7‐S9) and <100% under additivity (Figure 4c,d, Figure S10‐S12). In summary, the results show that interlocus interference produces a more negligible effect under multiplicativity and a more substantial effect under additivity, whereas intralocus interference produces a similarly substantial effect under both multiplicativity and additivity.

For interlocus interference in sexual populations, the probability of fixation and the average time to fixation of a new beneficial mutation can be approximated using effective selection coefficients, which are calculated as the percentage improvement on mean fitness at the time of mutation ($1+f_{B}s_{B}$). Each statistic is calculated as the sum of the statistic for either background (B or b) weighted by the probability that mutation A occurs on either background (which depends on the initial frequency of the two alleles: $f_{B}$ and $1‐f_{B}$, respectively). Consequently, under multiplicativity, the two probabilities of fixation when mutation A arises on either background (given as $\pi_{A|B}$ and $\pi_{A|b}$) and the overall probability of fixation are (cf. Kimura, 1957):

(1a) $$\pi_{A|B}\approx k\left(1‐\exp\left(\frac{‐2s_{A}\left(1+s_{B}\right)}{\left(1+f_{B}s_{B}\right)}\right)\right)$$

(1b) $$\pi_{A|b}\approx k\left(1‐\exp\left(\frac{‐2s_{A}}{\left(1+f_{B}s_{B}\right)}\right)\right)$$

(1c) $$\pi \;\approx \;k\left(1\;‐\;f_{B}\;\exp\;\left(\frac{‐2s_{A}\left(1+s_{B}\right)}{\left(1+f_{B}s_{B}\right)}\right)\;‐\;\left(1‐f_{B}\right)\;\exp\;\left(\frac{‐2s_{A}}{\left(1+f_{B}s_{B}\right)}\right)\right)$$

Alternatively, under additivity, the two probabilities of fixation when mutation A arises on either background (given as $\pi_{A|B}$ and $\pi_{A|b}$) are:

(2a) $$\pi_{A|B}\approx k\left(1‐\exp\;\left(\frac{‐2\left(s_{A}+\left(1‐f_{B}\right)s_{B}\right)}{\left(1+f_{B}s_{B}\right)}\right)\right)$$

(2b) $$\pi_{A|b}\approx k\;\left(1‐\exp\;\left(\frac{‐2\left(s_{A}‐f_{B}s_{B}\right)}{\left(1+f_{B}s_{B}\right)}\right)\right)$$

(2c) $$\pi \approx k\left(1‐f_{B}\exp\left(\frac{‐2\left(s_{A}+\left(1‐f_{B}\right)s_{B}\right)}{\left(1+f_{B}s_{B}\right)}\right)‐\left(1‐f_{B}\right)\exp\left(\frac{‐2\left(s_{A}‐f_{B}s_{B}\right)}{\left(1+f_{B}s_{B}\right)}\right)\right)$$

Under both multiplicativity and additivity, the overall approximation for the probability of fixation (Equation 1c and 2c) is a reasonable prediction of the actual probability of fixation when the correlation coefficient is not near one (Figure S21‐S22). The average time to fixation has a simpler approximation because the average time to fixation of the new beneficial mutation is almost unchanged across the frequency of the existing beneficial allele. Under multiplicativity, the average time to fixation (given as T) is approximated by (cf. Hermisson & Pennings, 2005):

(3) $$T\approx \frac{2\;\ln\;\left(4Ns_{A}\right)}{s_{A}}$$

Under additivity, the case where *f_B_ = 0* is equal to the approximation under multiplicativity but all other cases where *f_B_* > 0 are approximated by:

(4) $$T\approx \frac{2\;\left(1+s_{B}\right)\;\ln\;\left(\frac{4\;Ns_{A}}{1+s_{B}}\right)}{s_{A}}$$

These approximations for the average time to fixation give a reasonably accurate prediction of the actual average time to fixation (Figure S23‐S24), especially when the initial frequency of the existing beneficial allele is not small and the correlation coefficient is not one.

## 4 DISCUSSION

To quantify the effects of interference on the evolution of resistance under multiplicative and additive fitness models, simulations were run to show the change in the probability of fixation and the average time to fixation of a new beneficial mutation when there is an on‐going substitution of a beneficial allele with variable starting frequencies. The results show a complicated set of patterns. For interlocus interference among the beneficial alleles at different loci, larger selection coefficients for the beneficial mutation results in smaller reductions in the probability of fixation, whereas larger selection coefficients for the beneficial allele (that has variable frequency) result in larger reductions. Under multiplicativity, the reduction of the beneficial mutation's probability of fixation is greatest at <10% (compared to in the absence of the beneficial allele) for intermediate frequencies of the beneficial allele; under additivity, the reduction is greatest at higher frequencies at <50%. For the average time to fixation, larger selection coefficients for the beneficial mutation and beneficial allele result in larger increases under multiplicativity, but these changes are small at their greatest extent for intermediate frequencies in tending to be <1% increase. Under additivity, larger selection coefficients for the beneficial mutation results in smaller increases in the average time to fixation, whereas larger selection coefficients for the beneficial allele results in larger increases, which tends to be greatest at higher frequencies at <100% increase. For intralocus interference between beneficial alleles that are (actually or effectively) at the same locus, larger selection coefficients for the beneficial mutation results in smaller reductions in the probability of fixation, whereas larger selection coefficients for the beneficial allele result in larger reductions. The results are similar under multiplicativity and additivity: the reduction of the beneficial mutation's probability of fixation is greatest at >90% with smaller selection coefficients for intermediate frequencies of the beneficial allele with larger selection coefficients. For the average time to fixation, the results also show a similar but complicated pattern under multiplicativity and additivity, where larger selection coefficients for the beneficial allele results in larger increases. Yet, although multiplicativity has a >40% increase at its greatest for low (but nonzero) frequencies of the beneficial allele, additivity often has more than double this increase.

The first aim of this study addresses the fundamental question of whether the difference in fitness model between multiplicativity and additivity is sufficient to explain the observed differences in the effect of interference on the probability of fixation in studies that differ in many other ways. The results here are consistent with previous studies that show a negligible effect of interlocus interference under multiplicativity (Barton, 1994, 1995; Kim & Stephan, 2003; Lessard & Kermany, 2012; Li, 1987; Neher & Shraiman, 2009; Otto & Barton, 1997; Weissman & Barton, 2012; Yu & Etheridge, 2010) and a substantial effect of interference under additivity (Birky & Walsh, 1988; Hill & Robertson, 1966; Lessard & Kermany, 2012; Li, 1987; McVean & Charlesworth, 2000; Robertson, 1961). The substantial effect of interlocus interference with even a minor asymmetry in the frequency of the beneficial mutation and the beneficial allele under additivity is surprising given that the loss of an allele is much more likely if it has low number (not frequency) in the population (Fisher, 1930; Fisher & Ford, 1947; Kimura, 1957, 1962; Wright, 1931), where the baseline of mean fitness for the beneficial mutation is presumably relatively unaffected by the presence of the beneficial allele because it is at such low frequency. Yet, the rate of spread of a rare allele is near‐exponential, which gives a minor asymmetry in frequency a major head‐start, and the association between a mutation and its background may be strong even into higher frequencies under strong selection, resulting in a scenario closer to competition despite beneficial alleles being at different loci. The results here also show a more substantial effect of interference under intralocus interference irrespective of fitness model, which is also consistent with previous studies (Barton, 1995; Charlesworth et al., 1993; Cohen et al., 2005; Felsenstein, 1974; Felsenstein & Yokoyama, 1976; Gerrish & Lenski, 1998; Gillespie, 2000; Kosheleva & Desai, 2013; Maynard Smith & Haigh, 1974; McVean & Charlesworth, 2000; Neher & Shraiman, 2011; Otto & Barton, 1997; Park & Krug, 2013; Pearce & Fisher, 2019; Weissman & Barton, 2012; Weissman & Hallatschek, 2014). In this way, the novel feature of our results here is not the size of the effect of interference, but rather demonstrating how the difference between multiplicativity and additivity is sufficient to explain the difference in the size of the effect of interference.

A point of deviation from the results of previous studies comes in the approximation of the effects of interference. Following Robertson (1961), studies have repeatedly—but not universally (Comeron & Kreitman, 2002)—suggested that interference can be approximated as a reduction in the effective population size as a result of increased genetic drift (Barton, 1994, 1995; Birky & Walsh, 1988; Hill & Robertson, 1966; Neher et al., 2013; Weissman & Barton, 2012). Although this attribution may be useful for approximating the effects of interference on the probability of fixation, it is not clear how to extend this explanation to cover the differential effect of interference under multiplicativity and additivity (and the different patterns in the probability of fixation and the average time to fixation). Further, the reduction in the effective population size does not make sense of an observed pattern from interference of the longer times to fixation in models with deterministic evolutionary dynamics (i.e. without drift; Maynard Smith & Haigh, 1974) and/or models with infinite populations (Gillespie, 2000; Kosheleva & Desai, 2013). Here, instead, the effects of interference on the probability of fixation and average time to fixation are understood as reducing the effective selection coefficient of the new beneficial mutation, which permits a relatively simple and reasonably accurate approximation (where it at least provides a conservative estimate because interference acts in a predictable direction). The reduction in the effective selection coefficient captures how interference quantitatively reduces the efficacy of selection and, in this way, enhances the *relative* power of drift—without having to appeal to more complicated qualitative explanations like ‘allelic traffic’ (Comeron & Kreitman, 2002). This explanation is not in disagreement with previous suggestions (Barton, 1994, 1995; Birky & Walsh, 1988; Hill & Robertson, 1966; Neher et al., 2013; Weissman & Barton, 2012), but it does reflect that a similar shift in the power of drift can be achieved in relative terms by either a reduction in the effective population size or the effective selection coefficient.

The second aim addresses a more applied question of whether the neglect of genetic drift in resistance models removes an important aspect of resistance evolution by ignoring effects on the probability of resistance from interference. The answer depends on whether a multiplicative or additive fitness model is more appropriate to resistance evolution. Under multiplicativity, the effect of interference on the probability of fixation is usually negligible, which means that the probability of fixation is constant irrespective of whether resistance‐management strategies lead to selection at one or two loci. In this case, although models of resistance evolution would miss out on stochasticity, genetic drift does not significantly alter the outcome of selection in the comparison of strategies. In contrast under additivity, the effect of interference on the probability of fixation is usually substantial, which means that a selection pressure for resistance at two loci biases the stochasticity to disfavour the simultaneous spread of resistance at both loci. Herein, the loss of a beneficial resistance mutation is a neglected effect of a simultaneous selection pressure from some resistance‐management strategies, like a mixture of two pesticides, such that only focusing on the time to resistance could undervalue this strategy in comparisons. Therefore, the question of whether multiplicativity or additivity is the more appropriate fitness model for resistance evolution becomes crucial. As previously mentioned (see Introduction), additivity need not accurately reflect the interaction between selection coefficients, as it is a basic representative of any nonmultiplicative fitness model where selection coefficients are affected by the shifting baseline of mean fitness. Consequently, other fitness models often have similar properties to additivity, like the model of resistivity that shows a similar delta equation in Table 1. This also translates into similar properties for interference (Figure S25‐S48). As such, we can recognize multiplicativity and additivity as two ‘valid’ models with different implications for the effect of mean fitness, and whichever implication best reflects biological examples in particular or in general is to be settled by empirical (not theoretical) research.

There is a large amount of consistent empirical evidence from experimental evolution that supports the decline in selection coefficients of beneficial mutations at different loci over the course of adaptation to a new yet constant environment in diverse bacteria (Chou et al., 2011; Flynn et al., 2013; Hall et al., 2019; Khan et al., 2011; Wang et al., 2016; Wünsche et al., 2017; Zee & Velicer, 2017), fungi (Kryazhimskiy et al., 2014; Kvitek & Sherlock, 2011; Schoustra et al., 2016; Wei & Zhang, 2019) and viruses (Bull et al., 1997; Holder & Bull, 2001; Sackman & Rokyta, 2018)—and this is also supported in the context of resistance evolution (MacLean et al., 2010; Schenk et al., 2013). Although this might seemingly support the additive fitness model, this prediction is also in‐keeping with Fisher's (1930) geometric model of adaptation, which predicts this outcome from a declining phenotypic effect size of beneficial mutations, which is not a consideration of additivity but does have some empirical support from QTL analysis (Bell, 2010; Dittmar et al., 2016; Orr, 2001; Remington & Purugganan, 2003; Roff, 2007; Tanksley, 1993). Also consistently across studies in experimental evolution that examine it, mutations are measured to have a larger selection coefficient if they occur earlier in a sequence of adaptive substitutions, leading to the widespread attribution of negative (or diminishing‐returns) epistasis in bacteria (Chou et al., 2011; Flynn et al., 2013; Hall et al., 2019; Khan et al., 2011; Wang et al., 2016; Wünsche et al., 2017; Zee & Velicer, 2017), fungi (Schoustra et al., 2016; Wei & Zhang, 2019) and in the context of resistance evolution (MacLean et al., 2010; Schenk et al., 2013). Moreover, in a few select studies, the *same* mutation is shown to have a larger selection coefficient if it occurs later in a sequence of adaptive substitutions (Chou et al., 2011; Flynn et al., 2013; Hall et al., 2019; Kryazhimskiy et al., 2014; Sackman & Rokyta, 2018; Wang et al., 2016; Wünsche et al., 2017; Zee & Velicer, 2017), which has been taken as support for epistasis acting globally to influence the selection coefficients of beneficial mutations irrespective of their phenotypic effects. This undermines the link between phenotypic effect sizes and selection coefficients that is implicit in Fisher's (1930) geometric model because a mutation's selection coefficient depends upon the baseline of mean fitness, which makes it useful to separate an intrinsic selection coefficient owing to its phenotype effects from an effective selective coefficient that may depend upon mean fitness. There is also suggestive evidence in support of additivity from the detection of hard sweeps by resistance mutations in natural populations (Barnes et al., 2017; Feder et al., 2016; Frenkel et al., 2015; Hawkins et al., 2019; Weedall et al., 2020), but there is debate around the genomic methods that are used to detect hard sweeps (Garud et al., 2015; Hernandez et al., 2011; Karasov et al., 2010; Redman et al., 2015). Therefore, empirical evidence (especially from experimental evolution) does widely support the fundamental feature of the addition (or nonmultiplication) of intrinsic selection coefficients in the shifting baseline of mean fitness that reduces the effective selection coefficient of mutations that occur later in a sequence of adaptive substitutions.

The implication of the empirical support for additivity from experimental evolution is that the neglect of genetic drift in resistance models ignores an important aspect of resistance evolution as it occurs in real biological systems. Consequently, formulating a resistance‐management plan in a way that ignores interference may undervalue the effects of strategies like mixtures that generate simultaneous selection pressures (in contrast to alternative strategies like the solo‐use of pesticides sequentially). However, the extent of the undervaluation remains to be quantified in a more explicit model of resistance evolution and to be tested in an experimental set‐up that measures the probability of and time to resistance. The wider significance of this empirical evidence must be treated with caution because studies of experimental evolution in laboratory populations, like studies of resistance evolution in natural populations, tend to have strong selection. Yet, it seems that there are two qualitatively different models for the genetic dynamics of adaptation that hinge upon the interference between beneficial mutations at different loci, which are not necessarily incompatible when viewed as having different domains of application: multiplicativity fits with the view that complex adaptations in natural populations evolve gradually through the simultaneous spread of a large number of beneficial mutations with small effects that affect independent traits (Bell, 2010; Dobzhansky, 1937; Fisher, 1930; Fisher & Ford, 1947; Lande, 1983; Orr, 1998, 1999; Orr & Coyne, 1992), additivity fits with the view that humans often drive rapid adaptation in pest, pathogen and domesticated populations leading to the sequential spread of beneficial mutations with large effects (Endler, 1986; Haldane, 1957; Hazel & Lush, 1942; Kimura, 1983; Neher, 2013; Robertson, 1961; Wright, 1931, 1977). The key to which model is appropriate is not intervention by humans, as, for example, unconscious selection for domestication may be more gradual, but rather the rate at which an environment changes its selection pressures, like with the sudden application of a pesticide. The resulting implications for the genetics of adaptation are summarized in Table 2. In general, the predictions of multiplicativity would be a widely accepted null hypothesis for the genetics of adaptation consistent with Fisher's (1930) geometric model that predicts a declining rate of adaptation from the simultaneous substitution of beneficial mutations with smaller phenotypic effect sizes (that are more likely to bring a phenotype closer to its optimum) and correspondingly smaller intrinsic selection coefficients. In this way, the null hypothesis of multiplicativity suggests that it is as if evolution acts on populations by prioritizing the sweeping of beneficial mutations to fixation in order of their intrinsic selection coefficient. In contrast, not without precedent (e.g. Wright, 1931; Haldane, 1957) and akin to the inefficient ‘tandem method’ of artificial selection (Hazel & Lush, 1942), the substantial interference under additivity presents an alternate hypothesis for the genetics of adaptations that predicts a declining rate of adaptation from the sequential substitution of beneficial mutations with larger effective selection coefficients due to the shifting baseline of mean fitness. In this way, the alternative hypothesis of additivity suggests that it is as if evolution—through the combined effects of selection and drift in generating substantial interference—acts on populations by prioritizing beneficial mutations in order of their frequency (and/or the timing of mutation). Accordingly, the null and alternative hypotheses are distinguished in contrasting evolutionary trajectories for a new beneficial mutation with a larger intrinsic selection coefficient arises during an ongoing substitution for a beneficial allele with a smaller intrinsic selection coefficient. Whilst clearly distinctive, these hypotheses for the effects of interference for multiplicativity and additivity have yet to be experimentally tested. An ideal experimental set‐up to test fitness models would be to replicate the conditions of the simulations that are run here, using two pesticides that target a sexual pest with well‐characterized resistance mutations isolated within different resistant strains, which would enable starting populations to be assembled from different frequencies of those strains. The test would involve the regular sampling of an evolving population to observe the change in allele frequencies and estimate the selection coefficients for resistance mutations with exposure to one or other or both pesticides.

**TABLE 2:** Differences in the predictions of interlocus interference under multiplicativity and additivity for strongly selected alleles, like resistance mutations

	Multiplicativity $\omega_{\mathrm{AB}}=\left(1+s_{A}\right)\;\left(1+s_{B}\right)$	Additivity $\omega_{\mathrm{AB}}=1+s_{A}+s_{B}$
Probability of fixation	Unchanged, or slight decrease	Substantial decrease
Average time to fixation	Unchanged	Substantial increase
Rate of adaptation	Declining, due to declining phenotypic effect size	Declining, due to the shifting baseline of mean fitness
Pattern of allele fixation	Simultaneous	Sequential
Order of mutation fixation	Intrinsic selection coefficient (large to small)^a^	Frequency (large to small) or timing of mutation (not overlapping with ongoing substitution)^b^

^a^Under multiplicativity, the effective and intrinsic selection coefficients are so near‐equal as to be equivalent, which is emphatically not the case under additivity. The timing of mutation may also be important when adaptation is mutation limited, but this is shared with additivity, where the timing of mutation has additional importance due to the sequential pattern of allele fixation.

^b^There is also a trend in the size (large to small) of effective selection coefficient if beneficial mutations have the same starting frequency.

### PEER REVIEW

The peer review history for this article is available at https://publons.com/publon/10.1111/jeb.13919.

### OPEN RESEARCH BADGES

This article has earned an Open Data Badge for making publicly available the digitally‐shareable data necessary to reproduce the reported results. The data is available at [https://doi.org/10.5281/zenodo.5226387].

## Supporting information

Appendix S1

## Data Availability

Data available in article supplementary material and https://doi.org/10.5281/zenodo.5226387
