# Supplementary figures and images for: Evolution of resistance under alternative models of selective interference
Source: J Evol Biol. 2021 Sep 25;34(10):1608–23. doi: 10.1111/jeb.13919 (PMC9293239; doi:10.1111/jeb.13919)

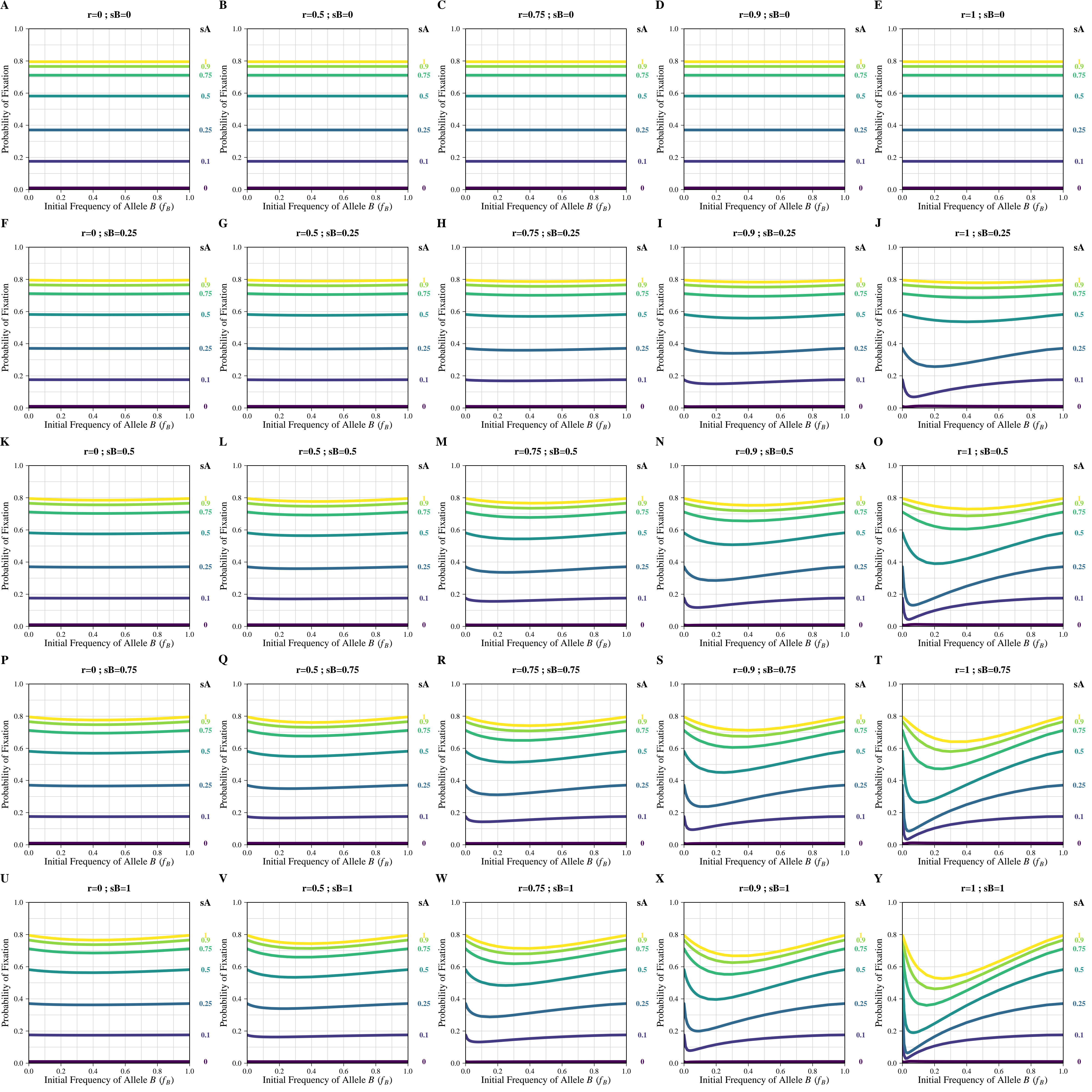

Supplement: Supplementary file 1 — Appendix S1 [file JEB-34-1608-s001.zip › SupportingInformation/FigureS01_PFIXA_stochastic_multiplicative_t1000_n100.pdf]

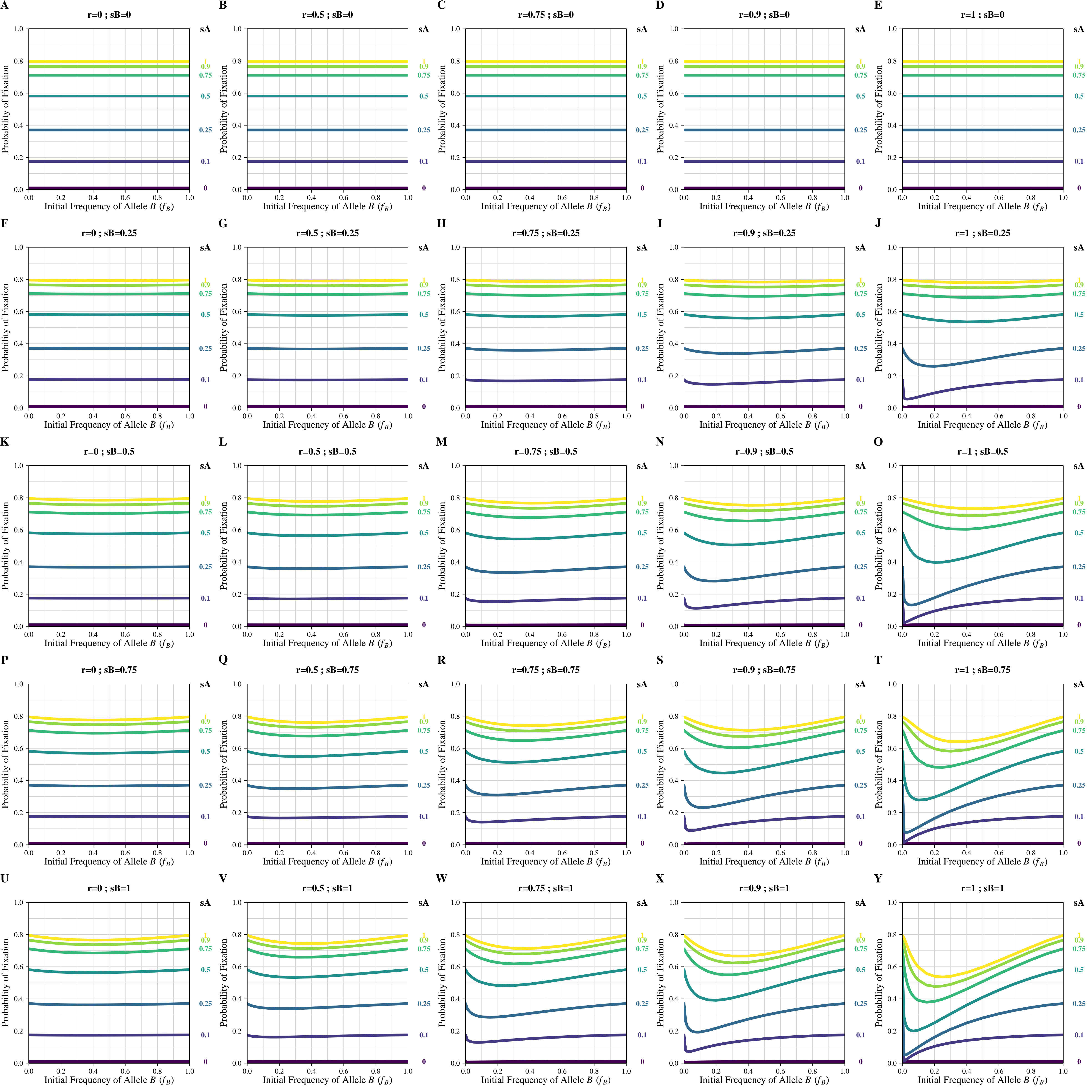

Supplement: Supplementary file 1 — Appendix S1 [file JEB-34-1608-s001.zip › SupportingInformation/FigureS02_PFIXA_deterministic_multiplicative_t1000_n100.pdf]

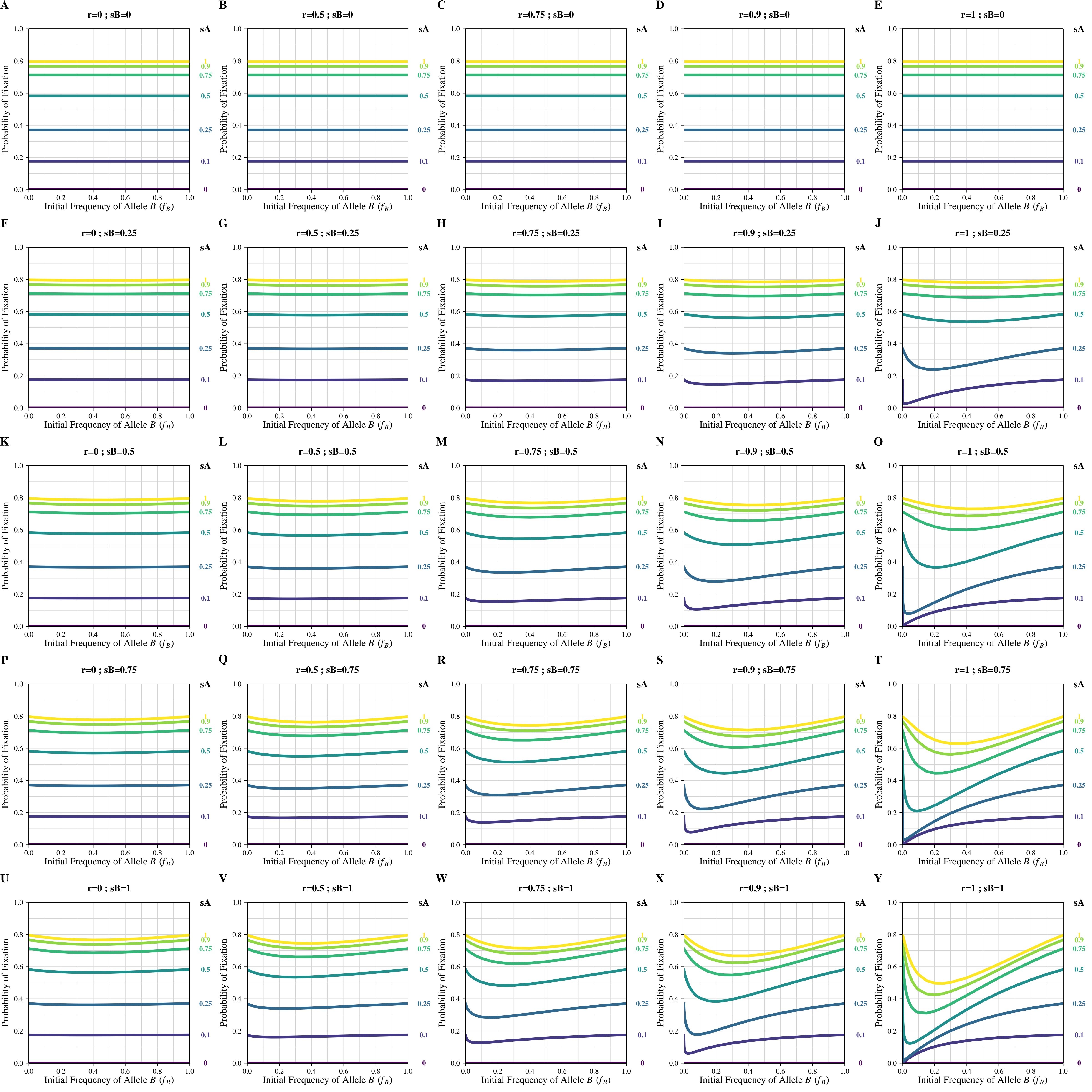

Supplement: Supplementary file 1 — Appendix S1 [file JEB-34-1608-s001.zip › SupportingInformation/FigureS03_PFIXA_deterministic_multiplicative_t10000_n1000.pdf]

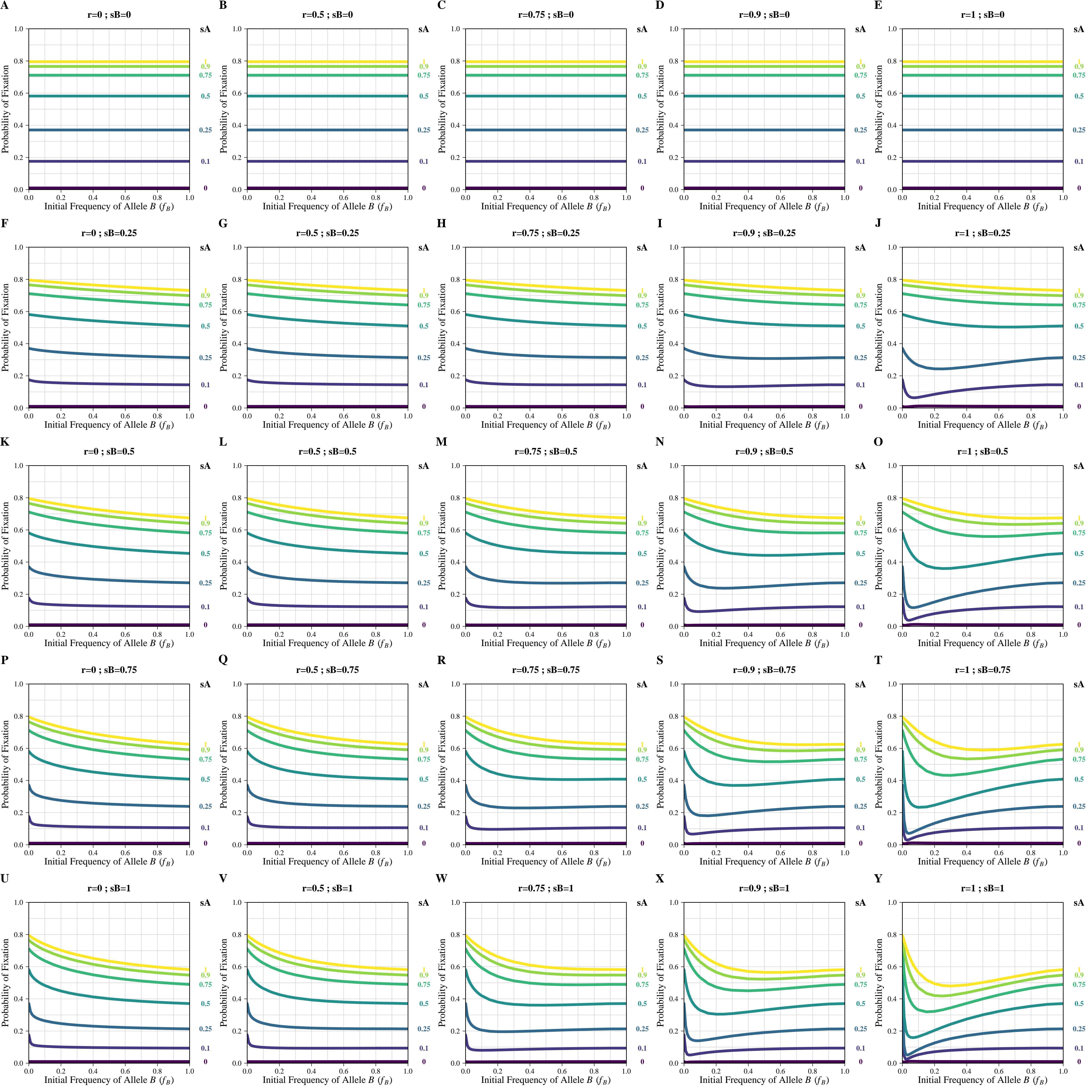

Supplement: Supplementary file 1 — Appendix S1 [file JEB-34-1608-s001.zip › SupportingInformation/FigureS04_PFIXA_stochastic_additive_t1000_n100.pdf]

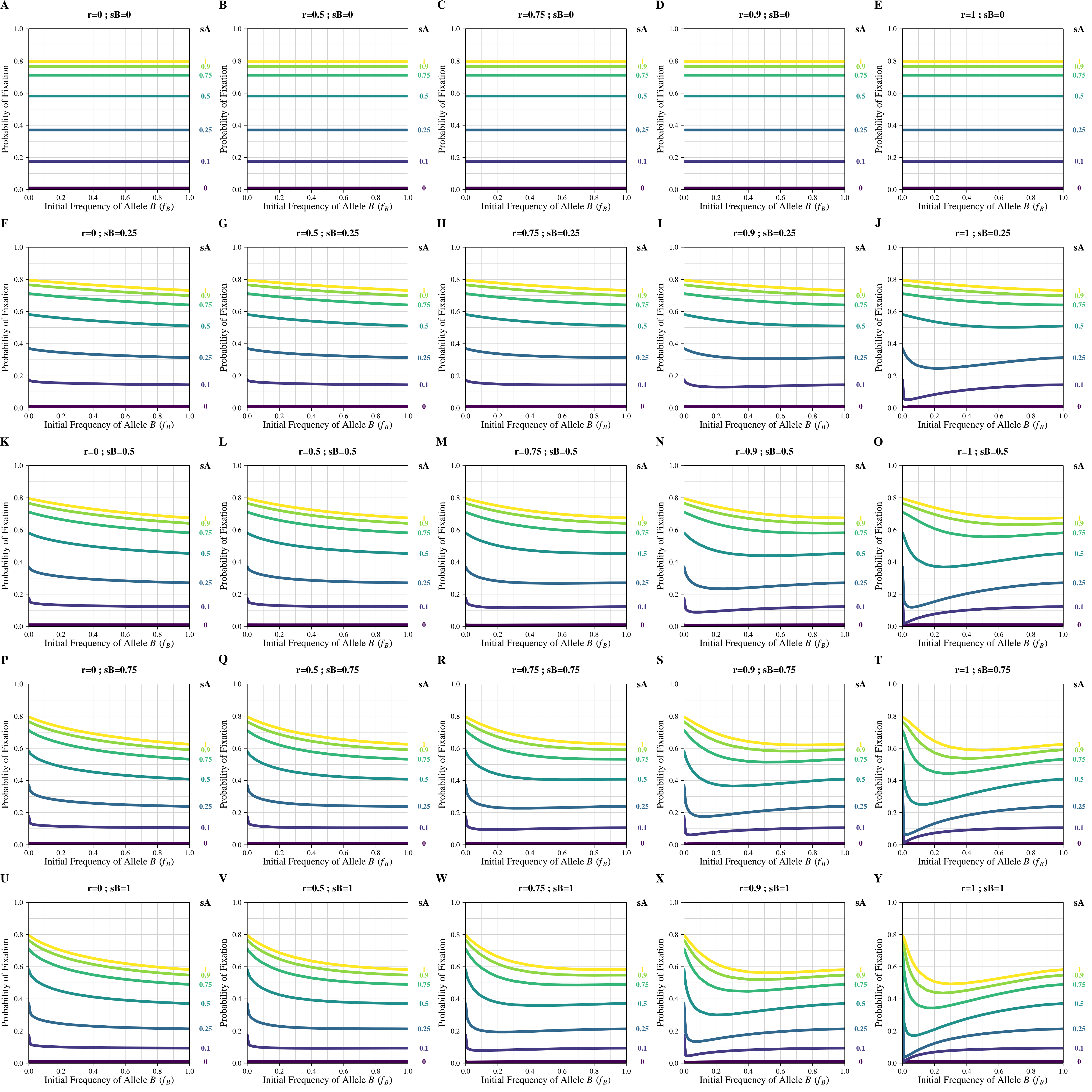

Supplement: Supplementary file 1 — Appendix S1 [file JEB-34-1608-s001.zip › SupportingInformation/FigureS05_PFIXA_deterministic_additive_t1000_n100.pdf]

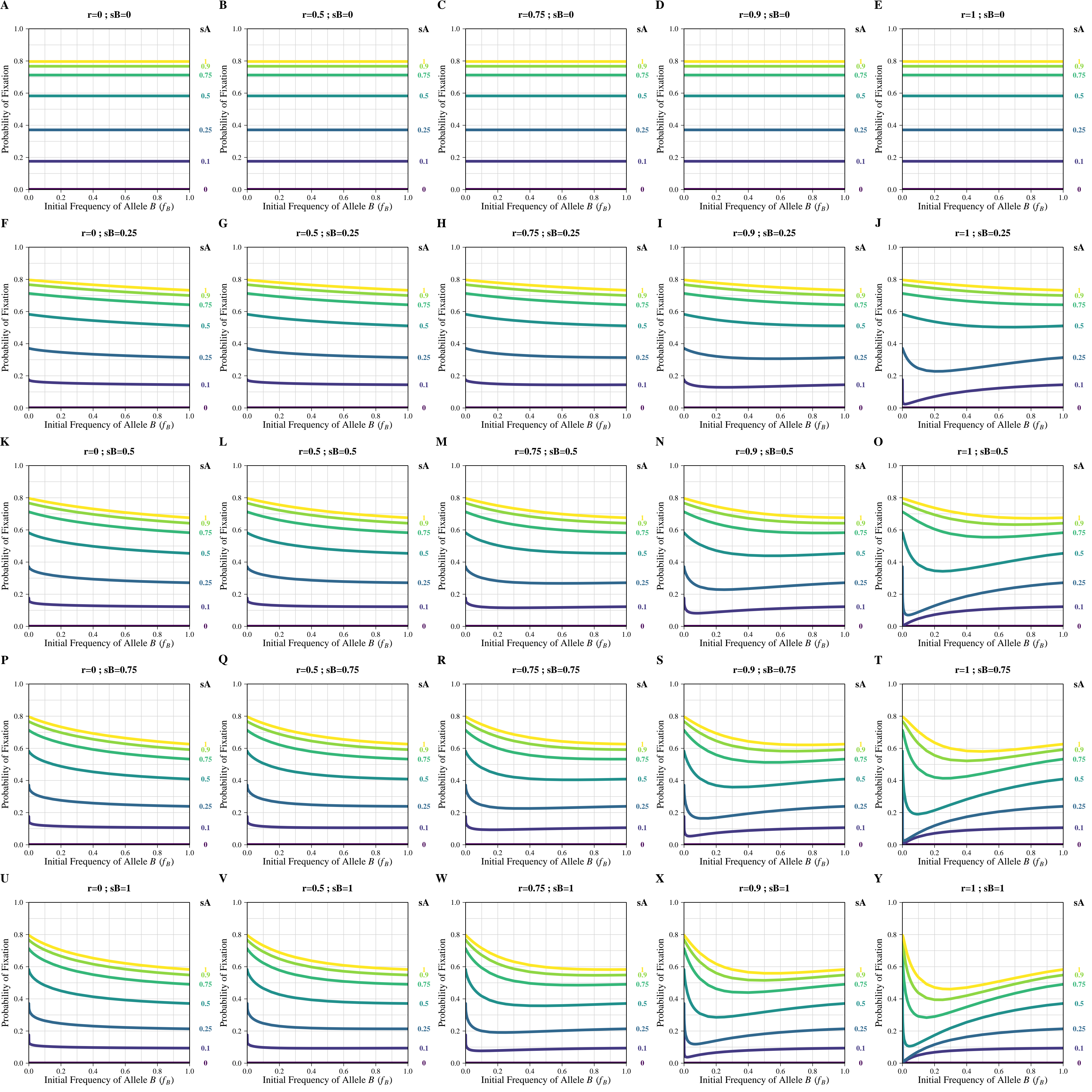

Supplement: Supplementary file 1 — Appendix S1 [file JEB-34-1608-s001.zip › SupportingInformation/FigureS06_PFIXA_deterministic_additive_t10000_n1000.pdf]

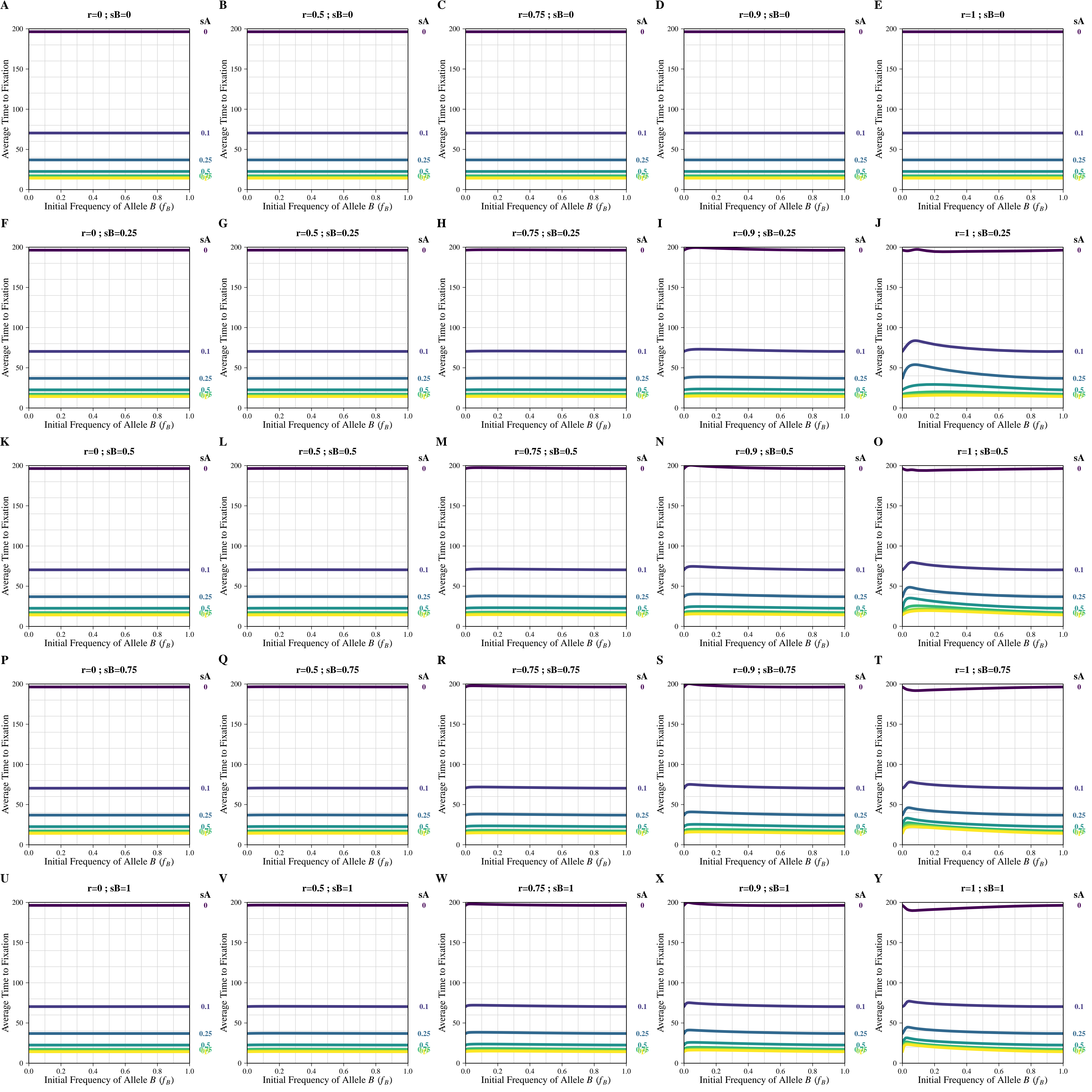

Supplement: Supplementary file 1 — Appendix S1 [file JEB-34-1608-s001.zip › SupportingInformation/FigureS07_TFIXA_stochastic_multiplicative_t1000_n100.pdf]

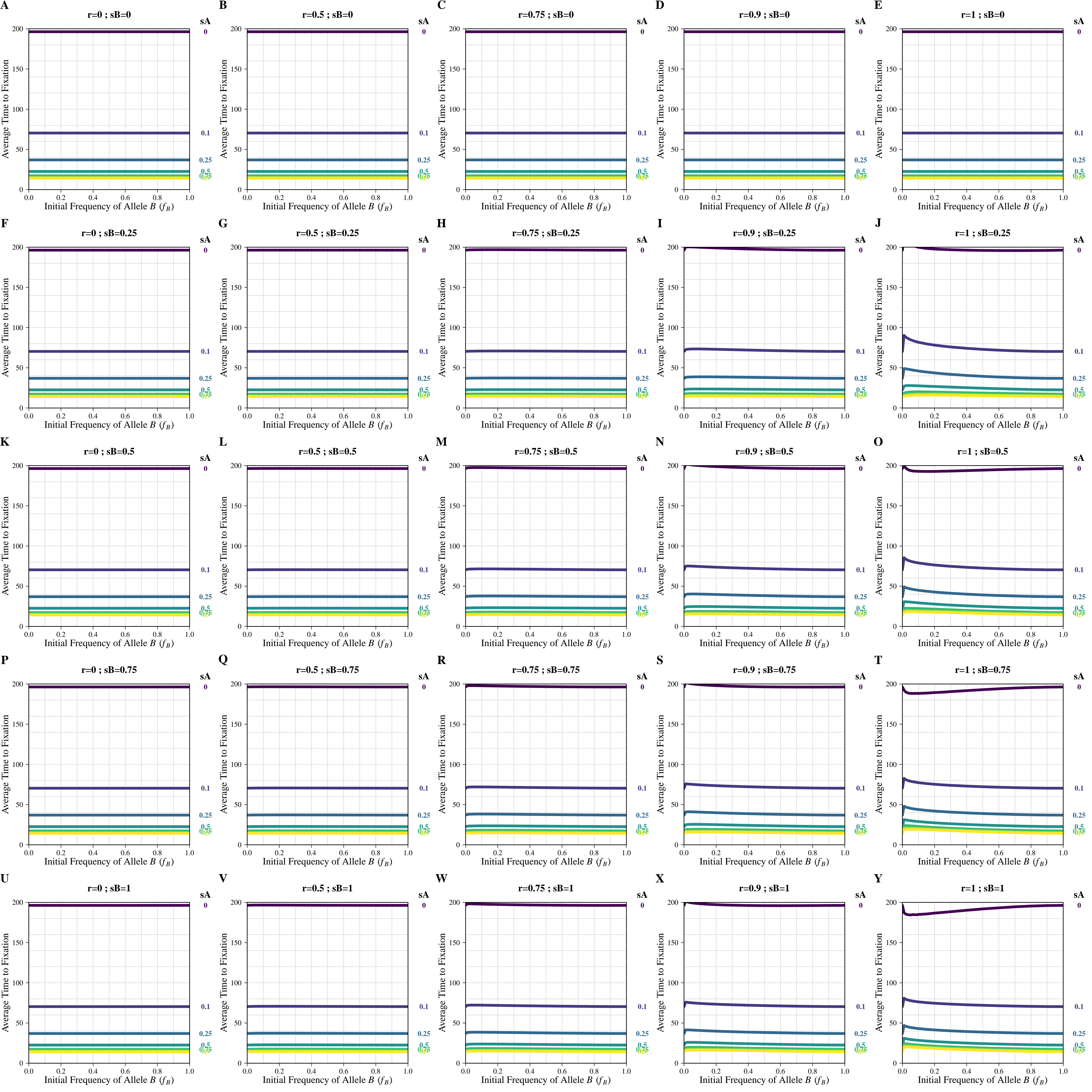

Supplement: Supplementary file 1 — Appendix S1 [file JEB-34-1608-s001.zip › SupportingInformation/FigureS08_TFIXA_deterministic_multiplicative_t1000_n100.pdf]

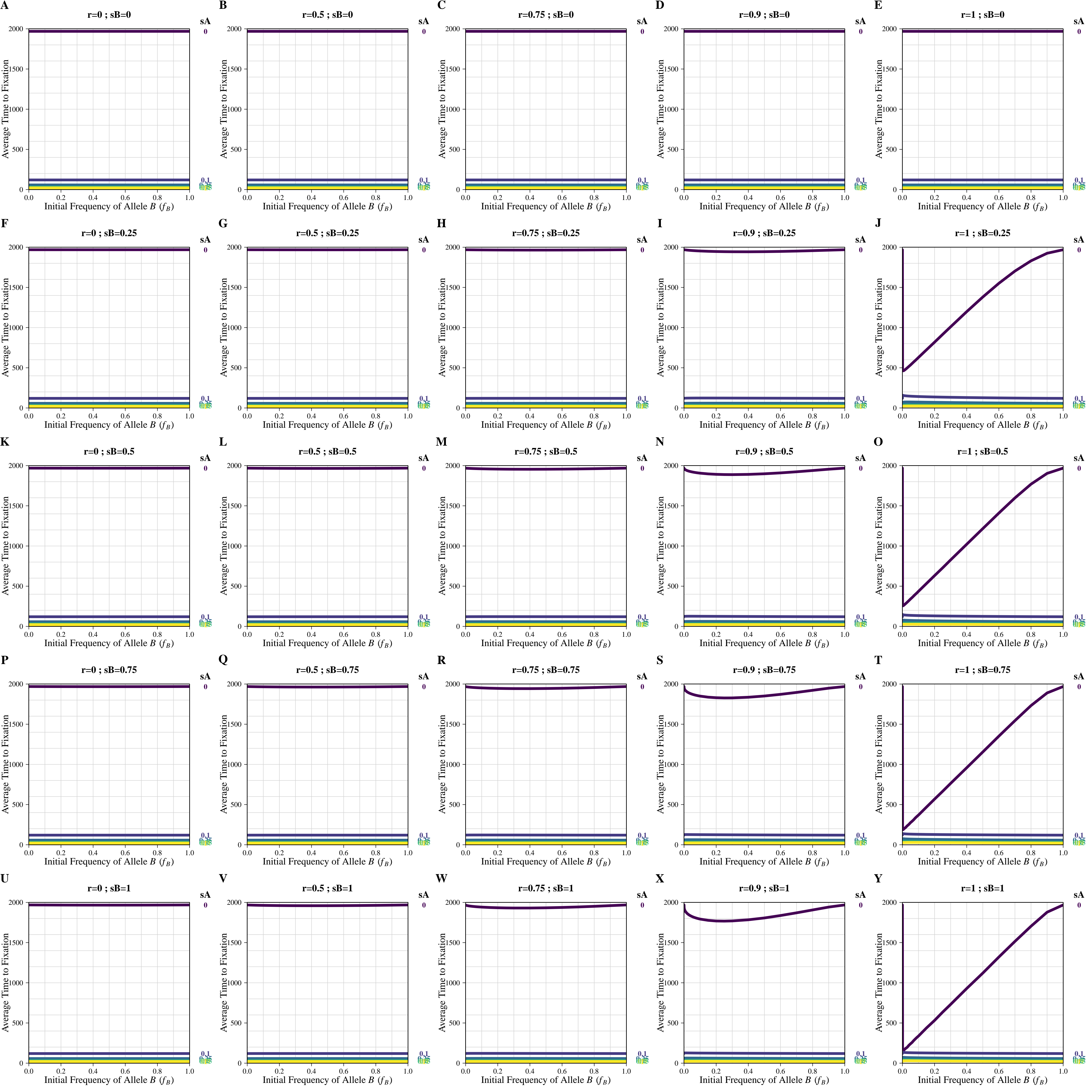

Supplement: Supplementary file 1 — Appendix S1 [file JEB-34-1608-s001.zip › SupportingInformation/FigureS09_TFIXA_deterministic_multiplicative_t10000_n1000.pdf]

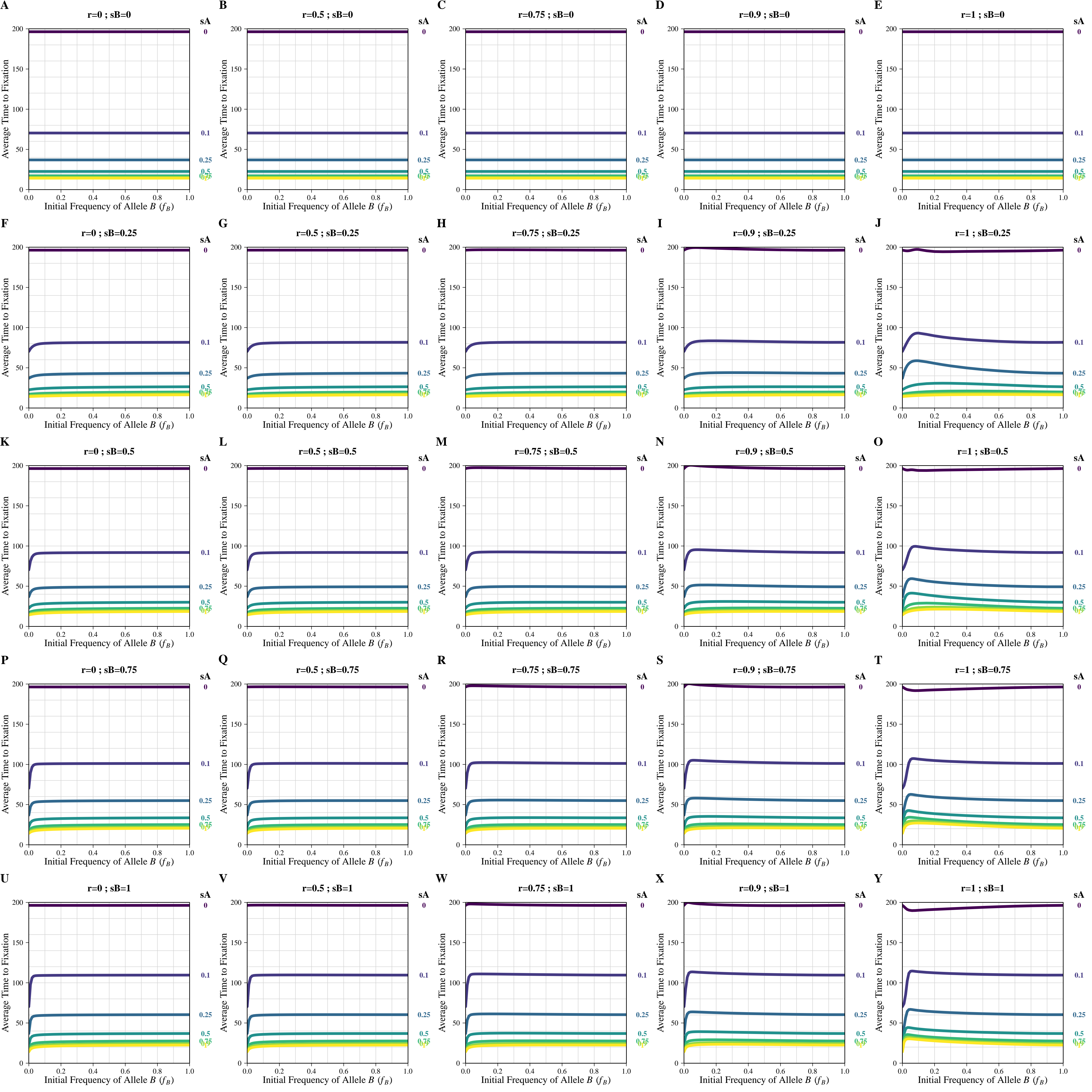

Supplement: Supplementary file 1 — Appendix S1 [file JEB-34-1608-s001.zip › SupportingInformation/FigureS10_TFIXA_stochastic_additive_t1000_n100.pdf]

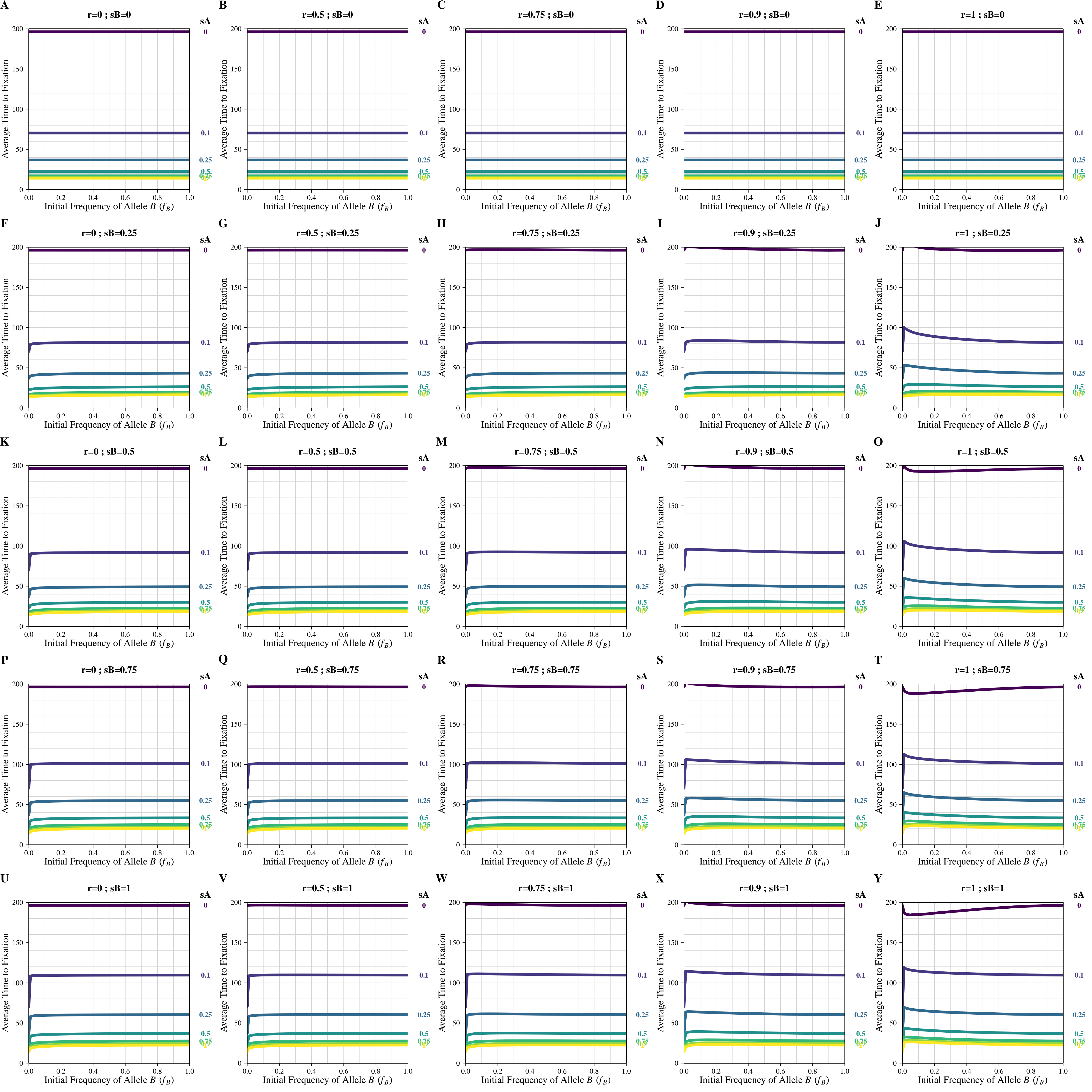

Supplement: Supplementary file 1 — Appendix S1 [file JEB-34-1608-s001.zip › SupportingInformation/FigureS11_TFIXA_deterministic_additive_t1000_n100.pdf]

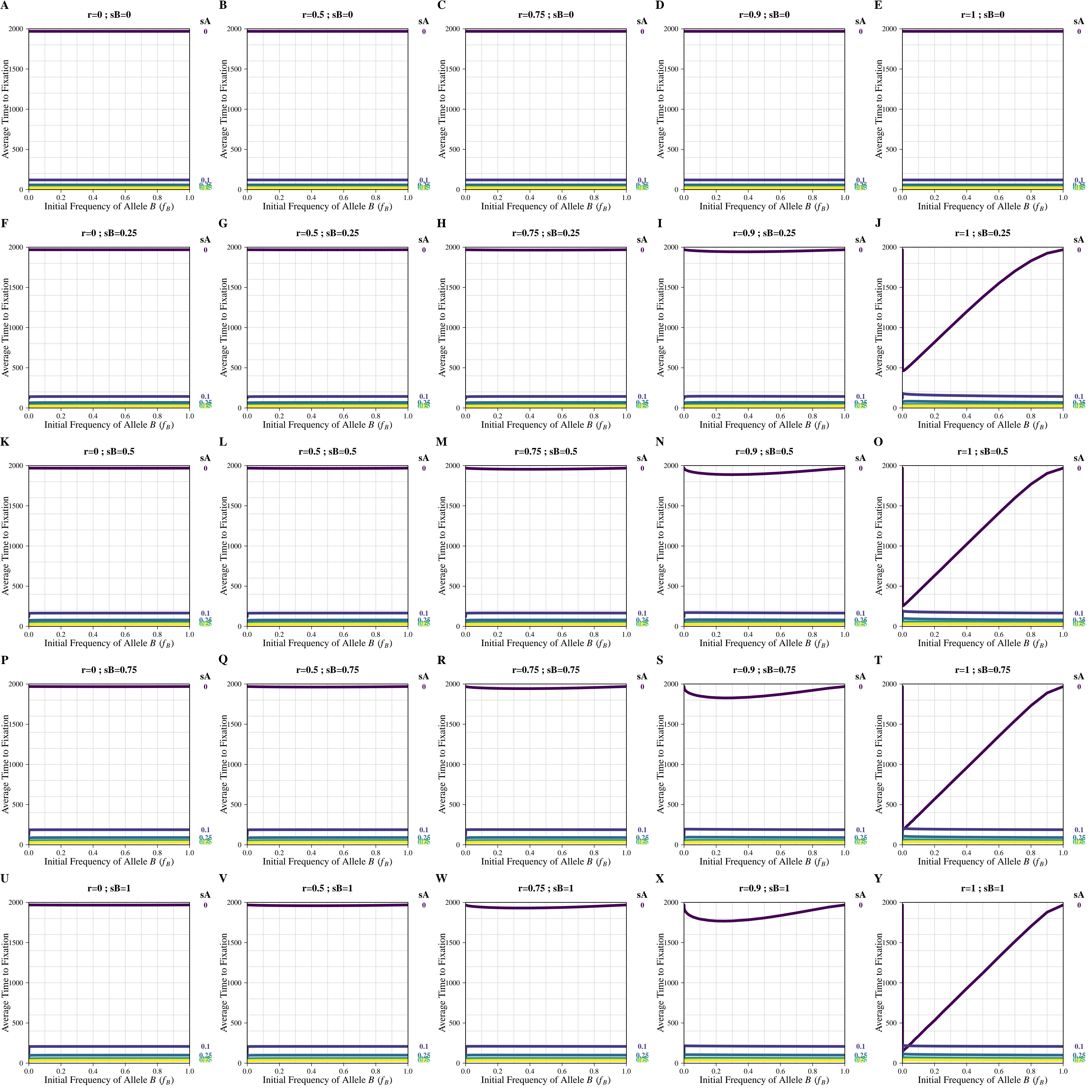

Supplement: Supplementary file 1 — Appendix S1 [file JEB-34-1608-s001.zip › SupportingInformation/FigureS12_TFIXA_deterministic_additive_t10000_n1000.pdf]

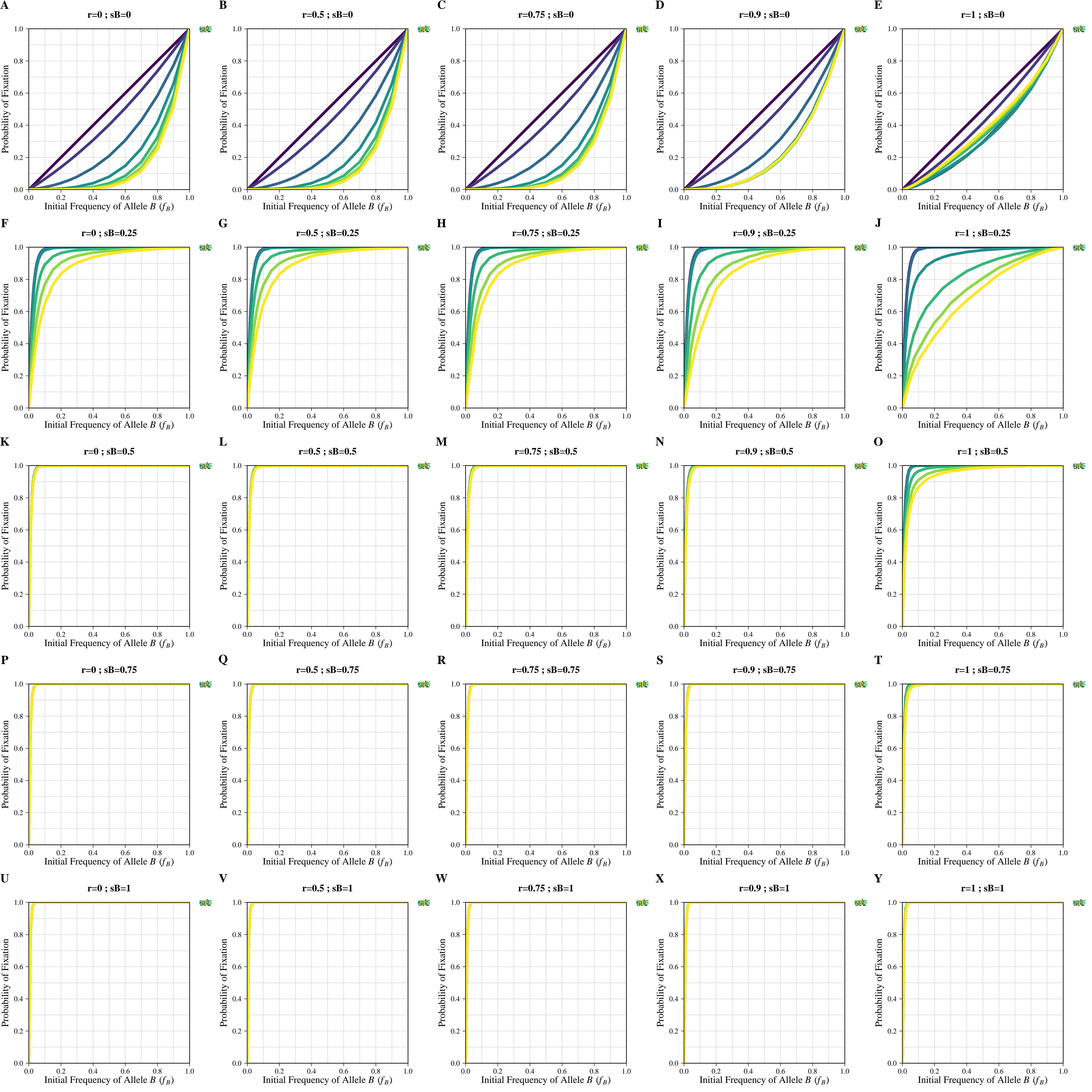

Supplement: Supplementary file 1 — Appendix S1 [file JEB-34-1608-s001.zip › SupportingInformation/FigureS13_PFIXB_stochastic_multiplicative_t1000_n100.pdf]

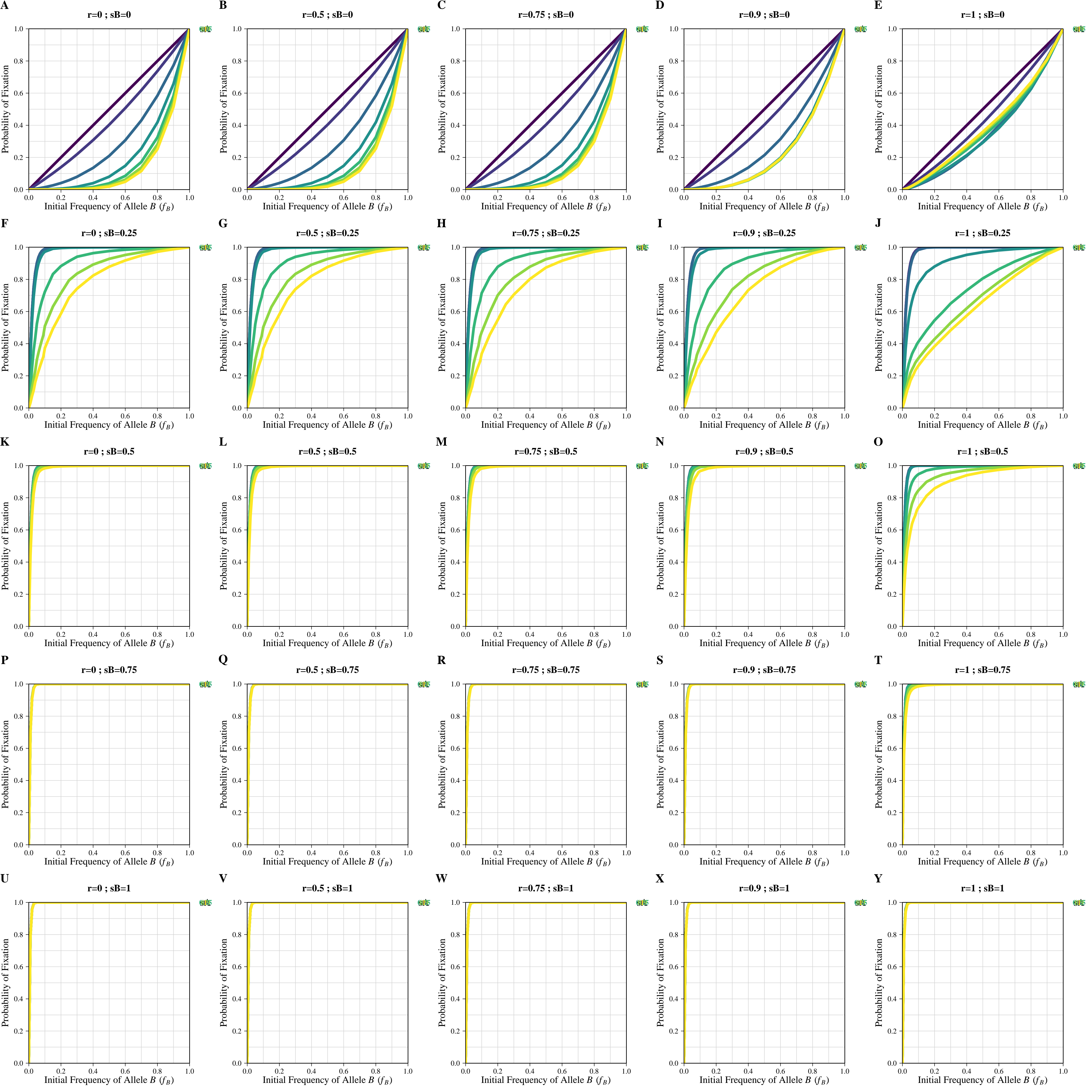

Supplement: Supplementary file 1 — Appendix S1 [file JEB-34-1608-s001.zip › SupportingInformation/FigureS14_PFIXB_stochastic_additive_t1000_n100.pdf]

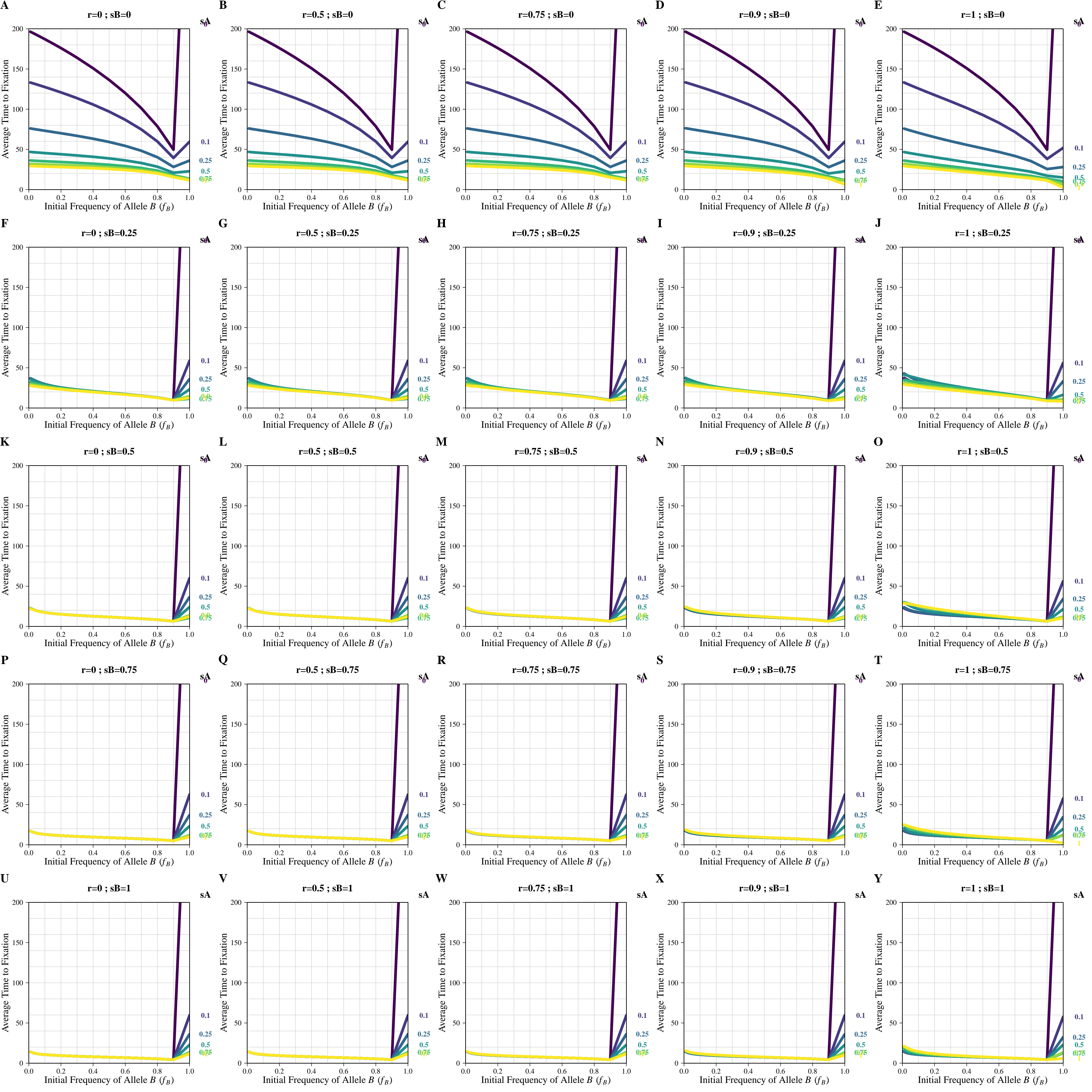

Supplement: Supplementary file 1 — Appendix S1 [file JEB-34-1608-s001.zip › SupportingInformation/FigureS15_TFIXB_stochastic_multiplicative_t1000_n100.pdf]

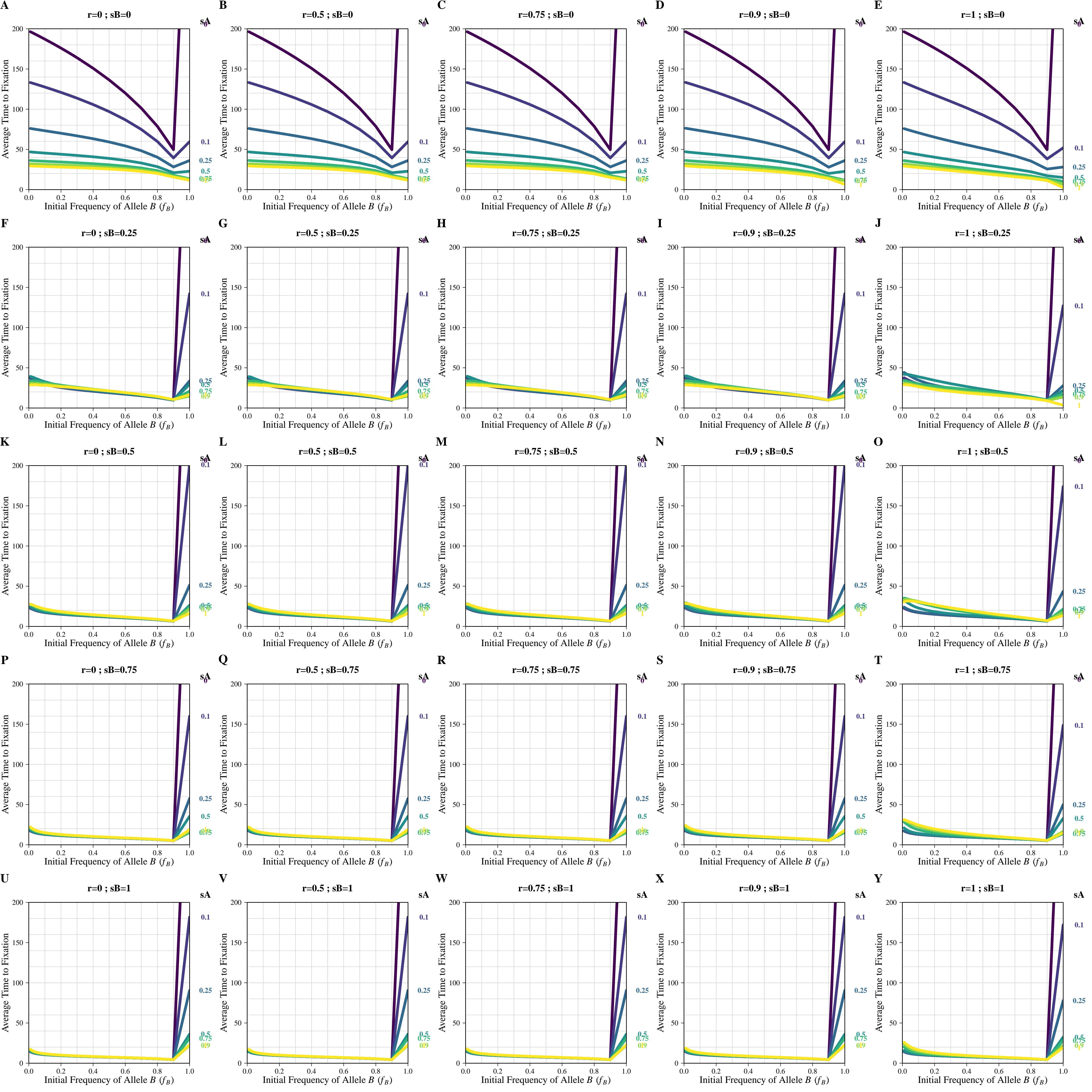

Supplement: Supplementary file 1 — Appendix S1 [file JEB-34-1608-s001.zip › SupportingInformation/FigureS16_TFIXB_stochastic_additive_t1000_n100.pdf]

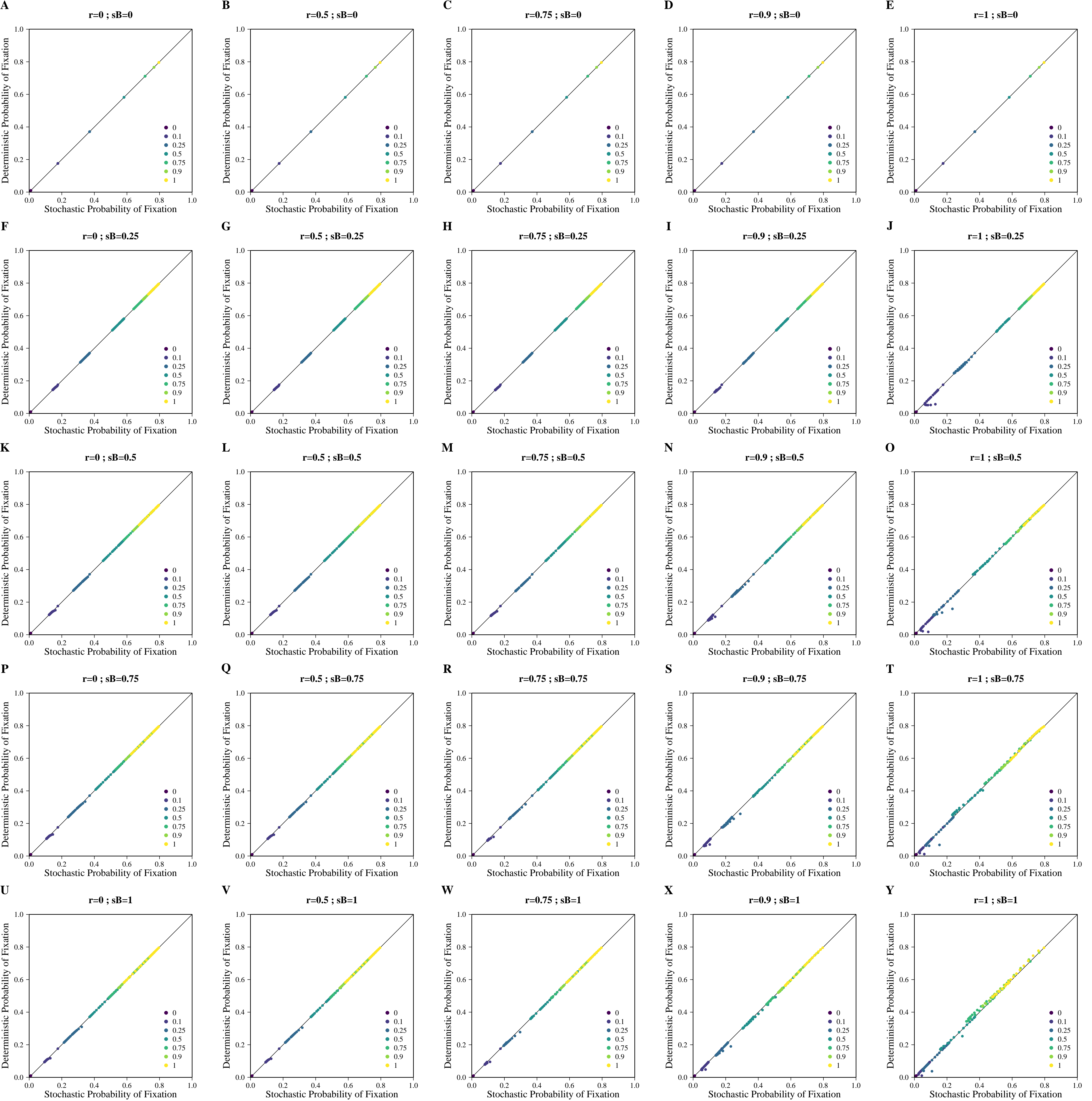

Supplement: Supplementary file 1 — Appendix S1 [file JEB-34-1608-s001.zip › SupportingInformation/FigureS17_COMPARISON_PFIXA_STOvsDET_additive_t1000_n100.pdf]

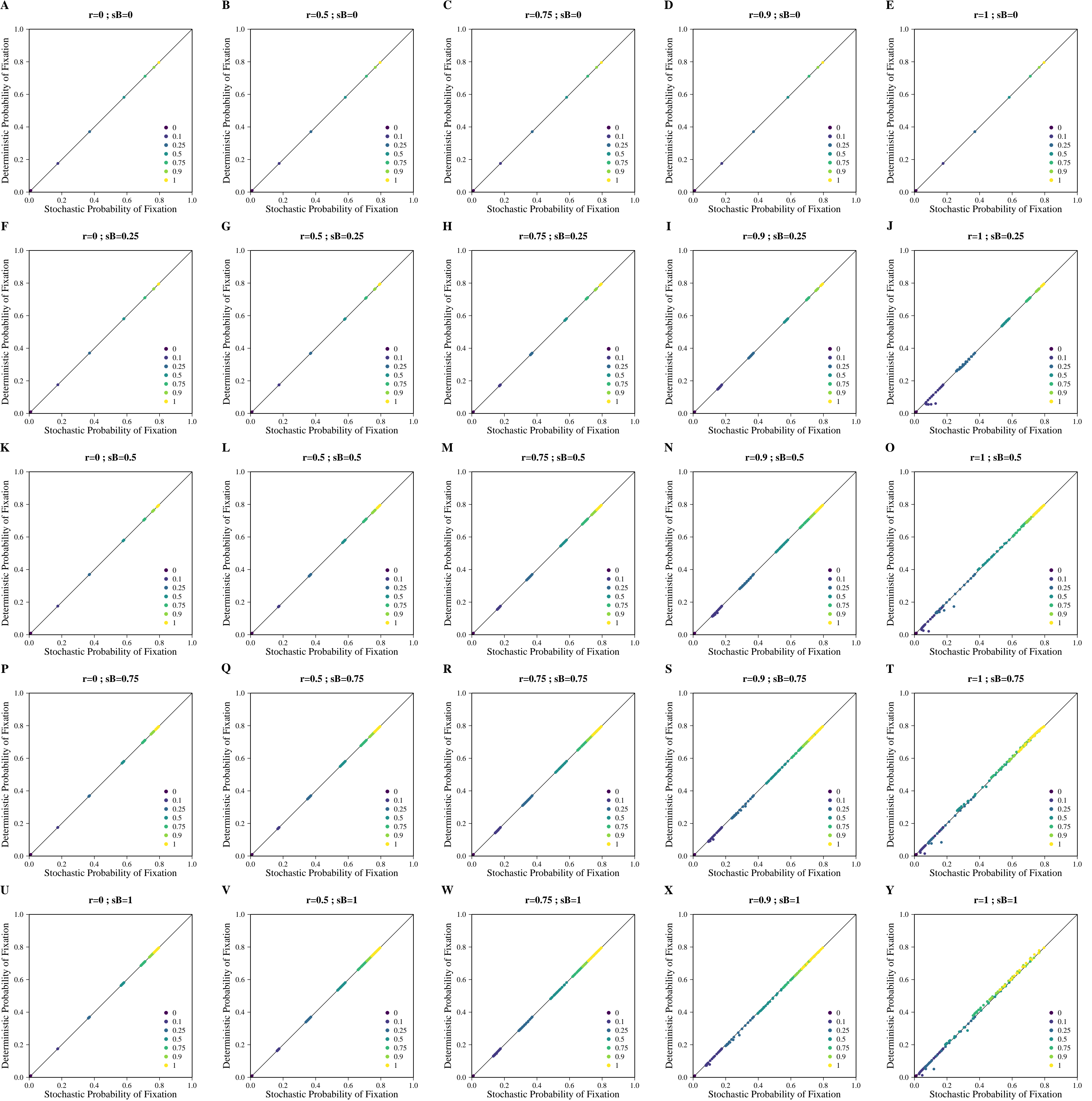

Supplement: Supplementary file 1 — Appendix S1 [file JEB-34-1608-s001.zip › SupportingInformation/FigureS18_COMPARISON_PFIXA_STOvsDET_multiplicative_t1000_n100.pdf]

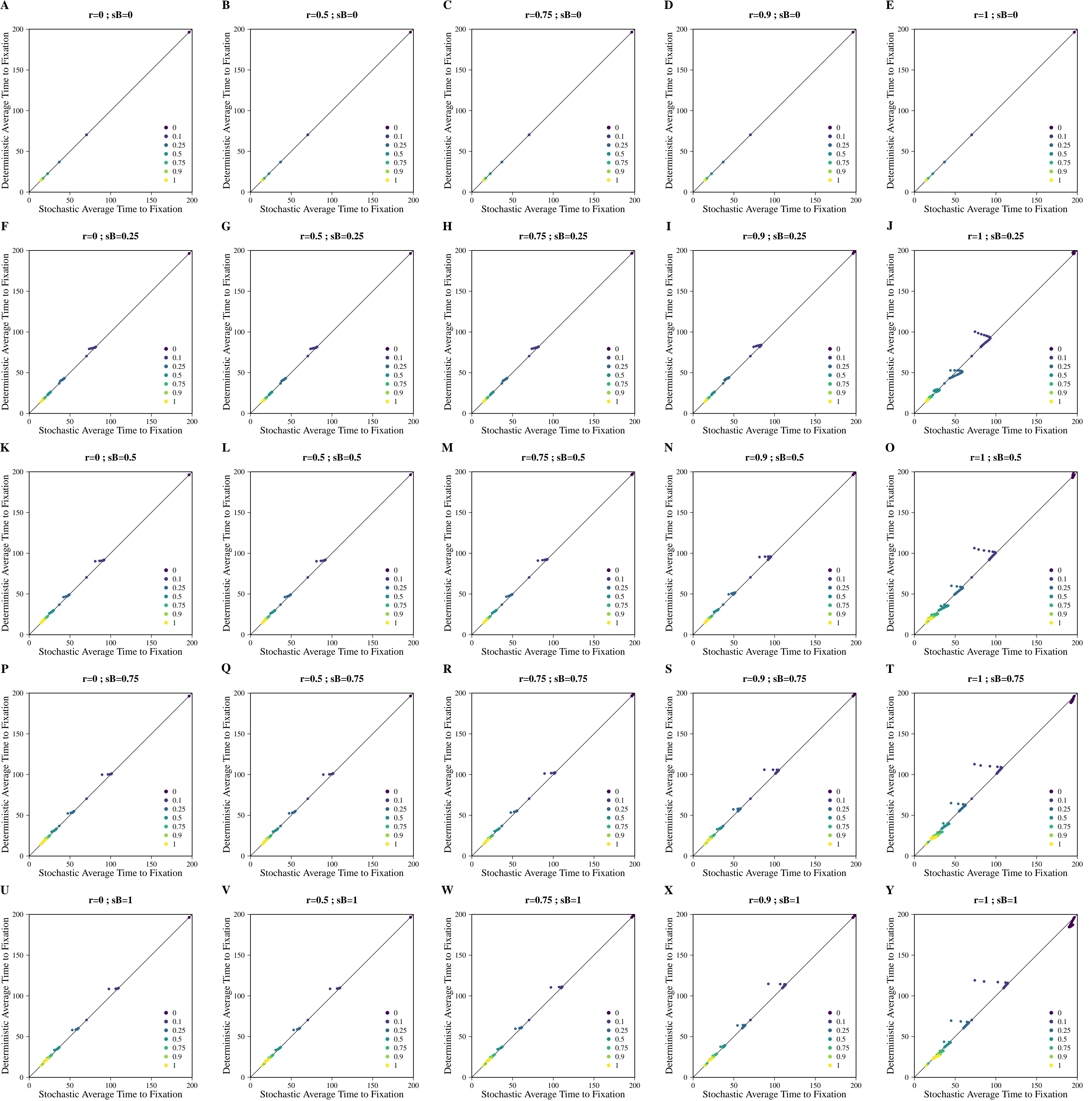

Supplement: Supplementary file 1 — Appendix S1 [file JEB-34-1608-s001.zip › SupportingInformation/FigureS19_COMPARISON_TFIXA_STOvsDET_additive_t1000_n100.pdf]

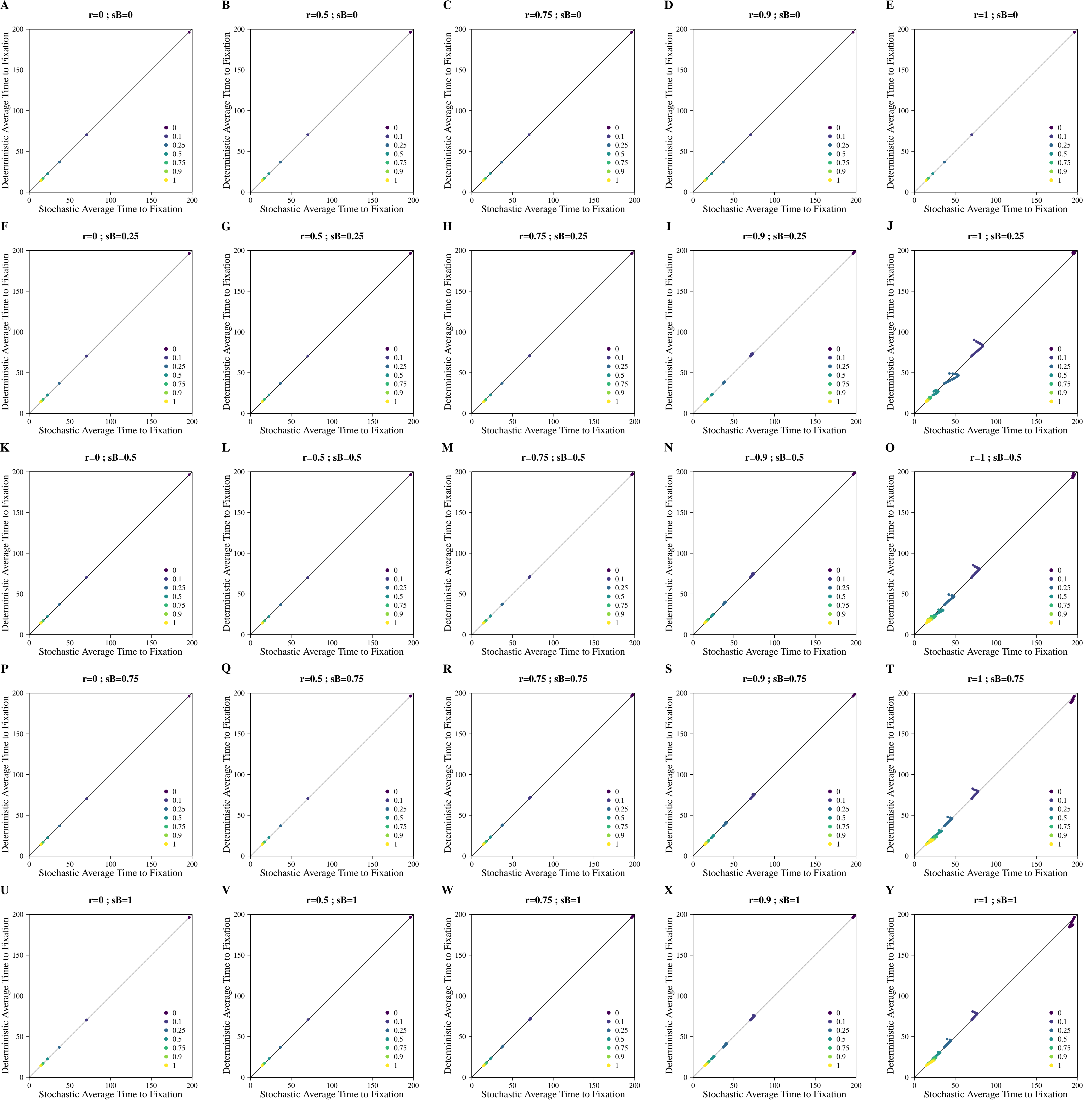

Supplement: Supplementary file 1 — Appendix S1 [file JEB-34-1608-s001.zip › SupportingInformation/FigureS20_COMPARISON_TFIXA_STOvsDET_multiplicative_t1000_n100.pdf]

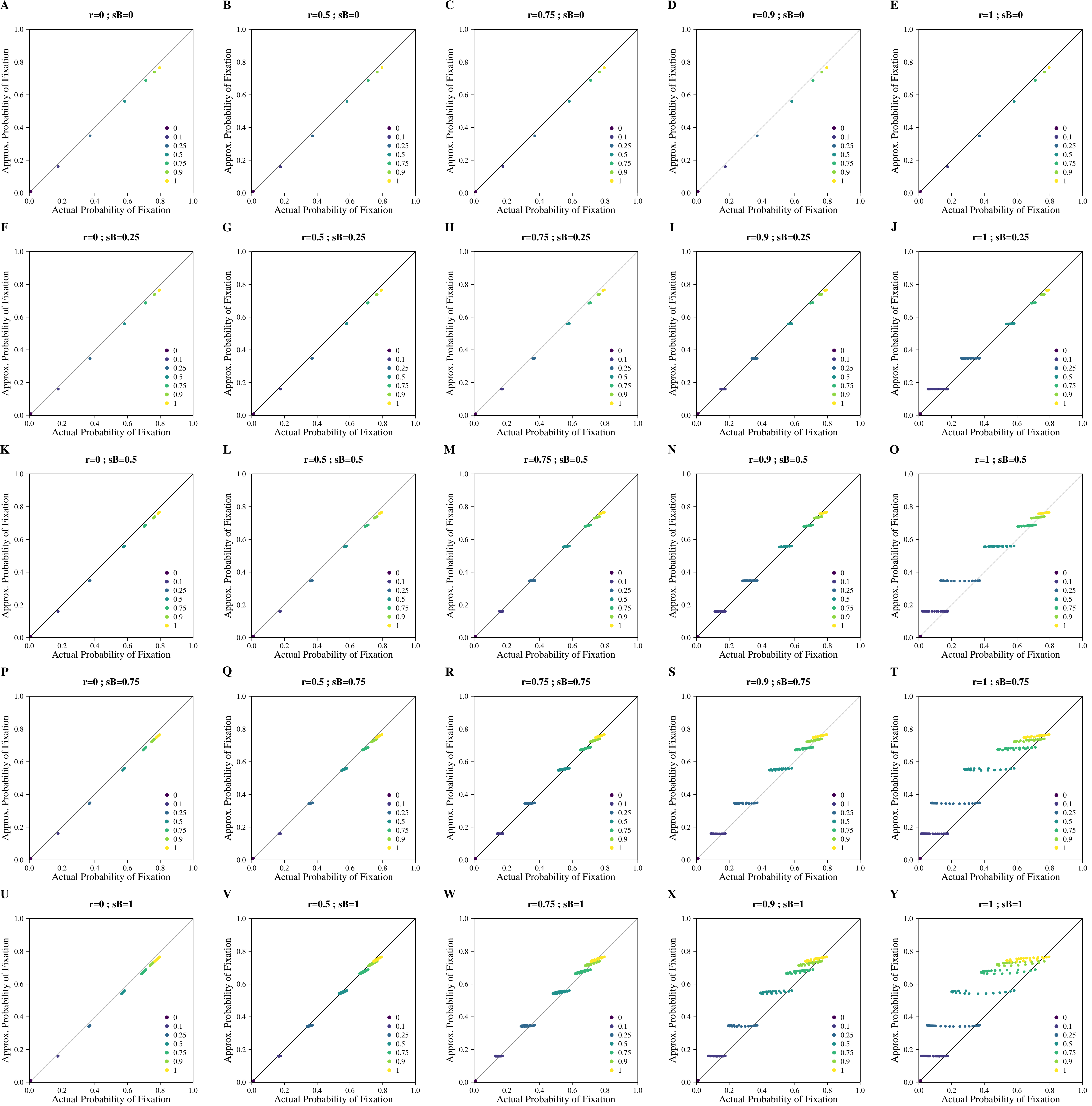

Supplement: Supplementary file 1 — Appendix S1 [file JEB-34-1608-s001.zip › SupportingInformation/FigureS21_COMPARISON_PFIXA_ACTvsAPP_multiplicative_t1000_n100.pdf]

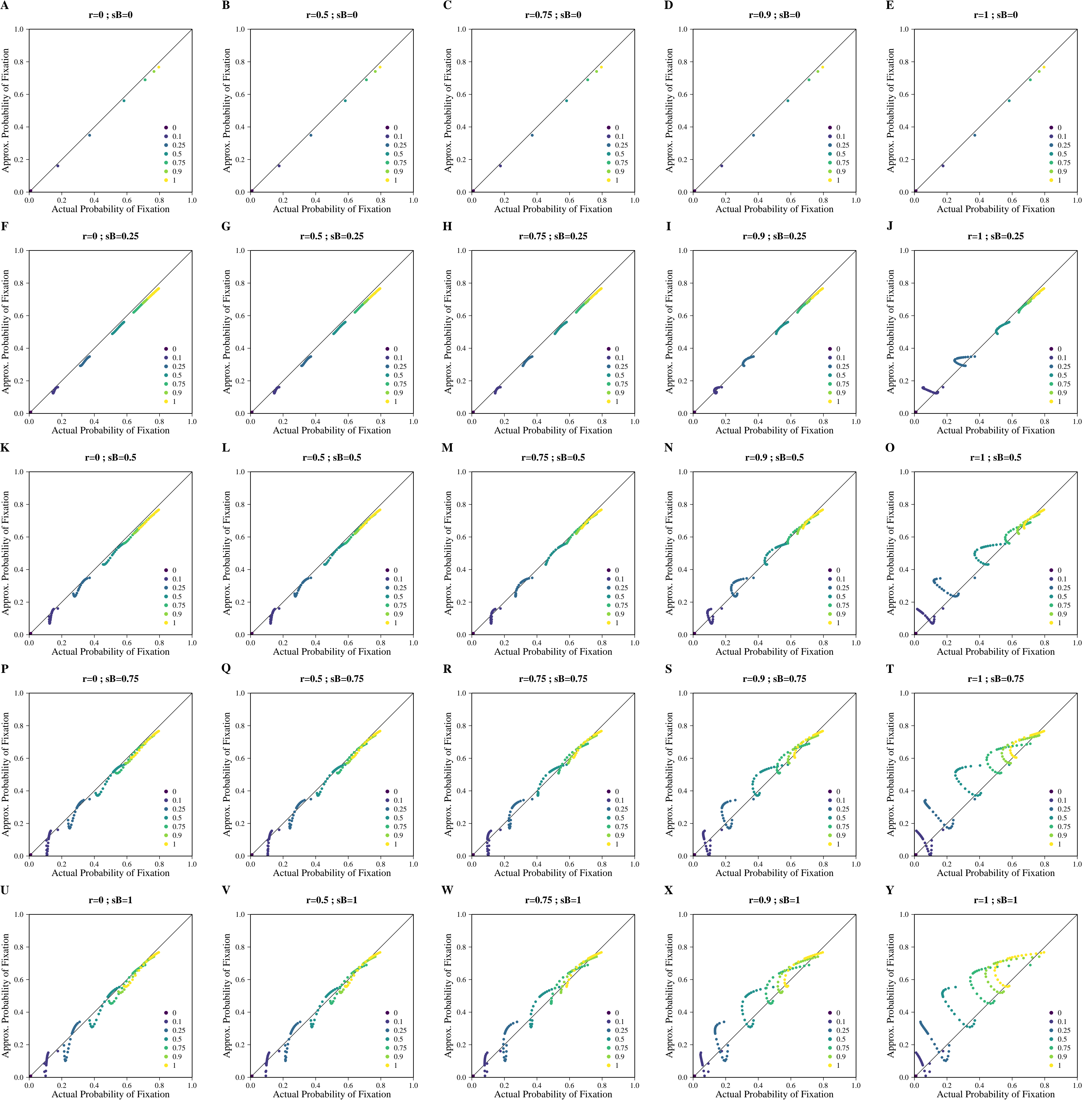

Supplement: Supplementary file 1 — Appendix S1 [file JEB-34-1608-s001.zip › SupportingInformation/FigureS22_COMPARISON_PFIXA_ACTvsAPP_additive_t1000_n100.pdf]

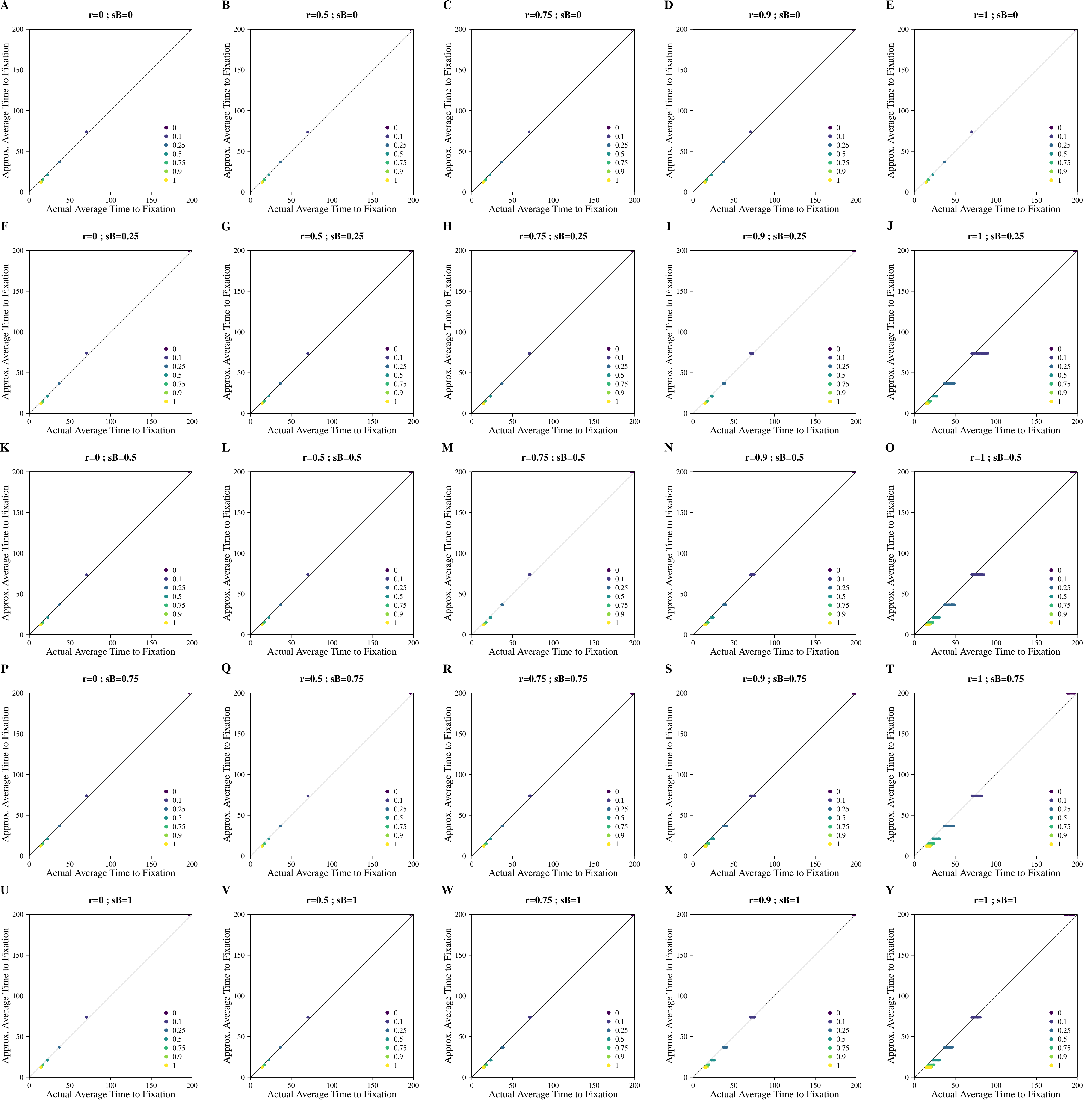

Supplement: Supplementary file 1 — Appendix S1 [file JEB-34-1608-s001.zip › SupportingInformation/FigureS23_COMPARISON_TFIXA_ACTvsAPP_multiplicative_t1000_n100.pdf]

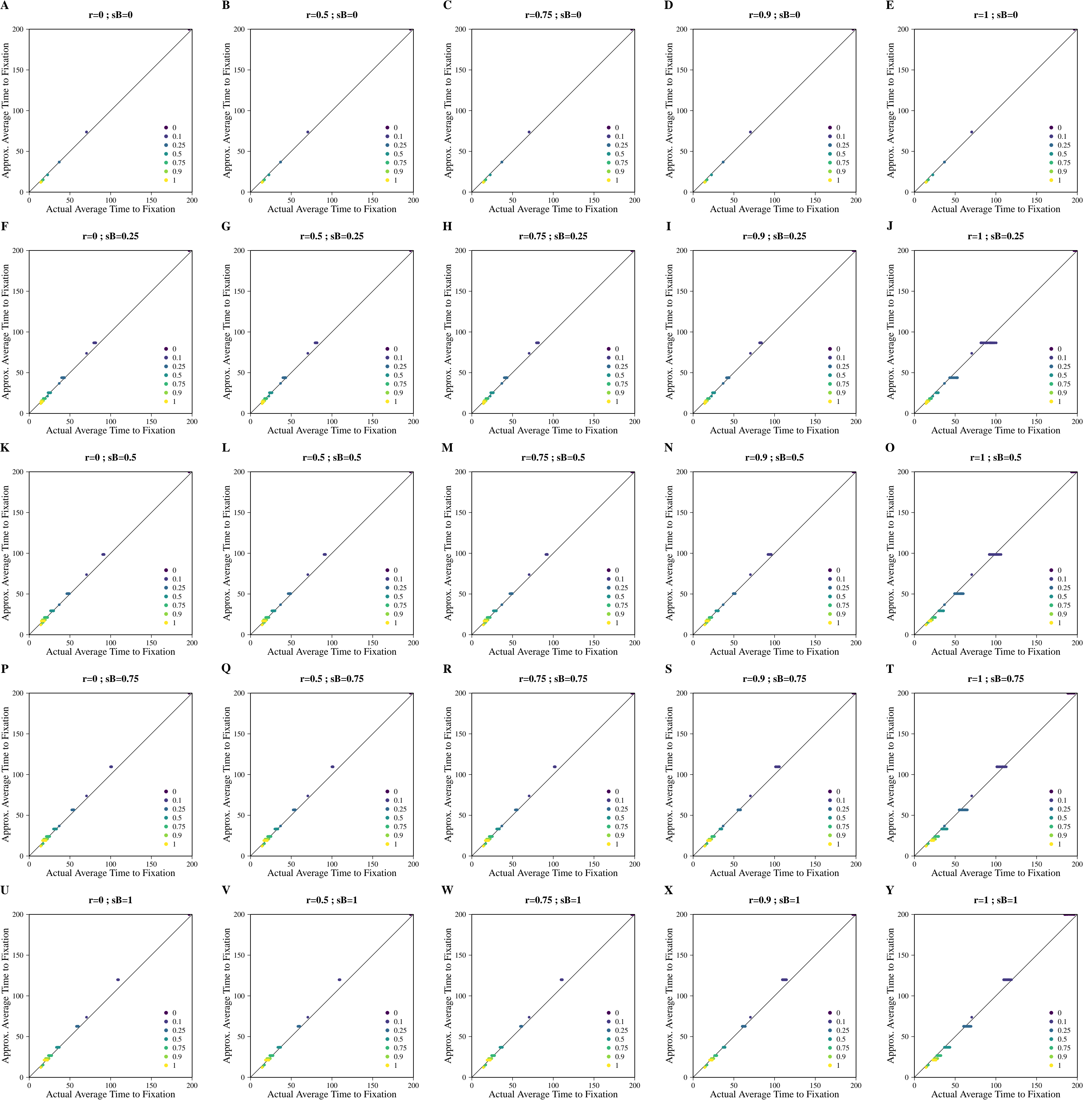

Supplement: Supplementary file 1 — Appendix S1 [file JEB-34-1608-s001.zip › SupportingInformation/FigureS24_COMPARISON_TFIXA_ACTvsAPP_additive_t1000_n100.pdf]

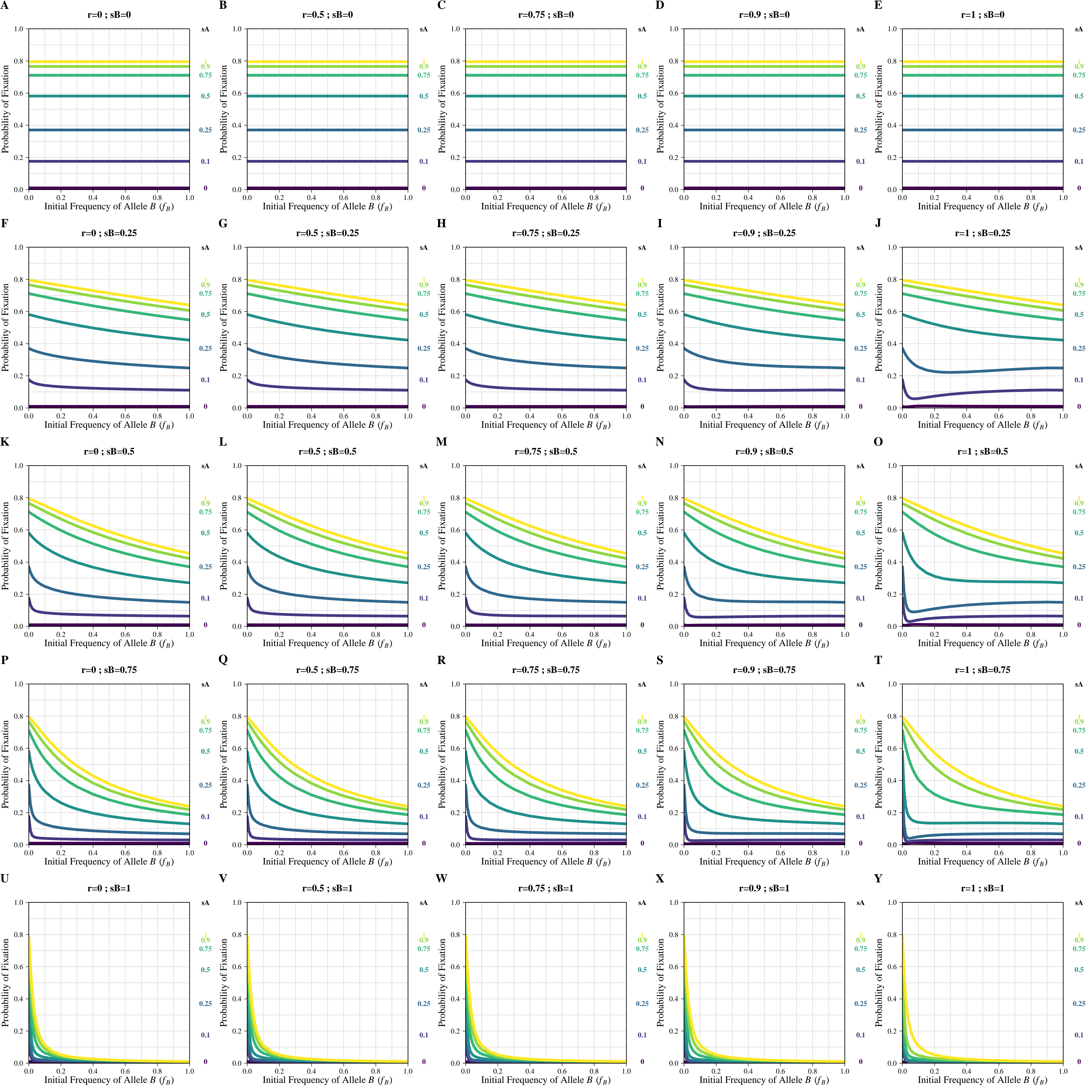

Supplement: Supplementary file 1 — Appendix S1 [file JEB-34-1608-s001.zip › SupportingInformation/FigureS25_PFIXA_stochastic_resistive_t1000_n100_d50.pdf]

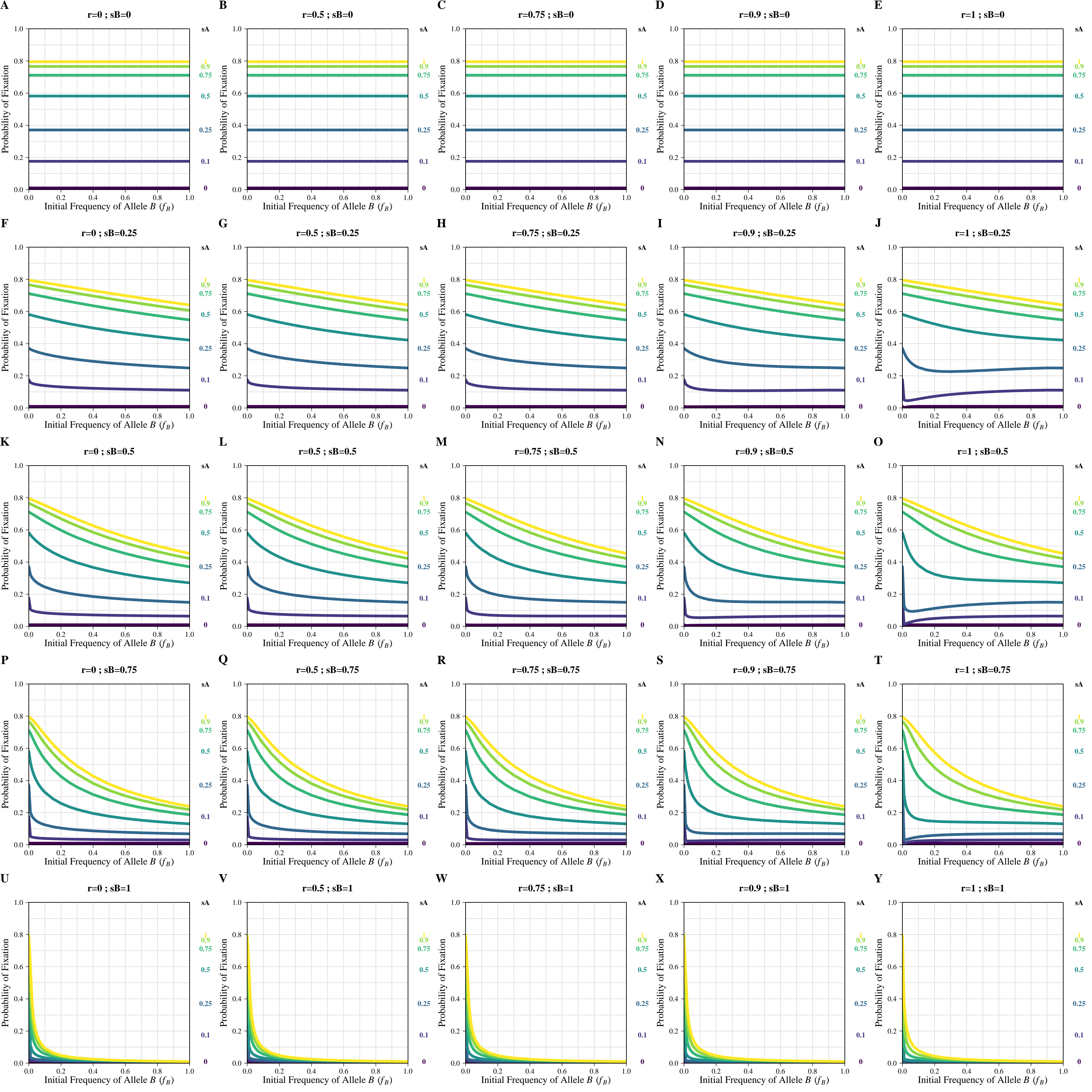

Supplement: Supplementary file 1 — Appendix S1 [file JEB-34-1608-s001.zip › SupportingInformation/FigureS26_PFIXA_deterministic_resistive_t1000_n100_d50.pdf]

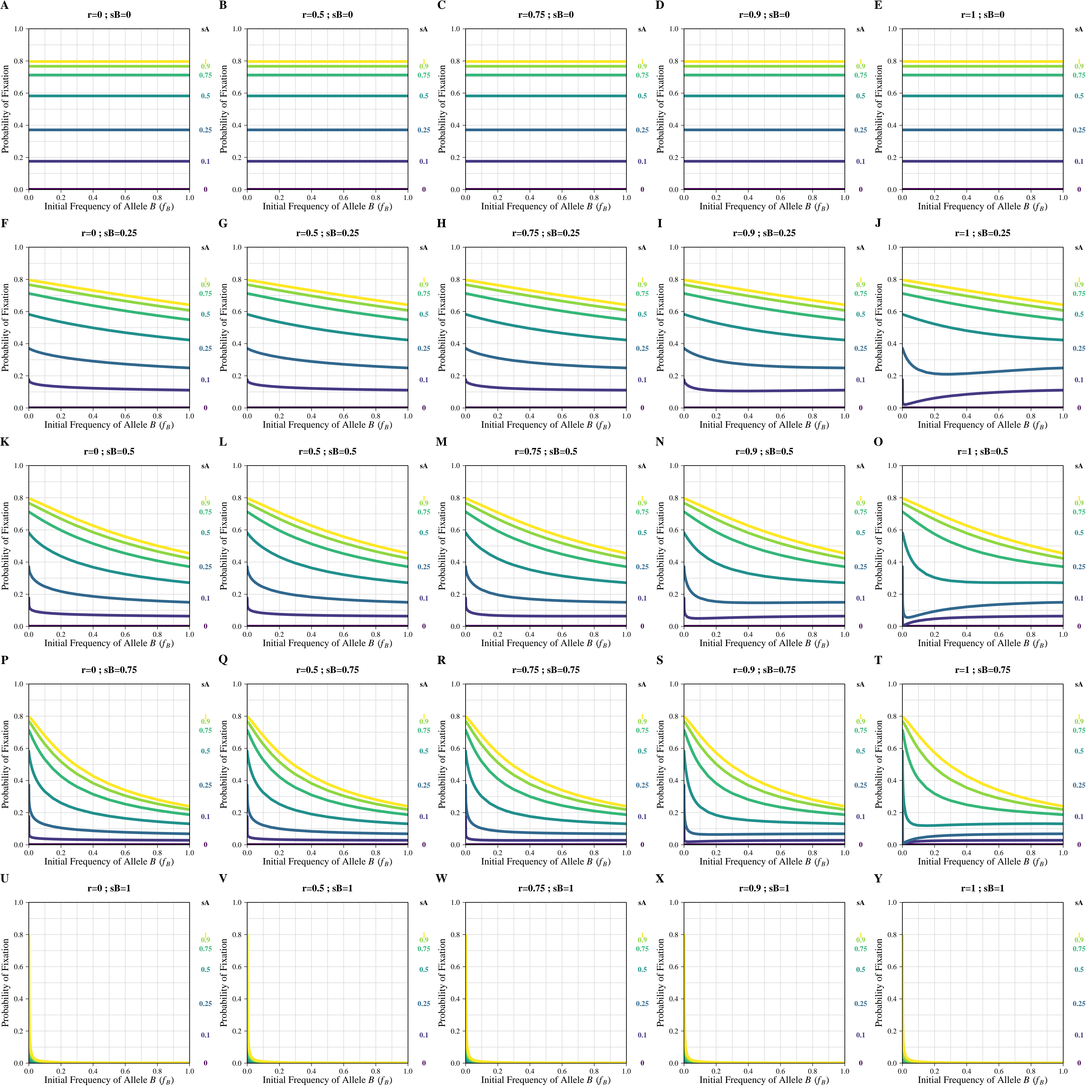

Supplement: Supplementary file 1 — Appendix S1 [file JEB-34-1608-s001.zip › SupportingInformation/FigureS27_PFIXA_deterministic_resistive_t10000_n1000_d50.pdf]

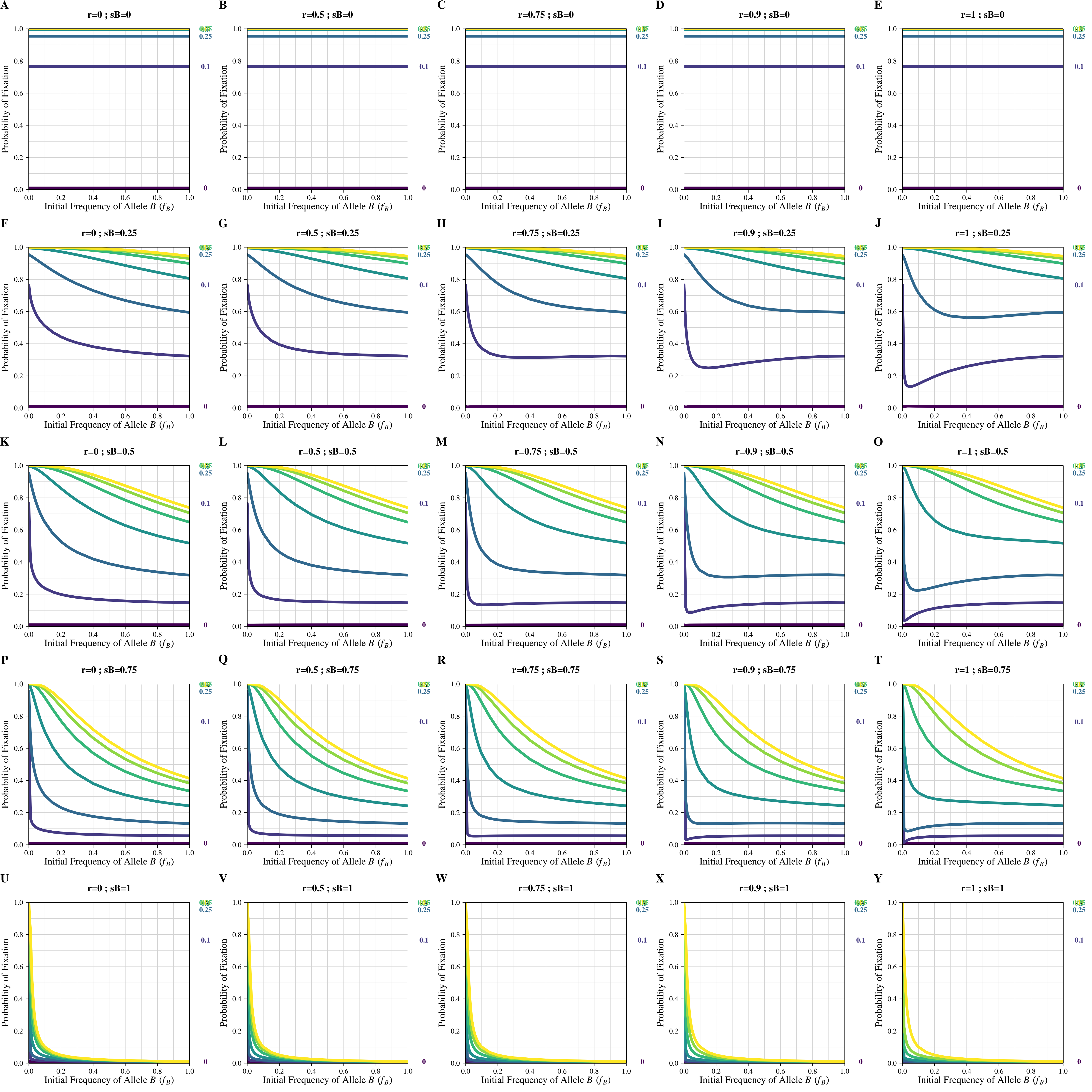

Supplement: Supplementary file 1 — Appendix S1 [file JEB-34-1608-s001.zip › SupportingInformation/FigureS28_PFIXA_stochastic_resistive_t1000_n100_d90.pdf]

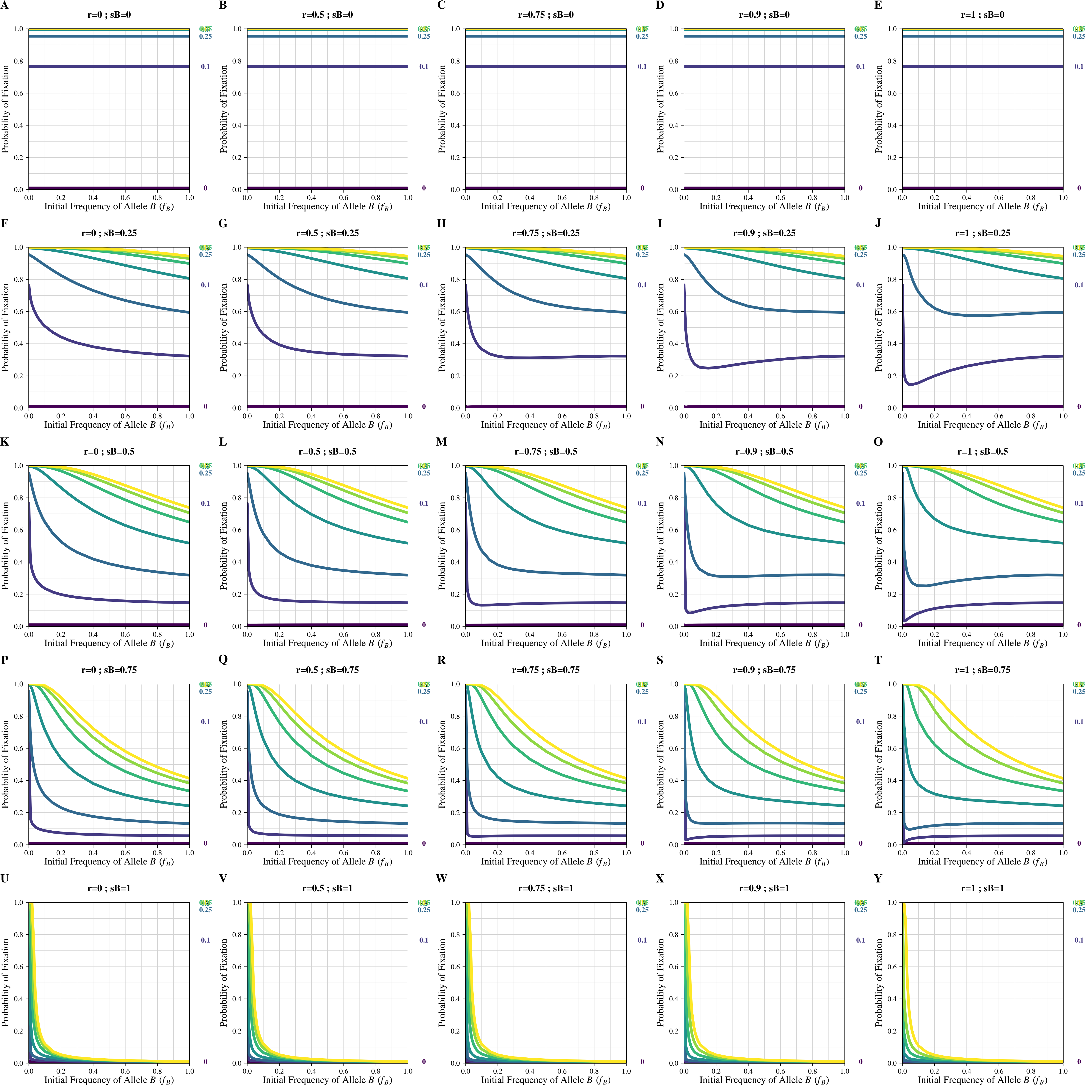

Supplement: Supplementary file 1 — Appendix S1 [file JEB-34-1608-s001.zip › SupportingInformation/FigureS29_PFIXA_deterministic_resistive_t1000_n100_d90.pdf]

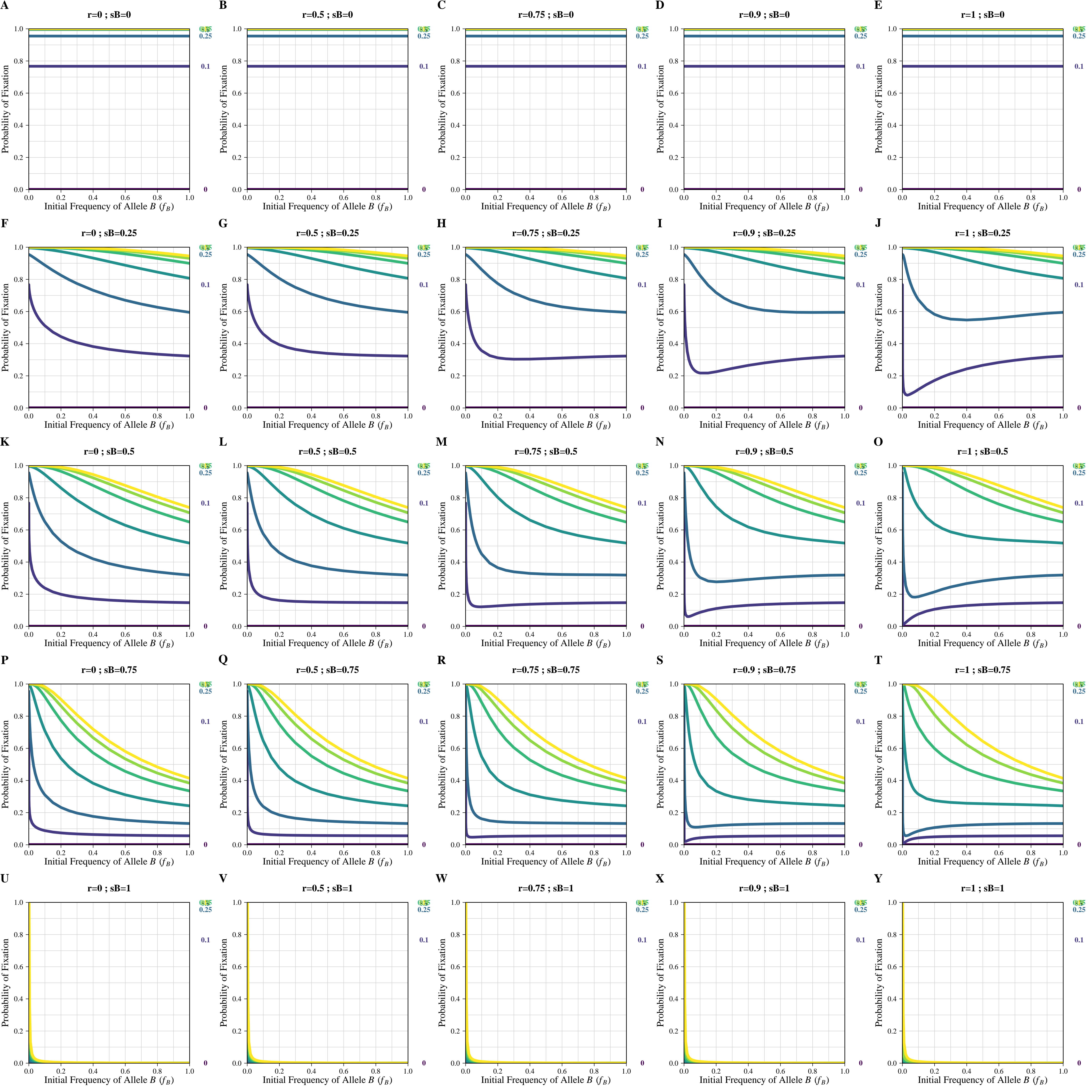

Supplement: Supplementary file 1 — Appendix S1 [file JEB-34-1608-s001.zip › SupportingInformation/FigureS30_PFIXA_stochastic_resistive_t10000_n1000_d90.pdf]

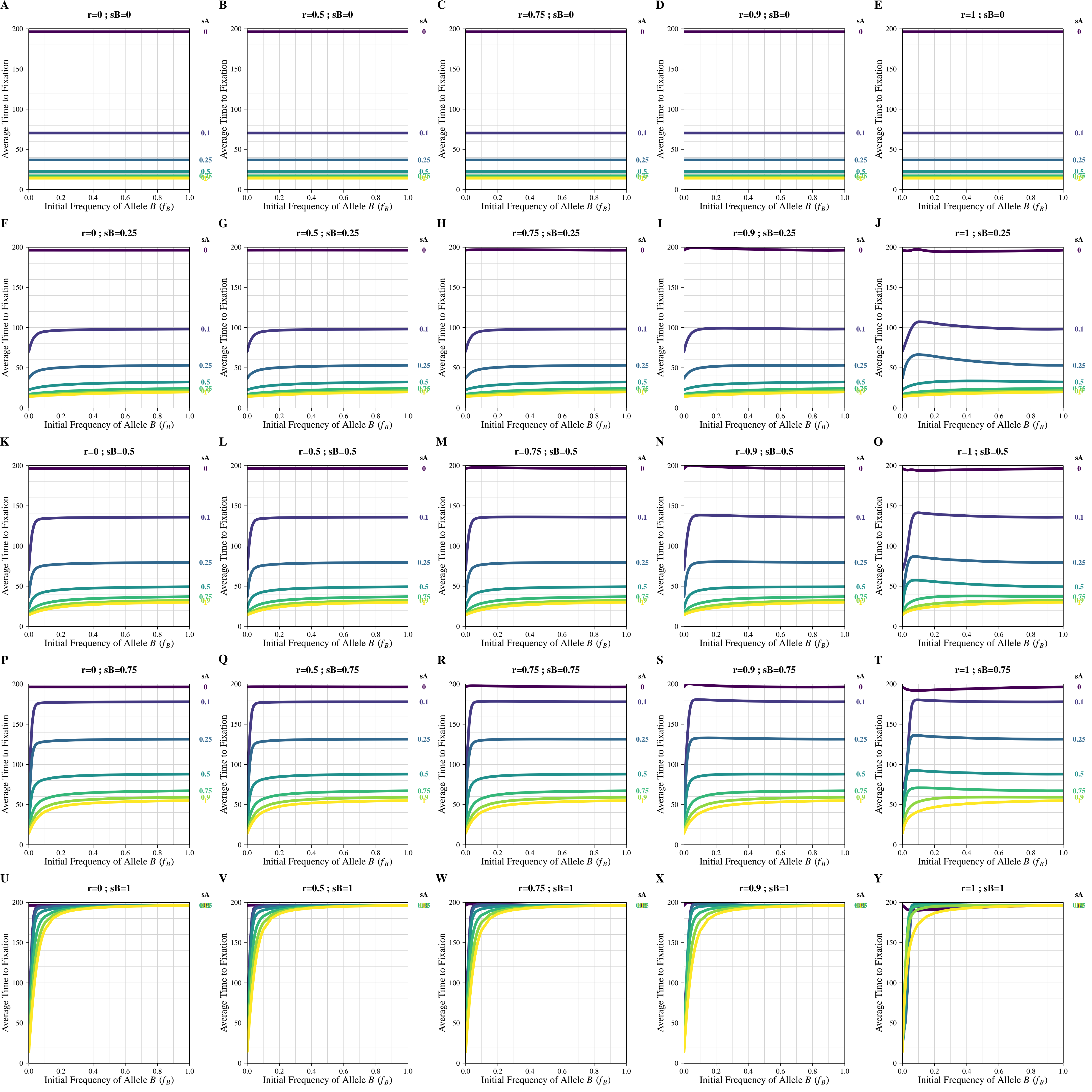

Supplement: Supplementary file 1 — Appendix S1 [file JEB-34-1608-s001.zip › SupportingInformation/FigureS31_TFIXA_stochastic_resistive_t1000_n100_d50.pdf]

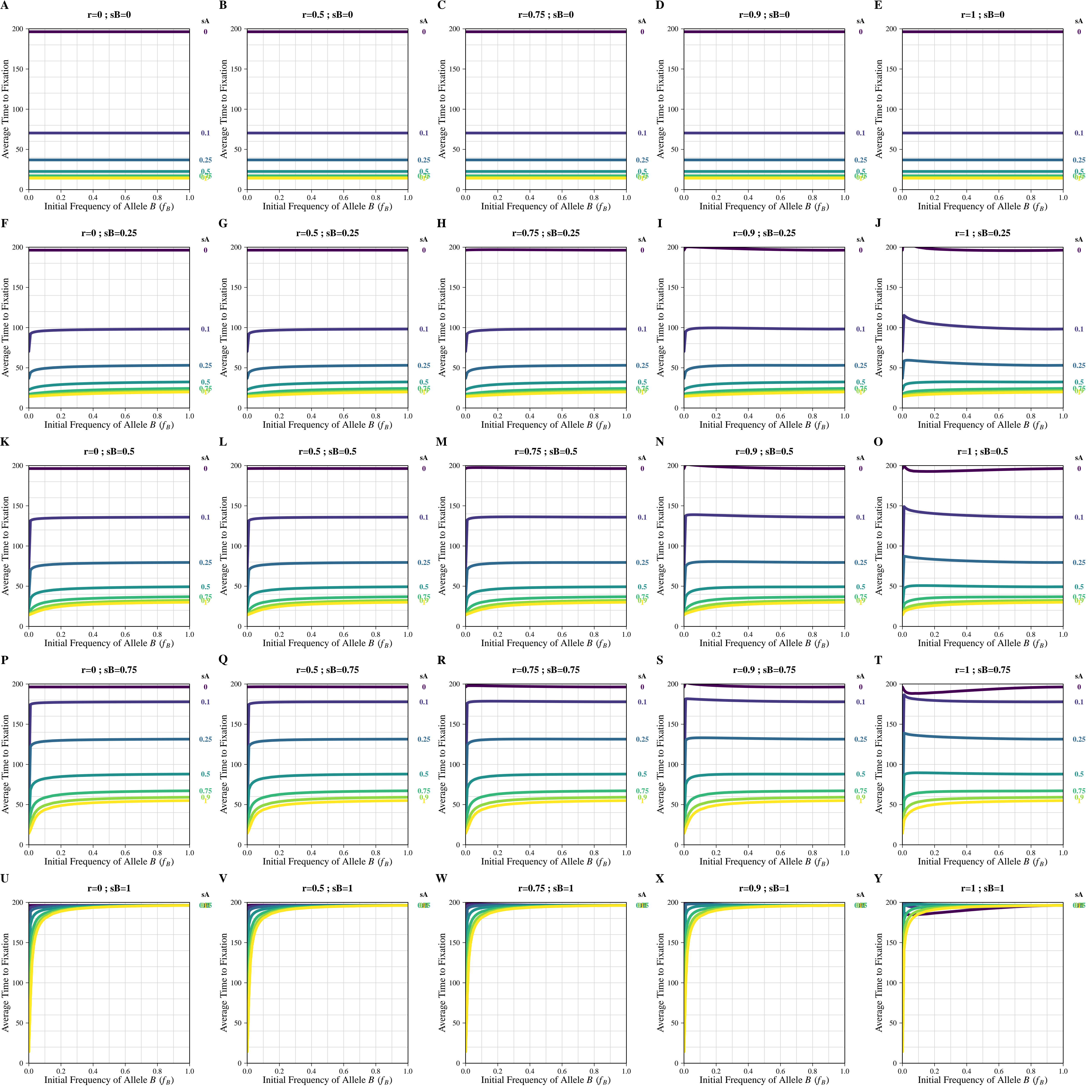

Supplement: Supplementary file 1 — Appendix S1 [file JEB-34-1608-s001.zip › SupportingInformation/FigureS32_TFIXA_deterministic_resistive_t1000_n100_d50.pdf]

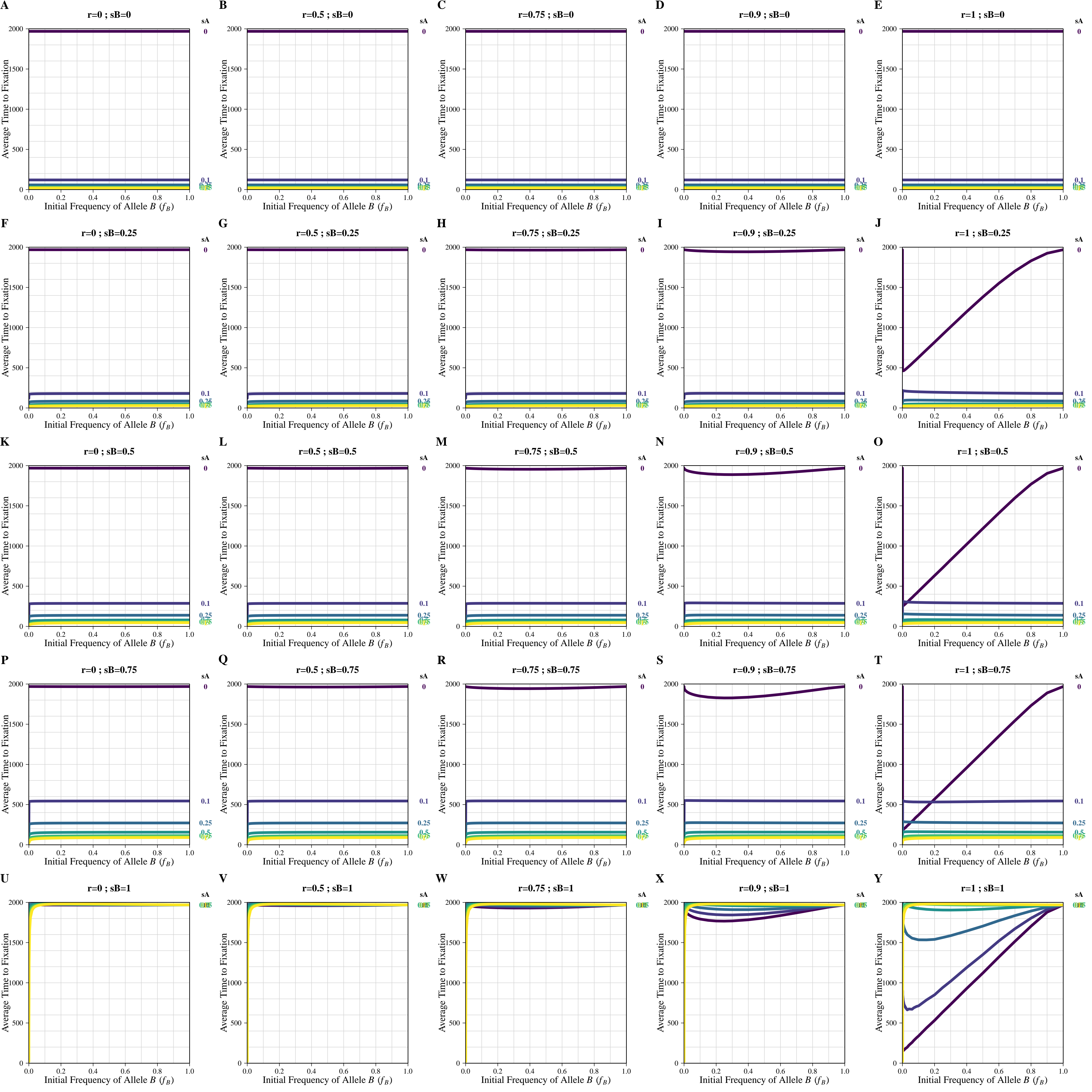

Supplement: Supplementary file 1 — Appendix S1 [file JEB-34-1608-s001.zip › SupportingInformation/FigureS33_TFIXA_deterministic_resistive_t10000_n1000_d50.pdf]

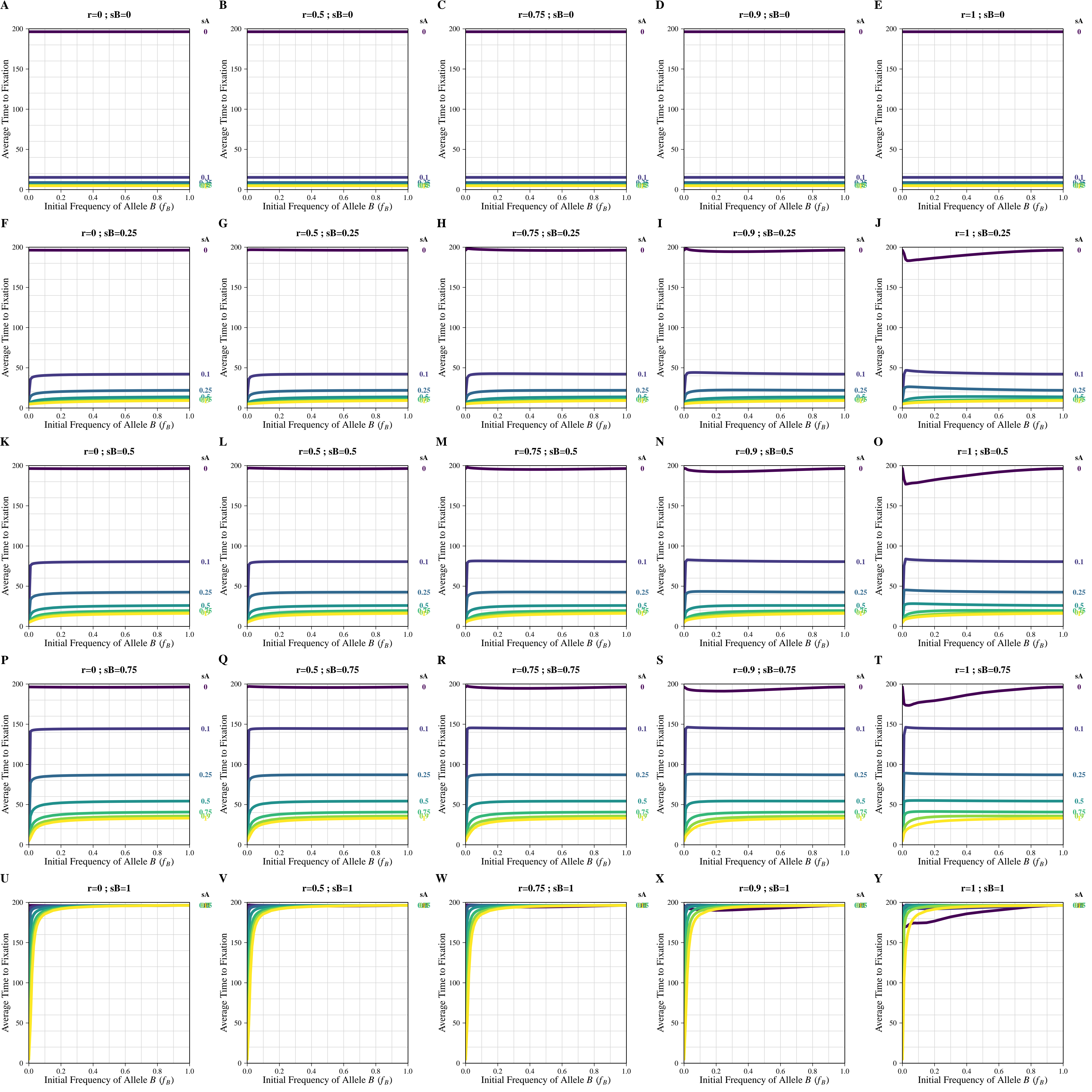

Supplement: Supplementary file 1 — Appendix S1 [file JEB-34-1608-s001.zip › SupportingInformation/FigureS34_TFIXA_stochastic_resistive_t1000_n100_d90.pdf]

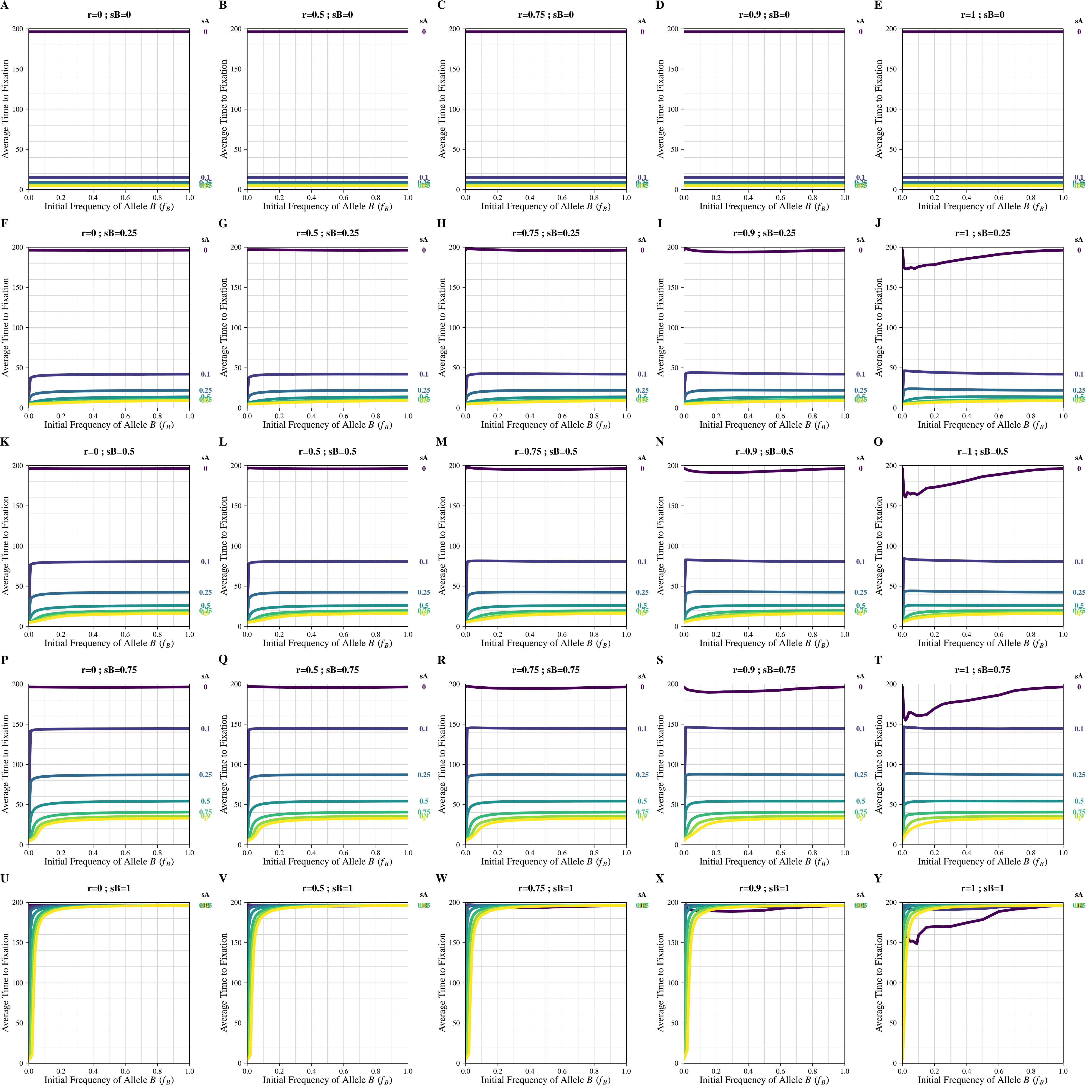

Supplement: Supplementary file 1 — Appendix S1 [file JEB-34-1608-s001.zip › SupportingInformation/FigureS35_TFIXA_deterministic_resistive_t1000_n100_d90.pdf]

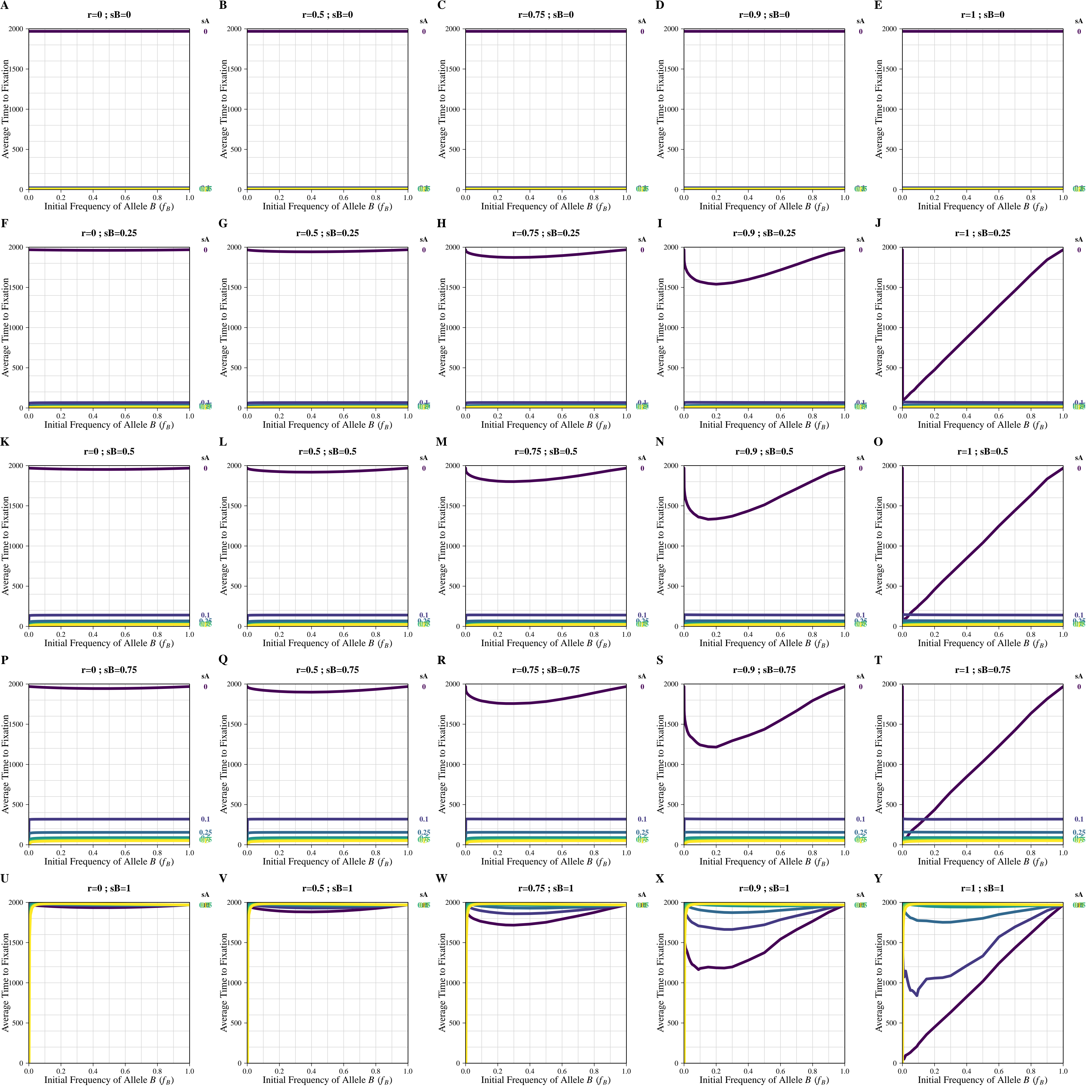

Supplement: Supplementary file 1 — Appendix S1 [file JEB-34-1608-s001.zip › SupportingInformation/FigureS36_TFIXA_deterministic_resistive_t10000_n1000_d90.pdf]

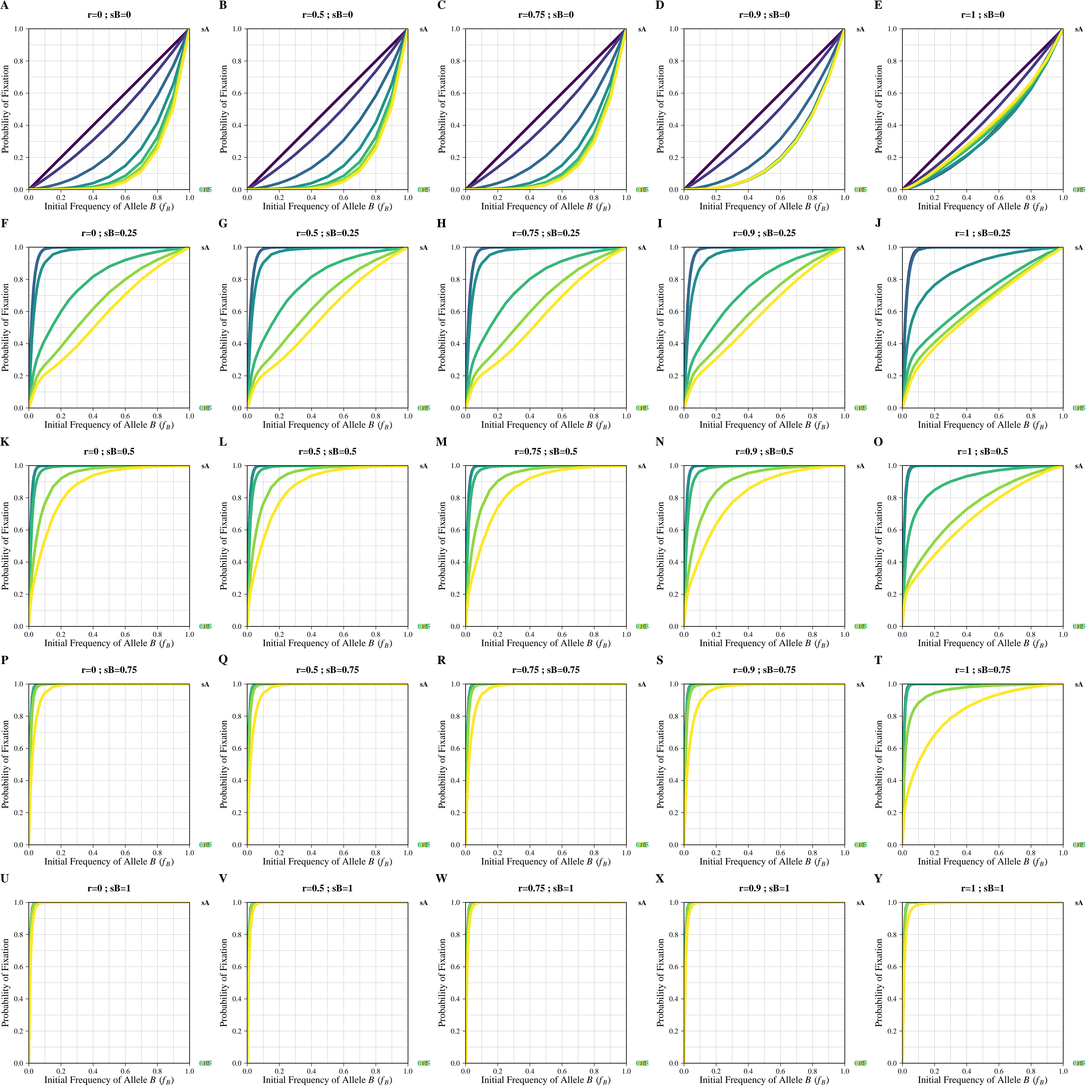

Supplement: Supplementary file 1 — Appendix S1 [file JEB-34-1608-s001.zip › SupportingInformation/FigureS37_PFIXB_stochastic_resistive_t1000_n100_d50.pdf]

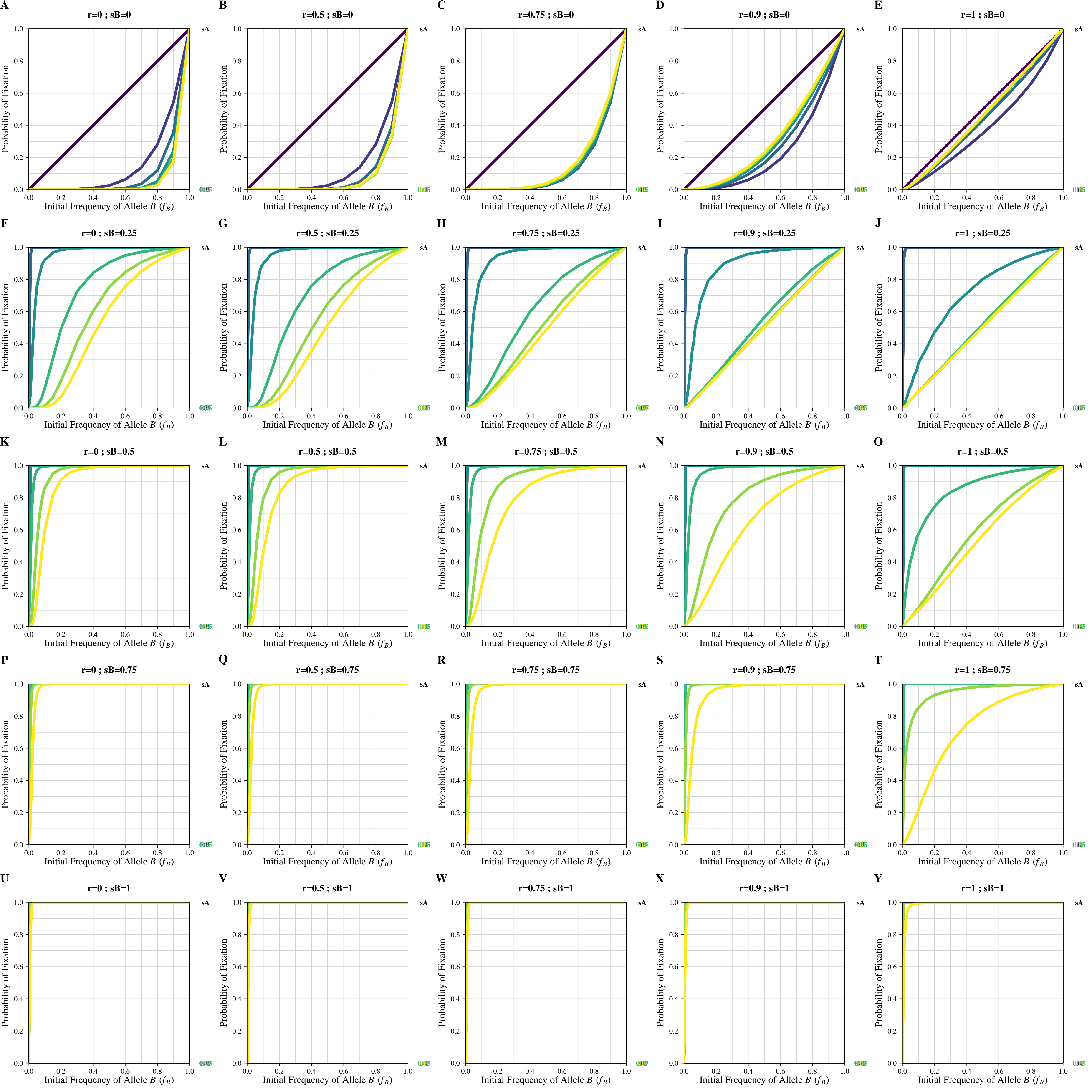

Supplement: Supplementary file 1 — Appendix S1 [file JEB-34-1608-s001.zip › SupportingInformation/FigureS38_PFIXB_stochastic_resistive_t1000_n100_d90.pdf]

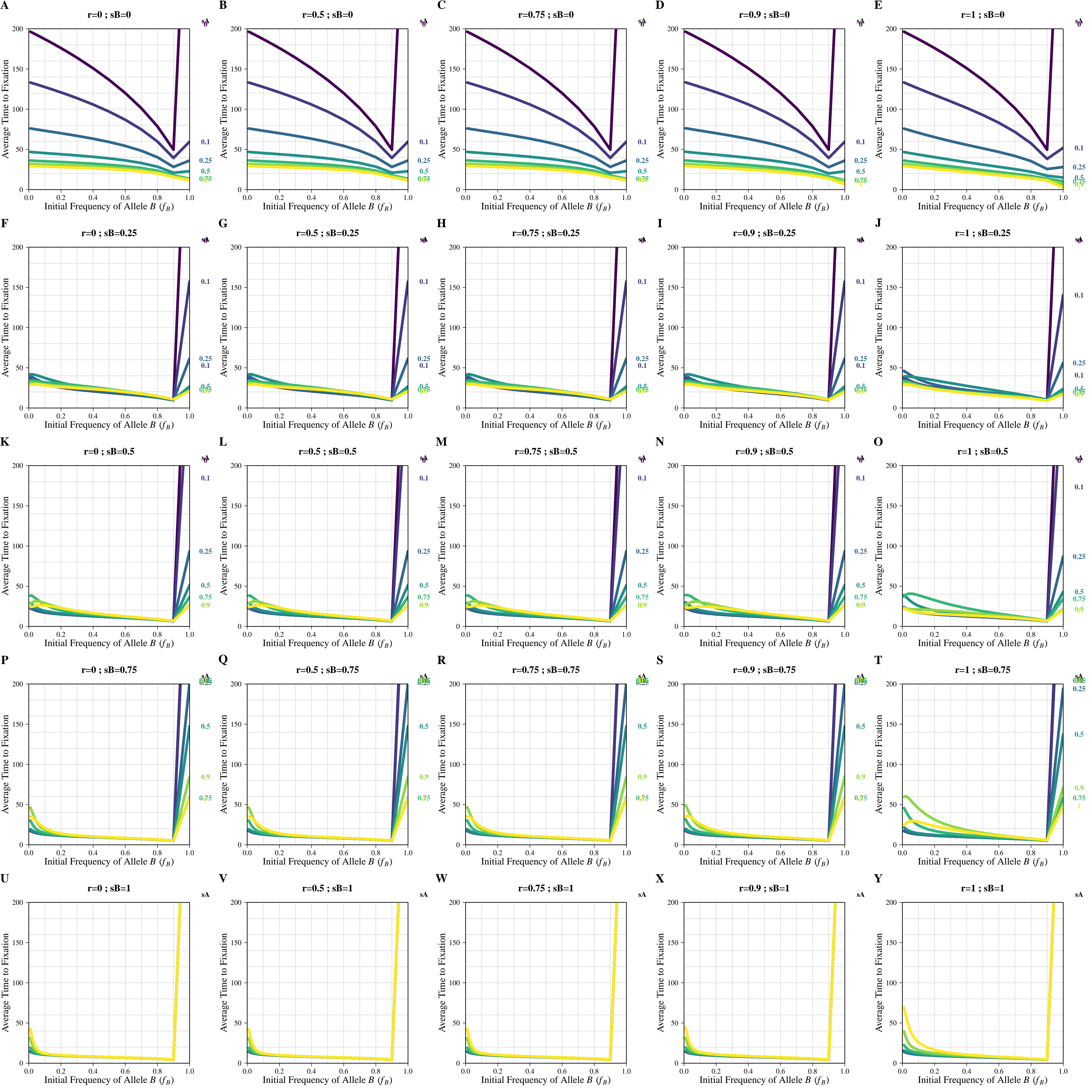

Supplement: Supplementary file 1 — Appendix S1 [file JEB-34-1608-s001.zip › SupportingInformation/FigureS39_TFIXB_stochastic_resistive_t1000_n100_d50.pdf]

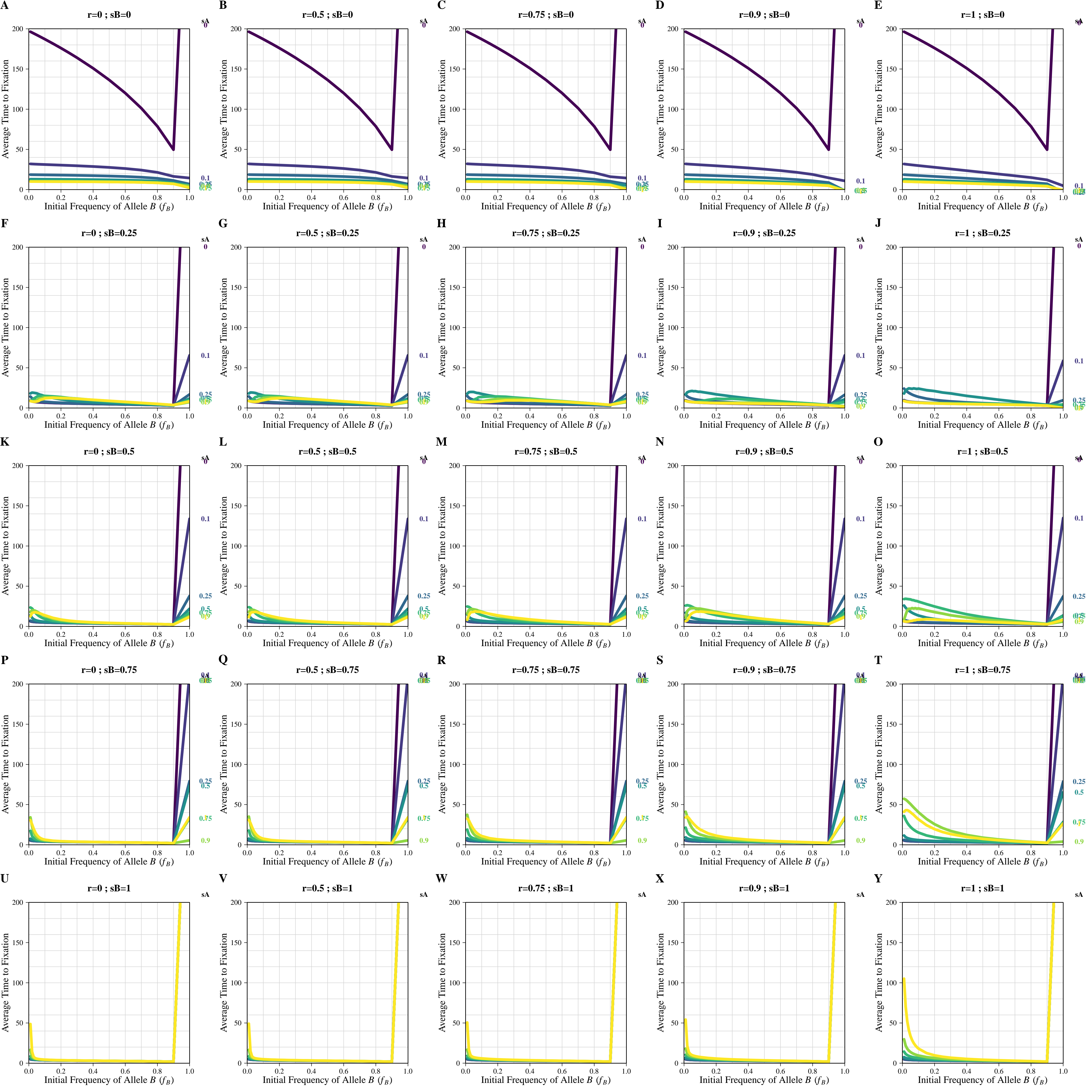

Supplement: Supplementary file 1 — Appendix S1 [file JEB-34-1608-s001.zip › SupportingInformation/FigureS40_TFIXB_stochastic_resistive_t1000_n100_d90.pdf]

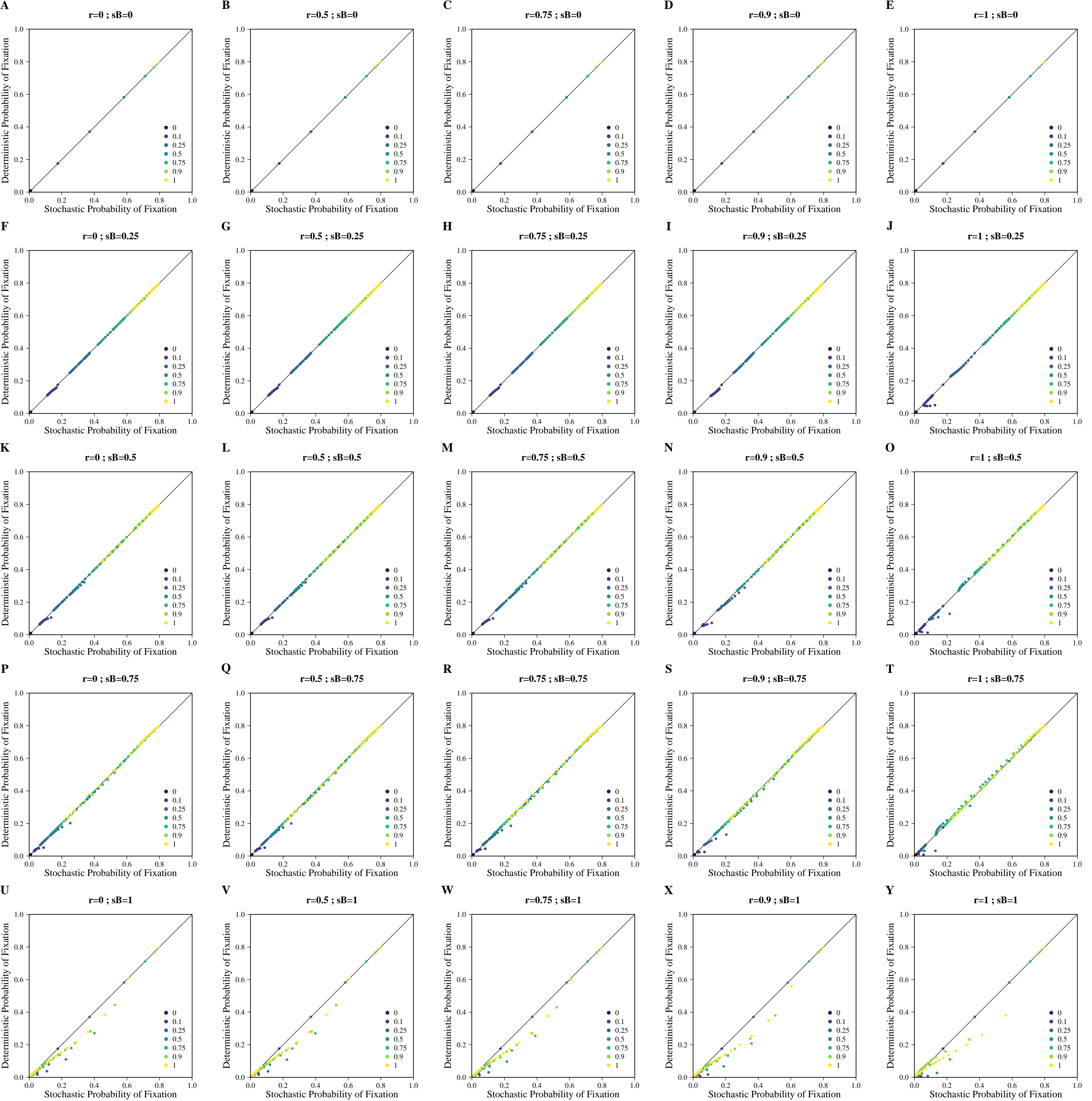

Supplement: Supplementary file 1 — Appendix S1 [file JEB-34-1608-s001.zip › SupportingInformation/FigureS41_COMPARISON_PFIXA_STOvsDET_resistive_t1000_n100_d50.pdf]

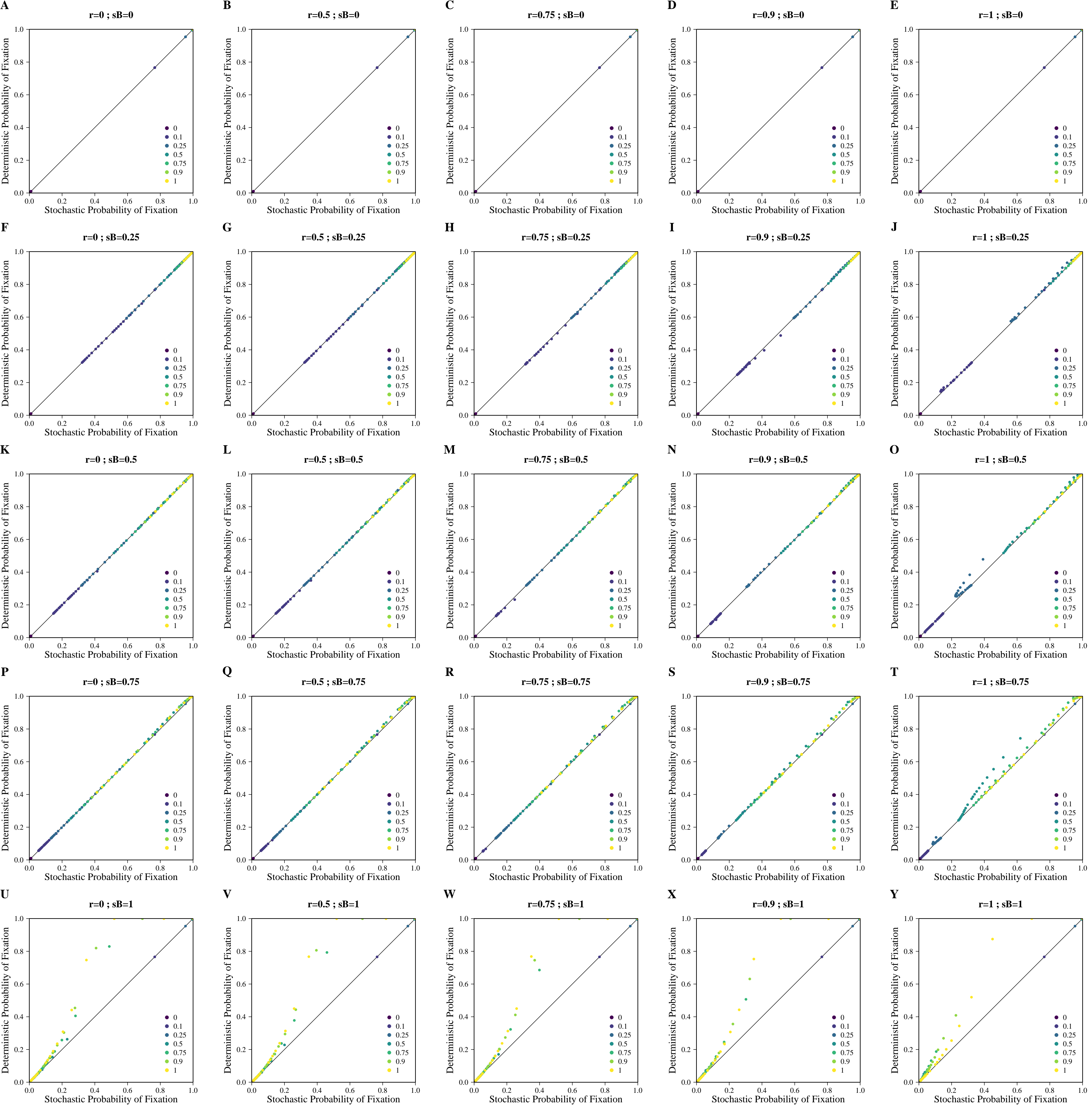

Supplement: Supplementary file 1 — Appendix S1 [file JEB-34-1608-s001.zip › SupportingInformation/FigureS42_COMPARISON_PFIXA_STOvsDET_resistive_t1000_n100_d90.pdf]

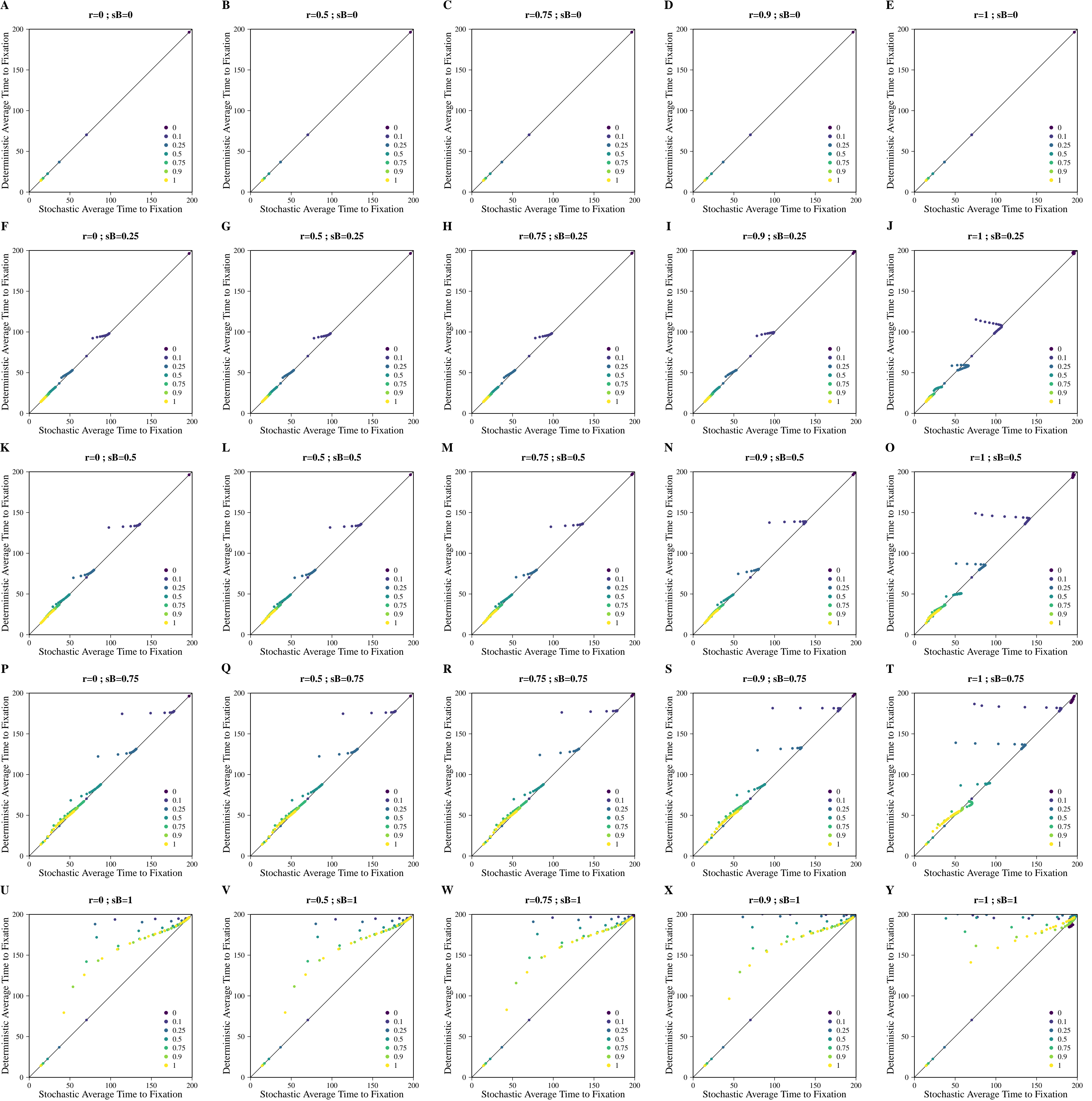

Supplement: Supplementary file 1 — Appendix S1 [file JEB-34-1608-s001.zip › SupportingInformation/FigureS43_COMPARISON_TFIXA_STOvsDET_resistive_t1000_n100_d50.pdf]

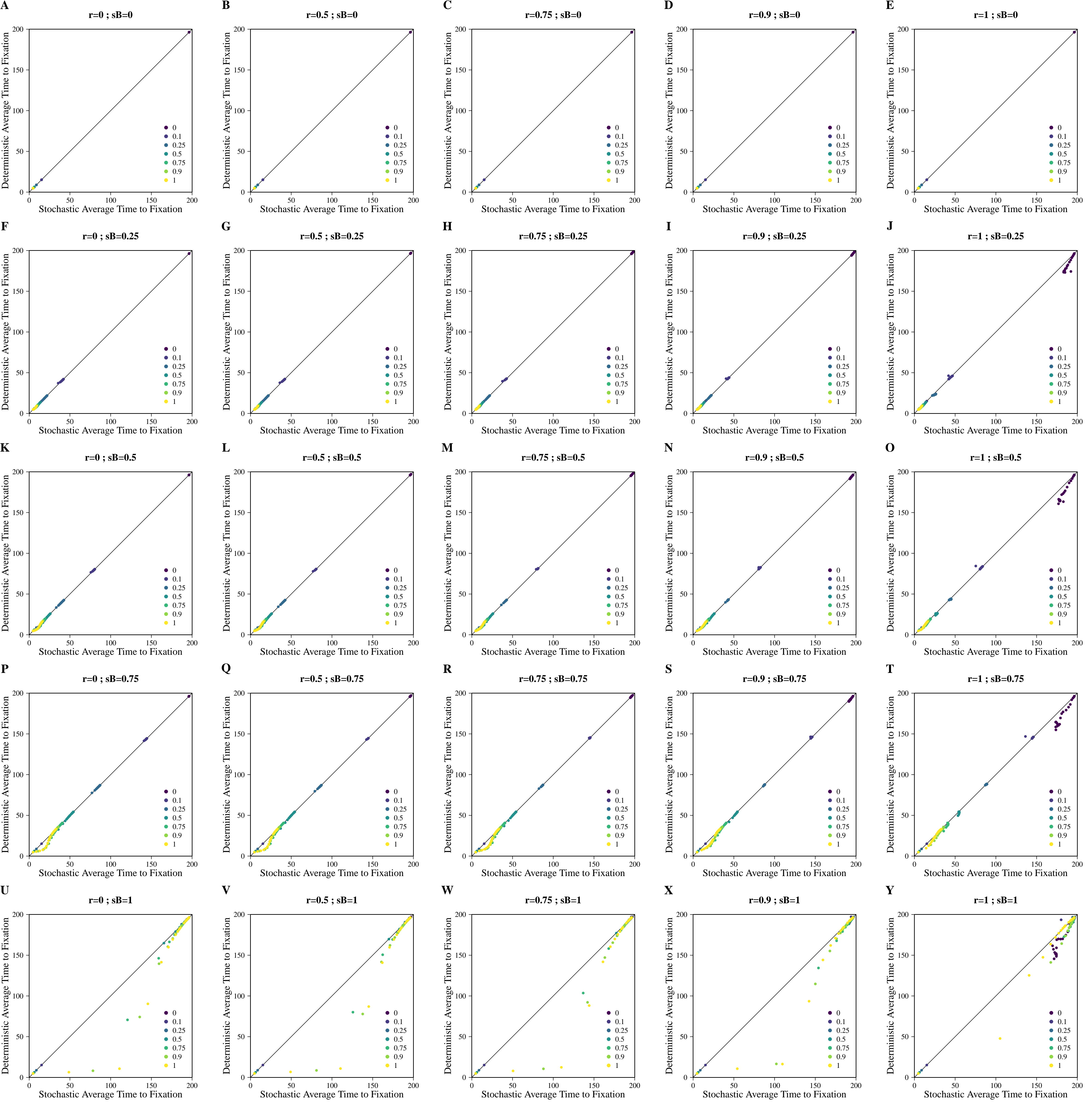

Supplement: Supplementary file 1 — Appendix S1 [file JEB-34-1608-s001.zip › SupportingInformation/FigureS44_COMPARISON_TFIXA_STOvsDET_resistive_t1000_n100_d90.pdf]

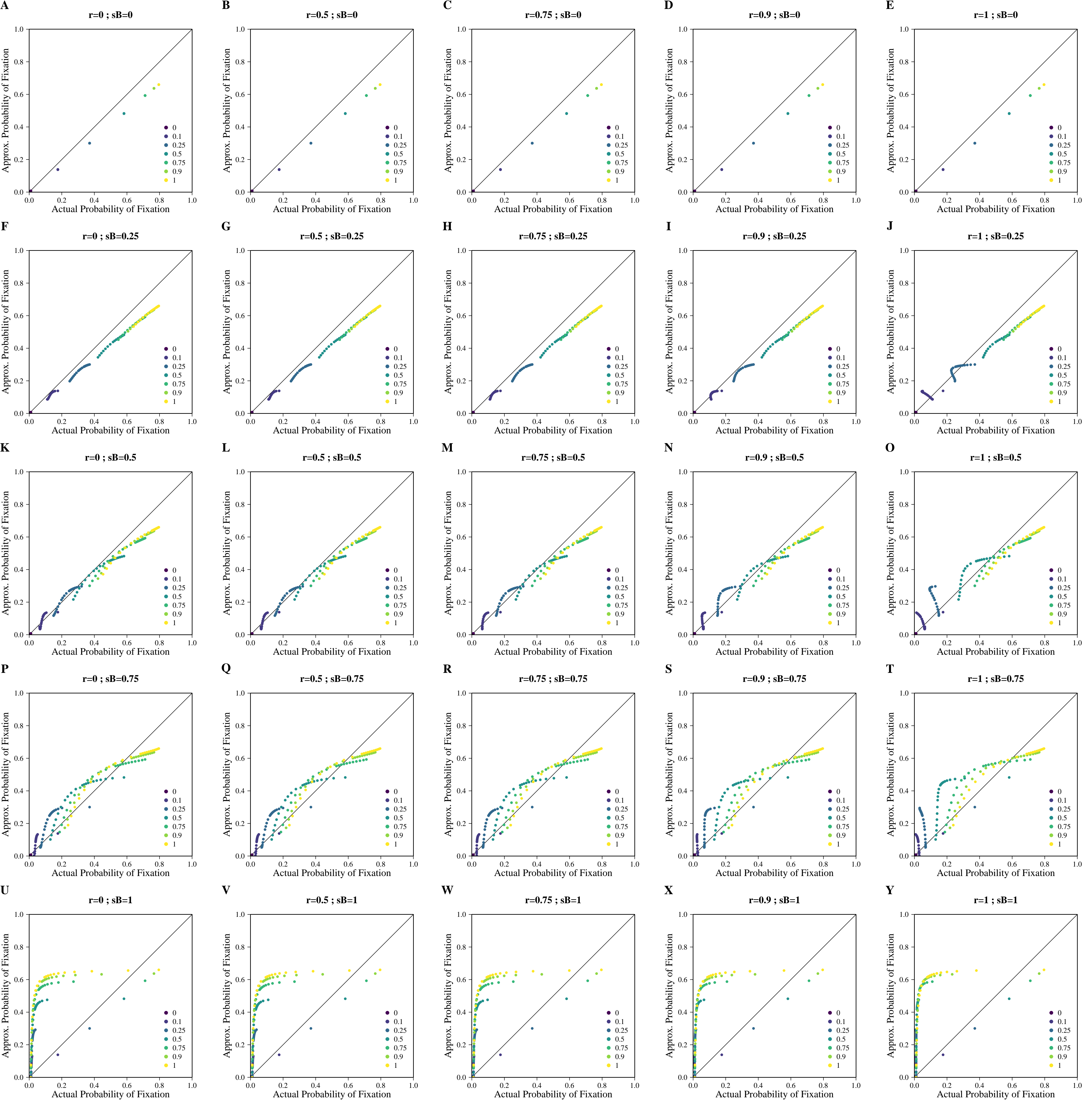

Supplement: Supplementary file 1 — Appendix S1 [file JEB-34-1608-s001.zip › SupportingInformation/FigureS45_COMPARISON_PFIXA_ACTvsAPP_resistive_t1000_n100_d50.pdf]

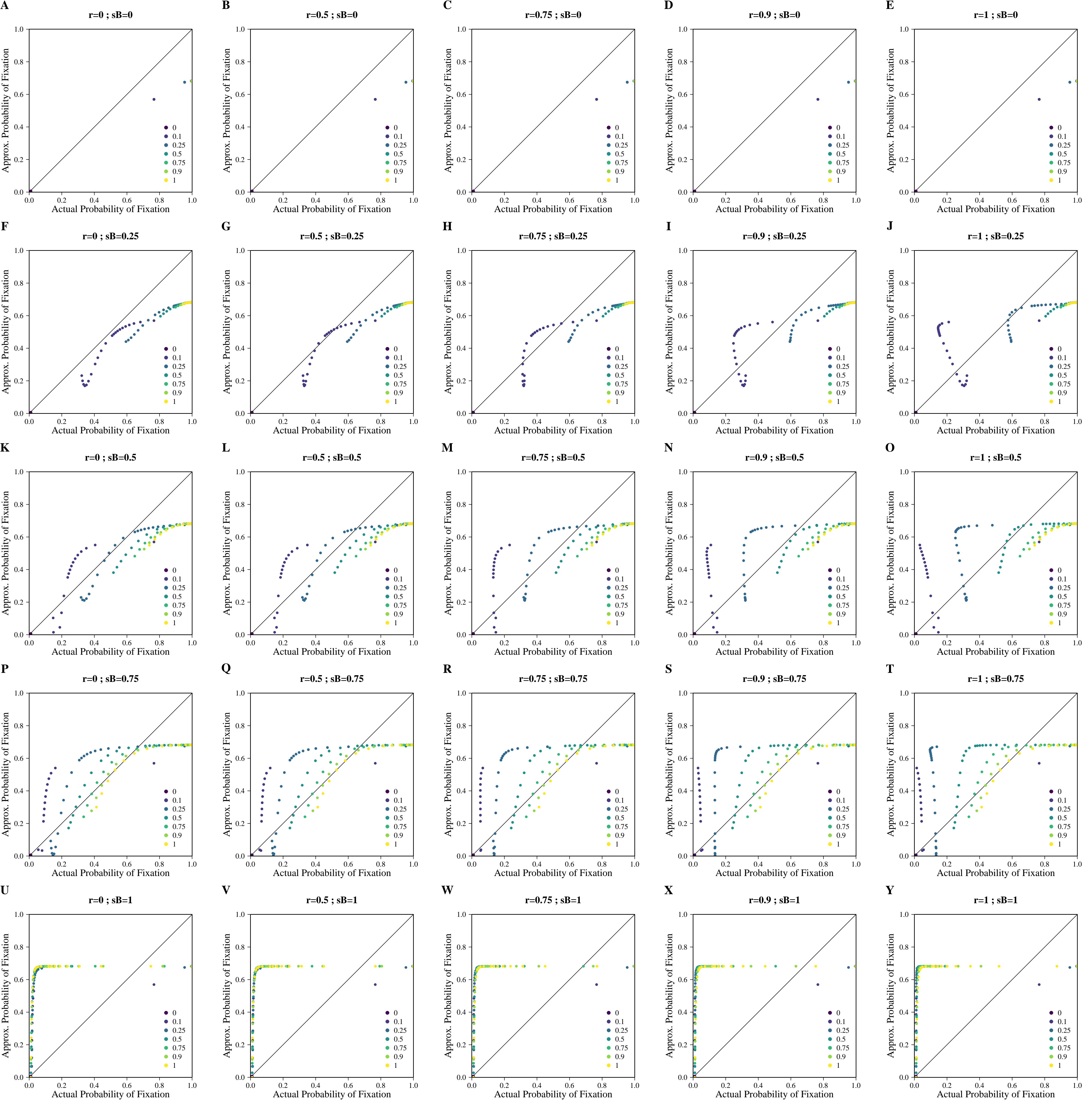

Supplement: Supplementary file 1 — Appendix S1 [file JEB-34-1608-s001.zip › SupportingInformation/FigureS46_COMPARISON_PFIXA_ACTvsAPP_resistive_t1000_n100_d90.pdf]

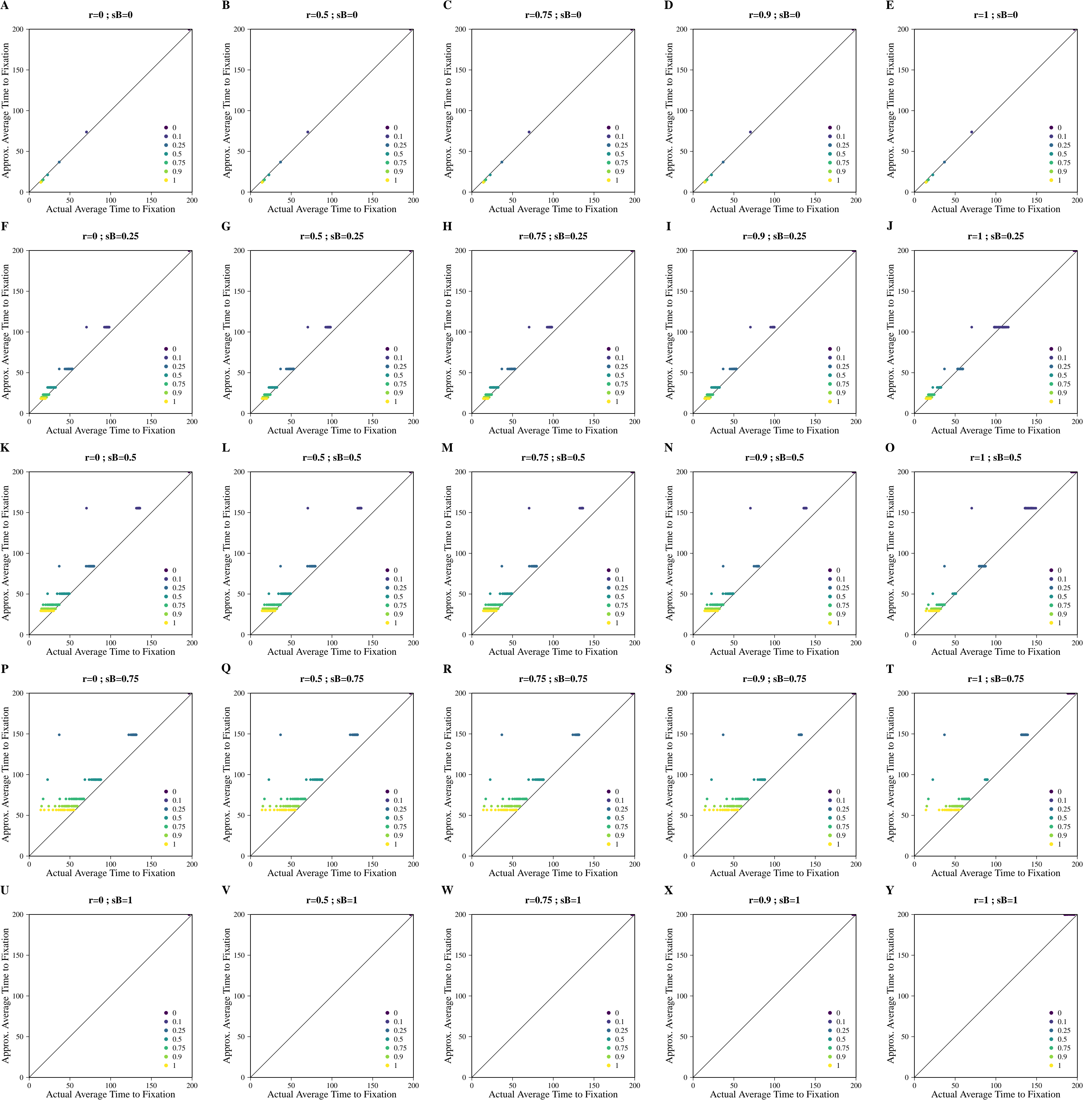

Supplement: Supplementary file 1 — Appendix S1 [file JEB-34-1608-s001.zip › SupportingInformation/FigureS47_COMPARISON_TFIXA_ACTvsAPP_resistive_t1000_n100_d50.pdf]

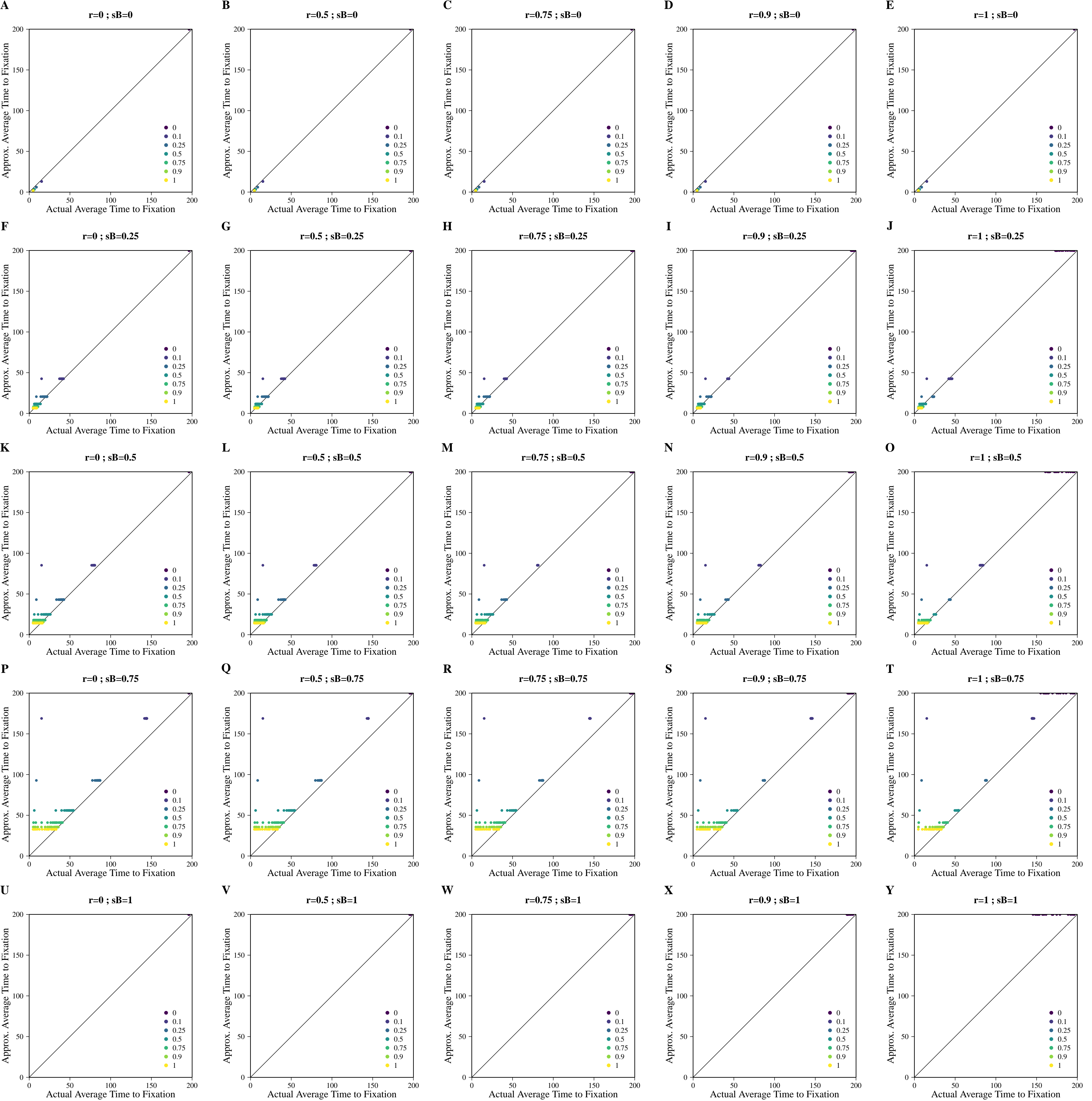

Supplement: Supplementary file 1 — Appendix S1 [file JEB-34-1608-s001.zip › SupportingInformation/FigureS48_COMPARISON_TFIXA_ACTvsAPP_resistive_t1000_n100_d90.pdf]
